# Publisher Correction: Understanding the genetic complexity of puberty timing across the allele frequency spectrum

**DOI:** 10.1038/s41588-024-01857-w

**Published:** 2024-07-09

**Authors:** Katherine A. Kentistou, Lena R. Kaisinger, Stasa Stankovic, Marc Vaudel, Edson Mendes de Oliveira, Andrea Messina, Robin G. Walters, Xiaoxi Liu, Alexander S. Busch, Hannes Helgason, Deborah J. Thompson, Federico Santoni, Konstantin M. Petricek, Yassine Zouaghi, Isabel Huang-Doran, Daniel F. Gudbjartsson, Eirik Bratland, Kuang Lin, Eugene J. Gardner, Yajie Zhao, Raina Y. Jia, Chikashi Terao, Marjorie J. Riggan, Manjeet K. Bolla, Mojgan Yazdanpanah, Nahid Yazdanpanah, Jonathan P. Bradfield, Linda Broer, Archie Campbell, Daniel I. Chasman, Diana L. Cousminer, Nora Franceschini, Lude H. Franke, Giorgia Girotto, Chunyan He, Marjo-Riitta Järvelin, Peter K. Joshi, Yoichiro Kamatani, Robert Karlsson, Jian’an Luan, Kathryn L. Lunetta, Reedik Mägi, Massimo Mangino, Sarah E. Medland, Christa Meisinger, Raymond Noordam, Teresa Nutile, Maria Pina Concas, Ozren Polašek, Eleonora Porcu, Susan M. Ring, Cinzia Sala, Albert V. Smith, Toshiko Tanaka, Peter J. van der Most, Veronique Vitart, Carol A. Wang, Gonneke Willemsen, Marek Zygmunt, Thomas U. Ahearn, Irene L. Andrulis, Hoda Anton-Culver, Antonis C. Antoniou, Paul L. Auer, Catriona L. K. Barnes, Matthias W. Beckmann, Amy Berrington de Gonzalez, Natalia V. Bogdanova, Stig E. Bojesen, Hermann Brenner, Julie E. Buring, Federico Canzian, Jenny Chang-Claude, Fergus J. Couch, Angela Cox, Laura Crisponi, Kamila Czene, Mary B. Daly, Ellen W. Demerath, Joe Dennis, Peter Devilee, Immaculata De Vivo, Thilo Dörk, Alison M. Dunning, Miriam Dwek, Johan G. Eriksson, Peter A. Fasching, Lindsay Fernandez-Rhodes, Liana Ferreli, Olivia Fletcher, Manuela Gago-Dominguez, Montserrat García-Closas, José A. García-Sáenz, Anna González-Neira, Harald Grallert, Pascal Guénel, Christopher A. Haiman, Per Hall, Ute Hamann, Hakon Hakonarson, Roger J. Hart, Martha Hickey, Maartje J. Hooning, Reiner Hoppe, John L. Hopper, Jouke-Jan Hottenga, Frank B. Hu, Hanna Huebner, David J. Hunter, ABCTB Investigators, Helena Jernström, Esther M. John, David Karasik, Elza K. Khusnutdinova, Vessela N. Kristensen, James V. Lacey, Diether Lambrechts, Lenore J. Launer, Penelope A. Lind, Annika Lindblom, Patrik K. E. Magnusson, Arto Mannermaa, Mark I. McCarthy, Thomas Meitinger, Cristina Menni, Kyriaki Michailidou, Iona Y. Millwood, Roger L. Milne, Grant W. Montgomery, Heli Nevanlinna, Ilja M. Nolte, Dale R. Nyholt, Nadia Obi, Katie M. O’Brien, Kenneth Offit, Albertine J. Oldehinkel, Sisse R. Ostrowski, Aarno Palotie, Ole B. Pedersen, Annette Peters, Giulia Pianigiani, Dijana Plaseska-Karanfilska, Anneli Pouta, Alfred Pozarickij, Paolo Radice, Gad Rennert, Frits R. Rosendaal, Daniela Ruggiero, Emmanouil Saloustros, Dale P. Sandler, Sabine Schipf, Carsten O. Schmidt, Marjanka K. Schmidt, Kerrin Small, Beatrice Spedicati, Meir Stampfer, Jennifer Stone, Rulla M. Tamimi, Lauren R. Teras, Emmi Tikkanen, Constance Turman, Celine M. Vachon, Qin Wang, Robert Winqvist, Alicja Wolk, Babette S. Zemel, Wei Zheng, Ko W. van Dijk, Behrooz Z. Alizadeh, Stefania Bandinelli, Eric Boerwinkle, Dorret I. Boomsma, Marina Ciullo, Georgia Chenevix-Trench, Francesco Cucca, Tõnu Esko, Christian Gieger, Struan F. A. Grant, Vilmundur Gudnason, Caroline Hayward, Ivana Kolčić, Peter Kraft, Deborah A. Lawlor, Nicholas G. Martin, Ellen A. Nøhr, Nancy L. Pedersen, Craig E. Pennell, Paul M. Ridker, Antonietta Robino, Harold Snieder, Ulla Sovio, Tim D. Spector, Doris Stöckl, Cathie Sudlow, Nic J. Timpson, Daniela Toniolo, André Uitterlinden, Sheila Ulivi, Henry Völzke, Nicholas J. Wareham, Elisabeth Widen, James F. Wilson, The Lifelines Cohort Study, The Danish Blood Donor Study, The Ovarian Cancer Association Consortium, Esther M. John, The Breast Cancer Association Consortium, Per Hall, Robert Winqvis, The Biobank Japan Project, The China Kadoorie Biobank Collaborative Group, Paul D. P. Pharoah, Liming Li, Douglas F. Easton, Pål R. Njølstad, Patrick Sulem, Joanne M. Murabito, Anna Murray, Despoina Manousaki, Anders Juul, Christian Erikstrup, Kari Stefansson, Momoko Horikoshi, Zhengming Chen, I. Sadaf Farooqi, Nelly Pitteloud, Stefan Johansson, Felix R. Day, John R. B. Perry, Ken K. Ong

**Affiliations:** 1 MRC Epidemiology Unit, University of Cambridge School of Clinical Medicine, Institute of Metabolic Science, Cambridge Biomedical Campus, Cambridge, UK; 2 Mohn Center for Diabetes Precision Medicine, Department of Clinical Science, University of Bergen, Bergen, Norway; 3 Department of Genetics and Bioinformatics, Health Data and Digitalization, Norwegian Institute of Public Health, Oslo, Norway; 4 University of Cambridge Metabolic Research Laboratories and NIHR Cambridge Biomedical Research Centre, Wellcome-MRC Institute of Metabolic Science, Addenbrooke’s Hospital, Cambridge, UK; 5 Division of Endocrinology, Diabetology, and Metabolism, Lausanne University Hospital, Lausanne, Switzerland; 6 Faculty of Biology and Medicine, University of Lausanne, Lausanne, Switzerland; 7 Nuffield Department of Population Health, University of Oxford, Oxford, UK; 8 Laboratory for Statistical and Translational Genetics, RIKEN Center for Integrative Medical Sciences, Yokohama, Japan; 9 Department of General Pediatrics, University of Münster, Münster, Germany; 10 Department of Growth and Reproduction, Copenhagen University Hospital—Rigshospitalet, Copenhagen, Denmark; 11 deCODE Genetics/Amgen, Inc., Reykjavik, Iceland; 12 School of Engineering and Natural Sciences, University of Iceland, Reykjavik, Iceland; 13 Centre for Cancer Genetic Epidemiology, Department of Public Health and Primary Care, University of Cambridge, Cambridge, UK; 14 Charité Universitätsmedizin Berlin, Corporate Member of Freie Universität Berlin and Humboldt-Universität zu Berlin, Institute of Pharmacology, Berlin, Germany; 15 Department of Medical Genetics, Haukeland University Hospital, Bergen, Norway; 16 Clinical Research Center, Shizuoka General Hospital, Shizuoka, Japan; 17 Department of Applied Genetics, The School of Pharmaceutical Sciences, University of Shizuoka, Shizuoka, Japan; 18 Department of Gynecology, Duke University Medical Center, Durham, NC USA; 19 Research Center of the Sainte-Justine University Hospital, University of Montreal, Montreal, Quebec Canada; 20 Quantinuum Research, Wayne, PA USA; 21 Center for Applied Genomics, Children’s Hospital of Philadelphia, Philadelphia, PA USA; 22 Department of Internal Medicine, Erasmus MC, Rotterdam, The Netherlands; 23 Department of Epidemiology, Erasmus MC, Rotterdam, The Netherlands; 24 Centre for Genomic and Experimental Medicine, Institute of Genetics and Cancer, University of Edinburgh, Edinburgh, UK; 25 Division of Preventive Medicine, Brigham and Women’s Hospital and Harvard Medical School, Boston, MA USA; 26 Division of Human Genetics, Children’s Hospital of Philadelphia, Philadelphia, PA USA; 27 Department of Genetics, University of Pennsylvania, Philadelphia, PA USA; 28 Center for Spatial and Functional Genomics, Children’s Hospital of Philadelphia, Philadelphia, PA USA; 29 Department of Epidemiology, University of North Carolina, Chapel Hill, NC USA; 30 Department of Genetics, University of Groningen, University Medical Center Groningen, Groningen, The Netherlands; 31 Institute for Maternal and Child Health—IRCCS ‘Burlo Garofolo’, Trieste, Italy; 32 Department of Medicine, Surgery and Health Sciences, University of Trieste, Trieste, Italy; 33 Department of Internal Medicine, Division of Medical Oncology, University of Kentucky College of Medicine, Lexington, KY USA; 34 Cancer Prevention and Control Research Program, Markey Cancer Center, University of Kentucky, Lexington, KY USA; 35 Department of Epidemiology and Biostatistics, MRC Health Protection Agency (HPA) Centre for Environment and Health, School of Public Health, Imperial College London, London, UK; 36 Institute of Health Sciences, University of Oulu, Oulu, Finland; 37 Biocenter Oulu, University of Oulu, Oulu, Finland; 38 Unit of Primary Care, Oulu University Hospital, Oulu, Finland; 39 Department of Children and Young People and Families, National Institute for Health and Welfare, Oulu, Finland; 40 Centre for Global Health Research, Usher Institute, University of Edinburgh, Edinburgh, Scotland; 41 Laboratory of Complex Trait Genomics, Department of Computational Biology and Medical Sciences, Graduate School of Frontier Sciences, The University of Tokyo, Tokyo, Japan; 42 Department of Medical Epidemiology and Biostatistics, Karolinska Institutet, Stockholm, Sweden; 43 Department of Biostatistics, Boston University School of Public Health, Boston, MA USA; 44 NHLBI’s and Boston University’s Framingham Heart Study, Framingham, MA USA; 45 Institute of Genomics, University of Tartu, Tartu, Estonia; 46 Department of Twin Research and Genetic Epidemiology, King’s College London, London, UK; 47 NIHR Biomedical Research Centre at Guy’s and St. Thomas’ Foundation Trust, London, UK; 48 QIMR Berghofer Medical Research Institute, Brisbane, Queensland Australia; 49 School of Psychology, University of Queensland, Brisbane, Queensland Australia; 50 Faculty of Medicine, University of Queensland, Brisbane, Queensland Australia; 51 Epidemiology, Medical Faculty, University of Augsburg, University Hospital of Augsburg, Augsburg, Germany; 52 Department of Internal Medicine, Section of Gerontology and Geriatrics, Leiden University Medical Center, Leiden, The Netherlands; 53 Institute of Genetics and Biophysics ‘A. Buzzati-Traverso’, CNR, Naples, Italy; 54 University of Split School of Medicine, Split, Croatia; 55 Algebra University College, Zagreb, Croatia; 56 Institute of Genetics and Biomedical Research, National Research Council, Sardinia, Italy; 57 Department of Biomedical Sciences, University of Sassari, Sassari, Italy; 58 MRC Integrative Epidemiology Unit, University of Bristol, Bristol, UK; 59 Population Health Science, Bristol Medical School, University of Bristol, Bristol, UK; 60 Division of Genetics and Cell Biology, San Raffele Hospital, Milano, Italy; 61 Icelandic Heart Association, Kopavogur, Iceland; 62 Faculty of Medicine, University of Iceland, Reykjavik, Iceland; 63 National Institute on Aging, National Institutes of Health, Baltimore, MD USA; 64 Department of Epidemiology, University of Groningen, University Medical Center Groningen, Groningen, The Netherlands; 65 MRC Human Genetics Unit, Institute of Genetics and Cancer, University of Edinburgh, Edinburgh, UK; 66 School of Medicine and Public Health, University of Newcastle, Newcastle, New South Wales Australia; 67 Hunter Medical Research Institute, Newcastle, New South Wales Australia; 68 Department of Biological Psychology, Vrije Universiteit Amsterdam; Amsterdam Public Health (APH) Research Institute, Amsterdam, The Netherlands; 69 Clinic of Gynaecology and Obstetrics, University Medicine Greifswald, Greifswald, Germany; 70 Division of Cancer Epidemiology and Genetics National Cancer Institute, National Institutes of Health, Department of Health and Human Services Bethesda, Bethesda, MD USA; 71 Fred A. Litwin Center for Cancer Genetics, Lunenfeld-Tanenbaum Research Institute of Mount Sinai Hospital, Toronto, Ontario Canada; 72 Department of Molecular Genetics, University of Toronto, Toronto, Ontario Canada; 73 Department of Medicine, Genetic Epidemiology Research Institute, University of California Irvine, Irvine, CA USA; 74 Division of Biostatistics, Institute for Health and Equity and Cancer Center, Medical College of Wisconsin, Milwaukee, WI USA; 75 Department of Gynecology and Obstetrics, Comprehensive Cancer Center Erlangen-EMN, Friedrich-Alexander University Erlangen-Nuremberg, University Hospital Erlangen, Erlangen, Germany; 76 Division of Genetics and Epidemiology, The Institute of Cancer Research, London, UK; 77 Department of Radiation Oncology, Hannover Medical School, Hannover, Germany; 78 Gynaecology Research Unit, Hannover Medical School, Hannover, Germany; 79 N.N. Alexandrov Research Institute of Oncology and Medical Radiology, Minsk, Belarus; 80 Copenhagen General Population Study, Herlev and Gentofte Hospital Copenhagen University Hospital, Herlev, Denmark; 81 Department of Clinical Biochemistry, Herlev and Gentofte Hospital Copenhagen University Hospital, Herlev, Denmark; 82 Division of Clinical Epidemiology and Aging Research, German Cancer Research Center (DKFZ), Heidelberg, Germany; 83 Division of Preventive Oncology, German Cancer Research Center (DKFZ) and National Center for Tumor Diseases (NCT), Heidelberg, Germany; 84 German Cancer Consortium (DKTK), German Cancer Research Center (DKFZ), Heidelberg, Germany; 85 Genomic Epidemiology Group, German Cancer Research Center (DKFZ), Heidelberg, Germany; 86 Division of Cancer Epidemiology, German Cancer Research Center (DKFZ), Heidelberg, Germany; 87 Cancer Epidemiology Group, University Cancer Center Hamburg (UCCH), University Medical Center Hamburg-Eppendorf, Hamburg, Germany; 88 Department of Laboratory Medicine and Pathology, Mayo Clinic Rochester, Rochester, MN USA; 89 Sheffield Institute for Nucleic Acids (SInFoNiA), Department of Oncology and Metabolism, University of Sheffield, Sheffield, UK; 90 Department of Clinical Genetics, Fox Chase Cancer Center, Philadelphia, PA USA; 91 Division of Epidemiology and Community Health, School of Public Health, University of Minnesota, Minneapolis, MN USA; 92 Department of Pathology, Leiden University Medical Center, Leiden, The Netherlands; 93 Department of Human Genetics, Leiden University Medical Center, Leiden, The Netherlands; 94 Department of Epidemiology, Harvard T.H. Chan School of Public Health, Boston, MA USA; 95 Channing Division of Network Medicine, Department of Medicine, Brigham and Women’s Hospital and Harvard Medical School, Boston, MA USA; 96 Centre for Cancer Genetic Epidemiology, Department of Oncology, University of Cambridge, Cambridge, UK; 97 School of Life Sciences, University of Westminster, London, UK; 98 Department of General Practice and Primary Healthcare, University of Helsinki, Helsinki University Hospital, Helsinki, Finland; 99 Yong Loo Lin School of Medicine, Department of Obstetrics and Gynecology and Human Potential Translational Research Programme, National University Singapore, Singapore City, Singapore; 100 Singapore Institute for Clinical Sciences (SICS), Agency for Science, Technology and Research (A*STAR), Singapore City, Singapore; 101 Department of Biobehavioral Health, Pennsylvania State University, University Park, PA USA; 102 The Breast Cancer Now Toby Robins Research Centre, The Institute of Cancer Research, London, UK; 103 Genomic Medicine Group, International Cancer Genetics and Epidemiology Group Fundación Pública Galega de Medicina Xenómica, Instituto de Investigación Sanitaria de Santiago de Compostela (IDIS), Complejo Hospitalario Universitario de Santiago, SERGAS Santiago de Compostela, Coruña, Spain; 104 Medical Oncology Department, Hospital Clínico San Carlos Instituto de Investigación Sanitaria San Carlos (IdISSC), Centro Investigación Biomédica en Red de Cáncer (CIBERONC), Madrid, Spain; 105 Human Genotyping Unit-CeGen, Spanish National Cancer Research Centre (CNIO), Madrid, Spain; 106 Research Unit of Molecular Epidemiology, Helmholtz Zentrum München—German Research Center for Environmental Health, Neuherberg, Germany; 107 Institute of Epidemiology, Helmholtz Zentrum München—German Research Center for Environmental Health, Neuherberg, Germany; 108 Team ‘Exposome and Heredity’, CESP, Gustave Roussy INSERM, University Paris-Saclay, UVSQ, Orsay, France; 109 Department of Preventive Medicine, Keck School of Medicine, University of Southern California, Los Angeles, CA USA; 110 Department of Oncology, Södersjukhuset, Stockholm, Sweden; 111 Molecular Genetics of Breast Cancer, German Cancer Research Center (DKFZ), Heidelberg, Germany; 112 Department of Pediatrics, University of Pennsylvania Perelman School of Medicine, Philadelphia, PA USA; 113 Division of Pulmonary Medicine, Children’s Hospital of Philadelphia, Philadelphia, PA USA; 114 Division of Obstetrics and Gynaecology, University of Western Australia, Crawley, Western Australia Australia; 115 Department of Obstetrics and Gynaecology, University of Melbourne and The Royal Women’s Hospital, Parkville, Victoria Australia; 116 Department of Medical Oncology, Erasmus MC Cancer Institute, Rotterdam, The Netherlands; 117 Dr. Margarete Fischer-Bosch-Institute of Clinical Pharmacology, Stuttgart, Germany; 118 University of Tübingen, Tübingen, Germany; 119 Centre for Epidemiology and Biostatistics, Melbourne School of Population and Global Health, The University of Melbourne, Melbourne, Victoria Australia; 120 Department of Nutrition, Harvard T.H. Chan School of Public Health School of Public Health, Boston, MA USA; 121 Australian Breast Cancer Tissue Bank, Westmead Institute for Medical Research, University of Sydney, Sydney, New South Wales Australia; 122 Oncology, Department of Clinical Sciences in Lund, Lund University, Lund, Sweden; 123 Department of Epidemiology and Population Health, Stanford University School of Medicine Stanford, Stanford, CA USA; 124 Department of Medicine, Division of Oncology Stanford Cancer Institute, Stanford University School of Medicine Stanford, Stanford, CA USA; 125 Hebrew SeniorLife Institute for Aging Research, Boston, MA USA; 126 Harvard Medical School, Boston, MA USA; 127 Institute of Biochemistry and Genetics of the Ufa Federal Research Centre of the Russian Academy of Sciences, Ufa, Russia; 128 Department of Genetics and Fundamental Medicine, Bashkir State University, Ufa, Russia; 129 Department of Medical Genetics, Oslo University Hospital and University of Oslo, Oslo, Norway; 130 Institute of Clinical Medicine, Faculty of Medicine, University of Oslo, Oslo, Norway; 131 Department of Computational and Quantitative Medicine, City of Hope, Duarte, CA USA; 132 City of Hope Comprehensive Cancer Center, City of Hope, Duarte, CA USA; 133 Laboratory for Translational Genetics, Department of Human Genetics, KU Leuven, Leuven, Belgium; 134 VIB Center for Cancer Biology, VIB, Leuven, Belgium; 135 Laboratory of Epidemiology and Population Sciences, National Institute on Aging, Intramural Research Program, National Institutes of Health, Bethesda, MD USA; 136 School of Biomedical Sciences, Queensland University of Technology, Brisbane, Queensland Australia; 137 Department of Molecular Medicine and Surgery, Karolinska Institutet, Stockholm, Sweden; 138 Department of Clinical Genetics, Karolinska University Hospital, Stockholm, Sweden; 139 Translational Cancer Research Area, University of Eastern Finland, Kuopio, Finland; 140 Institute of Clinical Medicine, Pathology and Forensic Medicine, University of Eastern Finland, Kuopio, Finland; 141 Wellcome Trust Centre for Human Genetics, University of Oxford, Oxford, UK; 142 Oxford Centre for Diabetes, Endocrinology and Metabolism, University of Oxford, Churchill Hospital, Oxford, UK; 143 NIHR Oxford Biomedical Research Centre, Churchill Hospital, Oxford, UK; 144 Institute of Human Genetics, Klinikum rechts der Isar, Technical University of Munich, School of Medicine, Munich, Germany; 145 Biostatistics Unit, The Cyprus Institute of Neurology and Genetics, Nicosia, Cyprus; 146 Cancer Epidemiology Division, Cancer Council Victoria, Melbourne, Victoria Australia; 147 Institute for Molecular Bioscience, The University of Queensland, Brisbane, Queensland Australia; 148 Department of Obstetrics and Gynecology, Helsinki University Hospital, University of Helsinki, Helsinki, Finland; 149 School of Biomedical Sciences, Faculty of Health, Centre for Genomics and Personalised Health, Queensland University of Technology, Brisbane, Queensland Australia; 150 Institute for Occupational Medicine and Maritime Medicine, University Medical Center Hamburg-Eppendorf, Hamburg, Germany; 151 Institute of Medical Biometry and Epidemiology, University Medical Center Hamburg-Eppendorf, Hamburg, Germany; 152 Epidemiology Branch, National Institute of Environmental Health Sciences, NIH Research Triangle Park, Durham, NC USA; 153 Clinical Genetics Research Lab, Department of Cancer Biology and Genetics, Memorial Sloan Kettering Cancer Center, New York City, NY USA; 154 Clinical Genetics Service, Department of Medicine, Memorial Sloan Kettering Cancer Center, New York City, NY USA; 155 Interdisciplinary Center Psychopathology and Emotion Regulation, University Medical Center Groningen, University of Groningen, Groningen, The Netherlands; 156 Department of Clinical Immunology, Rigshospitalet—University of Copenhagen, Copenhagen, Denmark; 157 Department of Clinical Medicine, Faculty of Health and Medical Sciences, University of Copenhagen, Copenhagen, Denmark; 158 Psychiatric and Neurodevelopmental Genetics Unit, Massachusetts General Hospital and Harvard Medical School, Boston, MA USA; 159 Medical and Population Genetics Program, Broad Institute of MIT and Harvard, Cambridge, MA USA; 160 Stanley Center for Psychiatric Research, Broad Institute of MIT and Harvard, Cambridge, MA USA; 161 Wellcome Trust Sanger Institute, Wellcome Trust Genome Campus, Hinxton, UK; 162 Analytic and Translational Genetics Unit, Massachusetts General Hospital and Harvard Medical School, Boston, MA USA; 163 Institute for Molecular Medicine Finland (FIMM), University of Helsinki, Helsinki, Finland; 164 Department of Clinical Immunology, Zealand University Hospital, Køge, Denmark; 165 Institute for Medical Information Processing, Biometry and Epidemiology—IBE, Ludwig-Maximilians-Universität München, Munich, Germany; 166 Research Centre for Genetic Engineering and Biotechnology ‘Georgi D. Efremov’, MASA, Skopje, Republic of North Macedonia; 167 National Institute for Health and Welfare, Helsinki, Finland; 168 Unit of Preventive Medicine: Molecular Bases of Genetic Risk, Department of Experimental Oncology, Fondazione IRCCS, Istituto Nazionale dei Tumori (INT), Milan, Italy; 169 Faculty of Medicine, Clalit National Cancer Control Center, Carmel Medical Center and Technion, Haifa, Israel; 170 Department of Clinical Epidemiology, Leiden University Medical Center, Leiden, The Netherlands; 171 IRCCS Neuromed, Isernia, Italy; 172 Division of Oncology, Faculty of Medicine, School of Health Sciences, University of Thessaly, Larissa, Greece; 173 Institute for Community Medicine, University Medicine Greifswald, Greifswald, Germany; 174 Division of Molecular Pathology, The Netherlands Cancer Institute, Amsterdam, The Netherlands; 175 Division of Psychosocial Research and Epidemiology, The Netherlands Cancer Institute—Antoni van Leeuwenhoek Hospital, Amsterdam, The Netherlands; 176 Genetic Epidemiology Group, School of Population and Global Health, University of Western Australia Perth, Perth, Western Australia Australia; 177 Department of Population Health Sciences, Weill Cornell Medicine, New York City, NY USA; 178 Department of Population Science, American Cancer Society, Atlanta, GA USA; 179 Public Health Genomics Unit, Department of Chronic Disease Prevention, National Institute for Health and Welfare, Helsinki, Finland; 180 Program in Genetic Epidemiology and Statistical Genetics, Harvard T.H. Chan School of Public Health, Boston, MA USA; 181 Department of Quantitative Health Sciences, Division of Epidemiology, Mayo Clinic Rochester, Rochester, MN USA; 182 Laboratory of Cancer Genetics and Tumor Biology, Translational Medicine Research Unit, Biocenter Oulu, University of Oulu, Oulu, Finland; 183 Laboratory of Cancer Genetics and Tumor Biology, Northern Finland Laboratory Centre, Oulu, Finland; 184 Institute of Environmental Medicine, Karolinska Institutet, Stockholm, Sweden; 185 Division of Gastroenterology, Hepatology and Nutrition, Children’s Hospital of Philadelphia, Philadelphia, PA USA; 186 Division of Epidemiology, Department of Medicine, Vanderbilt Epidemiology Center, Vanderbilt-Ingram Cancer Center, Vanderbilt University School of Medicine, Nashville, TN USA; 187 Department of Internal Medicine, Division of Endocrinology, Leiden University Medical Center, Leiden, The Netherlands; 188 Geriatric Unit, Local Health Toscana Centro, Florence, Italy; 189 Human Genetics Center, University of Texas Health Science Center at Houston, Houston, TX USA; 190 Amsterdam Reproduction and Development Research Institute, Amsterdam, The Netherlands; 191 German Center for Diabetes Research (DZD), Neuherberg, Germany; 192 Division of Endocrinology and Diabetes, Children’s Hospital of Philadelphia, Philadelphia, PA USA; 193 Institute of Clinical Research, University of Southern Denmark, Department of Obstetrics and Gynecology, Odense University Hospital, Odense, Denmark; 194 Department of Maternity and Gynaecology, John Hunter Hospital, Newcastle, New South Wales Australia; 195 Department of Obstetrics and Gynaecology, University of Cambridge, Cambridge, UK; 196 State Institute of Health, Bavarian Health and Food Safety Authority (LGL), Oberschleissheim, Germany; 197 Centre for Medical Informatics, Usher Institute, University of Edinburgh, Edinburgh, UK; 198 Department of Epidemiology and Biostatistics, School of Public Health, Peking University, Beijing, China; 199 Center for Public Health and Epidemic Preparedness and Response, Peking University, Beijing, China; 200 Children and Adolescent Clinic, Haukeland University Hospital, Bergen, Norway; 201 Boston University Chobanian and Avedisian School of Medicine, Department of Medicine, Section of General Internal Medicine, Boston, MA USA; 202 Genetics of Complex Traits, University of Exeter Medical School, University of Exeter, RILD Level 3, Royal Devon and Exeter Hospital, Exeter, UK; 203 Centre Hospitalier Universitaire (CHU) Sainte-Justine Research Center, University of Montreal, Montreal, Quebec Canada; 204 Department of Pediatrics, University of Montreal, Montreal, Quebec Canada; 205 Department of Biochemistry and Molecular Medicine, University of Montreal, Montreal, Quebec Canada; 206 International Center for Research and Research Training in Endocrine Disruption of Male Reproduction and Child Health (EDMaRC), Copenhagen University Hospital—Rigshospitalet, Copenhagen, Denmark; 207 Department of Clinical Medicine, University of Copenhagen, Copenhagen, Denmark; 208 Department of Clinical Immunology, Aarhus University Hospital, Aarhus, Denmark; 209 Department of Clinical Medicine, Aarhus University, Aarhus, Denmark; 210 Laboratory for Genomics of Diabetes and Metabolism, RIKEN Center for Integrative Medical Sciences, Yokohama, Japan; 211 Metabolic Research Laboratory, Wellcome-MRC Institute of Metabolic Science, University of Cambridge School of Clinical Medicine, Cambridge, UK; 212 Department of Paediatrics, University of Cambridge, Cambridge, UK

**Keywords:** Genome-wide association studies, Obesity

Correction to: *Nature Genetics* 10.1038/s41588-024-01798-4, published online 1 July 2024.

In the version of the article initially published, in Fig. 5c, the trajectory labels now reading T01, T02, T03, T04, T05, T06, T07, T08, T09 and T10 appeared incorrectly as T-01, T-02, T-03, T-04, T-05, T-06, T-07, T-08, T-09 and T-10. The figure has now been amended in the HTML and PDF versions of the article.

