# Supplementary material for: Understanding the genetic complexity of puberty timing across the allele frequency spectrum
Source: Nat Genet. 2024 Jul 1;56(7):1397–411. doi: 10.1038/s41588-024-01798-4 (PMC11250262; doi:10.1038/s41588-024-01798-4)
Supplement: Supplementary file 1 — Supplementary Figs. 1–16, Consortia membership, Study-specific text and acknowledgements, Additional acknowledgements and Additional funding information. [file 41588_2024_1798_MOESM1_ESM.pdf]

nature genetics

Supplementary information https://doi.org/10.1038/s41588-024-01798-4

Understanding the genetic complexity of puberty timing across the allele frequency spectrum

In the format provided by the authors and unedited

Supplementary information

Table of contents

Supplementary Figures....2
Consortia membership....17
The China Kadoorie Biobank Collaborative Group....17
Lifelines Cohort Study....18
Danish Blood Donor Study....18
The Ovarian Cancer Association Consortium....19
The Breast Cancer Association Consortium....32
Study-specific text & acknowledgements....39
Additional acknowledgements....50
Additional funding information....51

Supplementary Figures

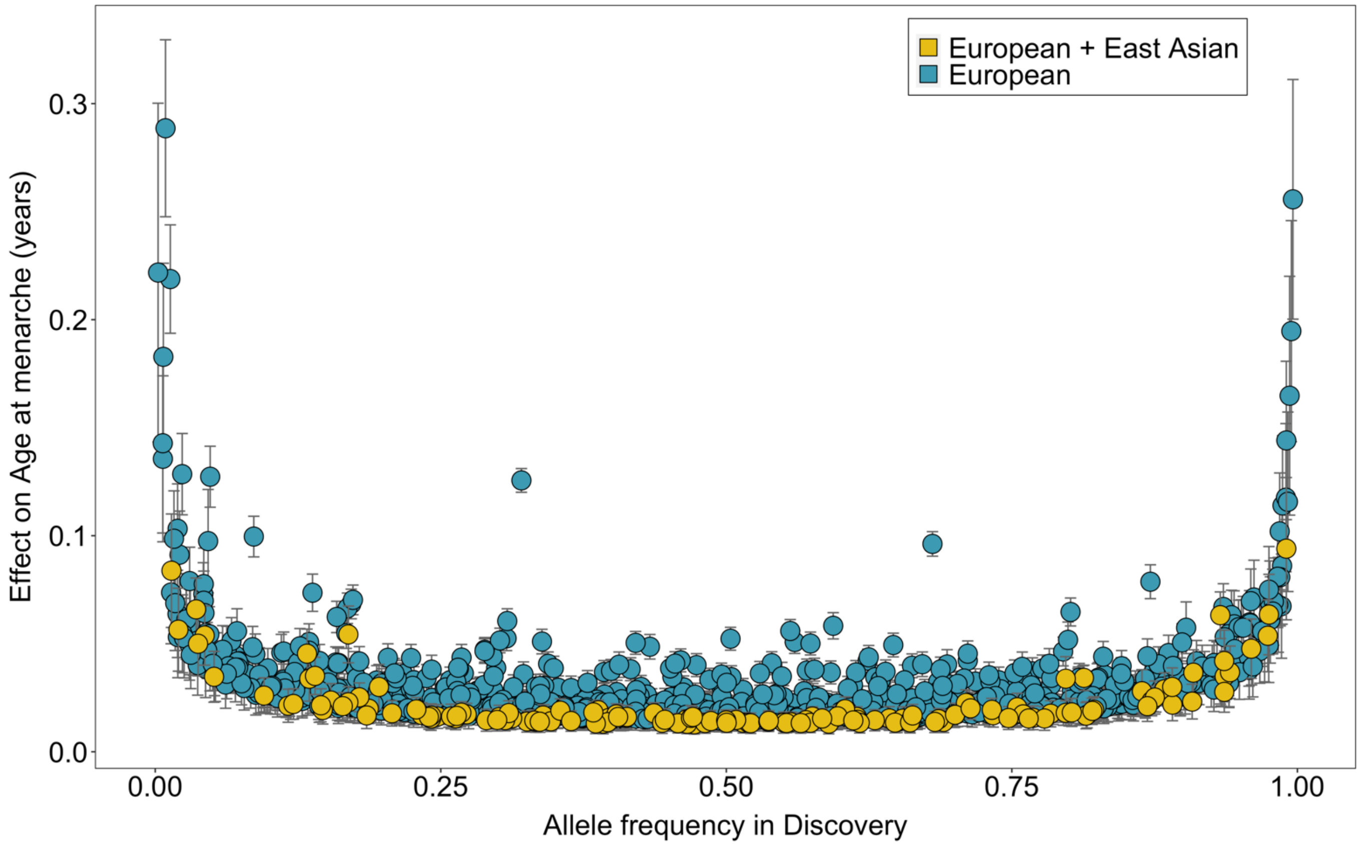

Supplementary Figure 1 | Distribution of effect sizes across 1080 independent signals with age at menarche (AAM). For each signal the effect size estimate and 95% confidence intervals are shown from the corresponding Discovery analysis, i.e. from the European-only or the ancestry combined meta-analysis, as indicated by point colours. Estimates are aligned towards the menarche-increasing alleles. Extended data are shown in Supplementary Table 2.

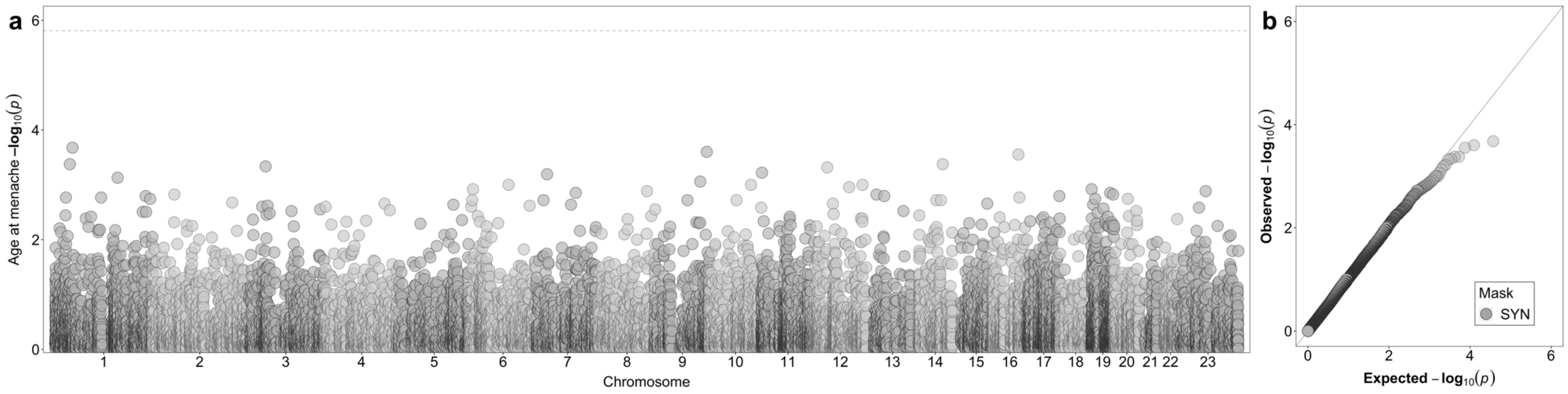

Supplementary Figure 2 | Synonymous variant associations with AAM. (a) Manhattan plot showing the gene burden associations from BOLT-LMM with age at menarche for the synonymous variant mask, as a negative control analysis. The horizontal line indicates the exome-wide significance threshold ($P<1.54x10^{-6}$). (b) Quantile-quantile (QQ) plot of the gene burden associations from BOLT-LMM for the synonymous variant mask.

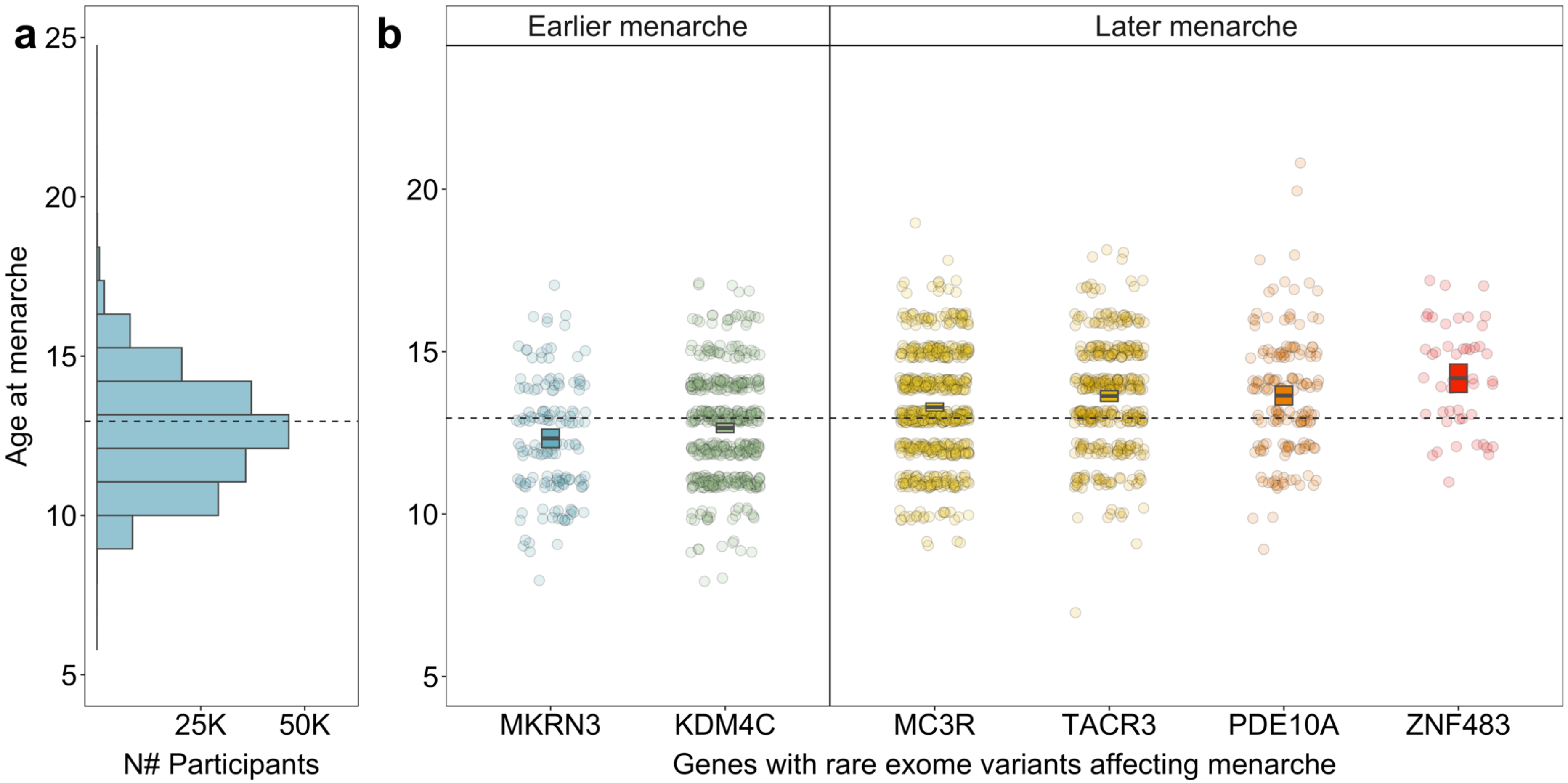

Supplementary Figure 3 | Distribution of age at menarche (AAM) in UK Biobank. Reported AAM in: (a) All white European unrelated female participants (N=187,941) (b) Carriers of qualifying rare variants in the associated genes. The horizontal dotted line indicates the mean AAM among non-carriers (N=185,929). Mean and 95% confidence intervals (CIs) for each carrier group are indicated by horizontal bars and boxes.

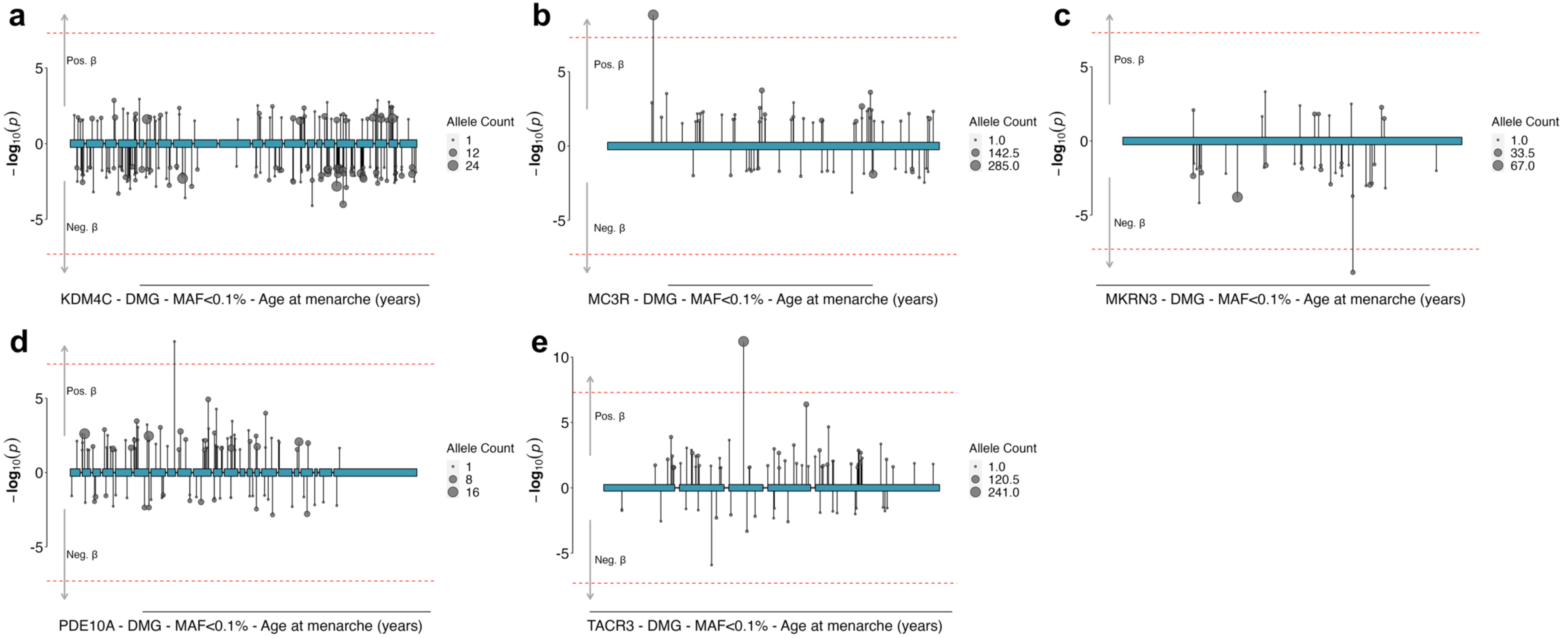

Supplementary Figure 4 | Variant-level associations with AAM in identified genes in UK Biobank. Rare exome variant associations from BOLT-LMM with AAM for variants in *KDM4C* (a), *MC3R* (b), *MKRN3* (c), *PDE10A* (d) and *TACR3* (e). The equivalent plot for *ZNF483* can be found in Fig. 3. Variant collapsing masks included variants with a minor allele frequency (MAF) < 0.1% and annotated as either high-confidence protein truncating variants (HC_PTV) or HC_PTV plus missense variants with a high CADD score (>=25, denoted DMG). Each variant association is represented by a circle and vertical line: the line length indicates the P-value (-log10), in the direction of its effect on AAM in carriers of the rare allele, and the circle size indicates the number of carriers of each variant (i.e. allele count). Exons are indicated by the blue boxes.

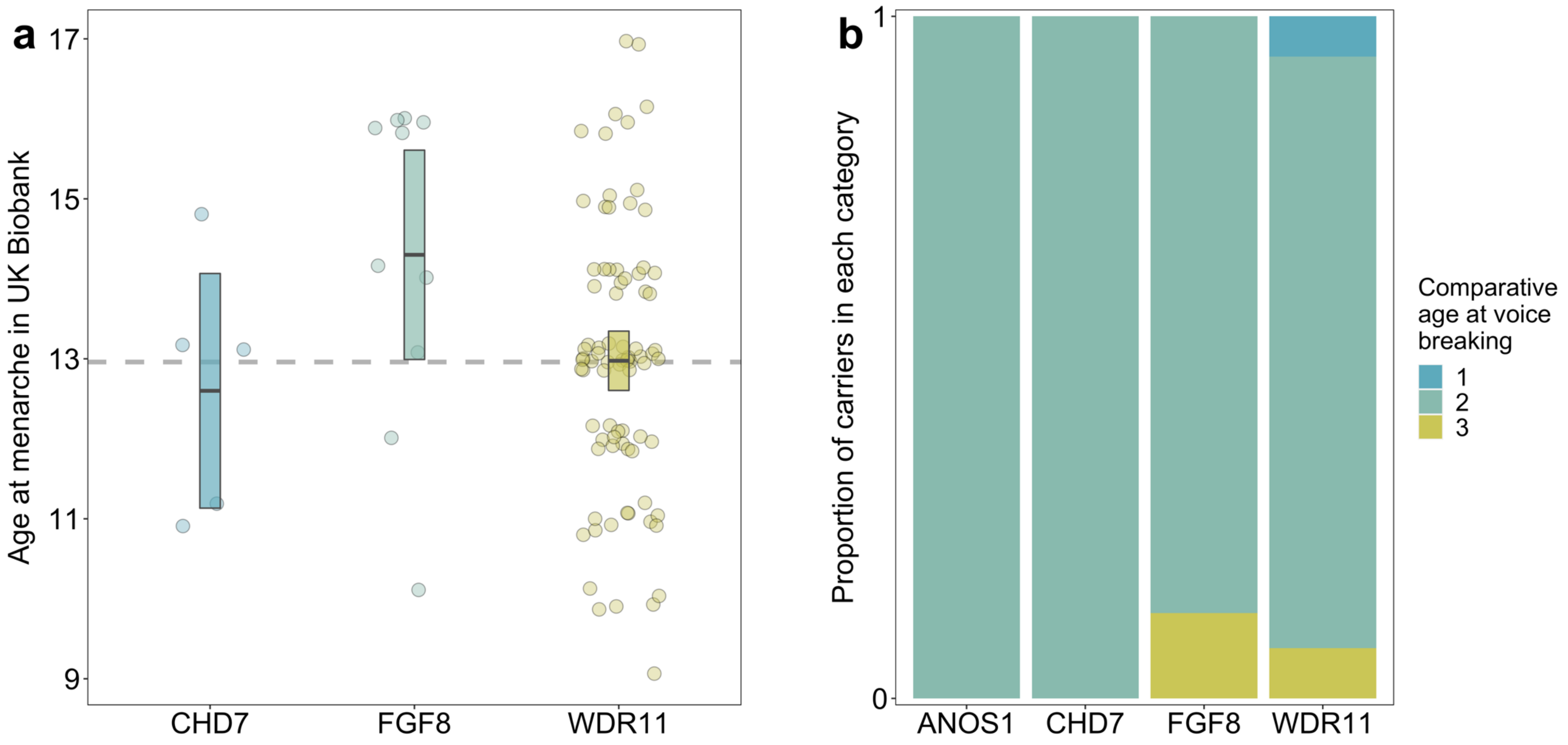

Supplementary Figure 5 | Puberty onset in carriers of IHH panel genes in UK Biobank. (a) Distribution of age at menarche (AAM) in UK Biobank white European unrelated female participants carrying qualifying rare variants in IHH panel genes. The horizontal dotted line indicates mean AAM among non-carriers (N=185,929). Mean and 95% confidence intervals (CIs) for each carrier group are indicated by horizontal bars and boxes. (b) Comparative age at voice breaking in men carrying qualifying rare variants in IHH panel genes. 1 indicates self-reported “younger than average age” at voice breaking, 2 “average age” and 3 “later than average age”.

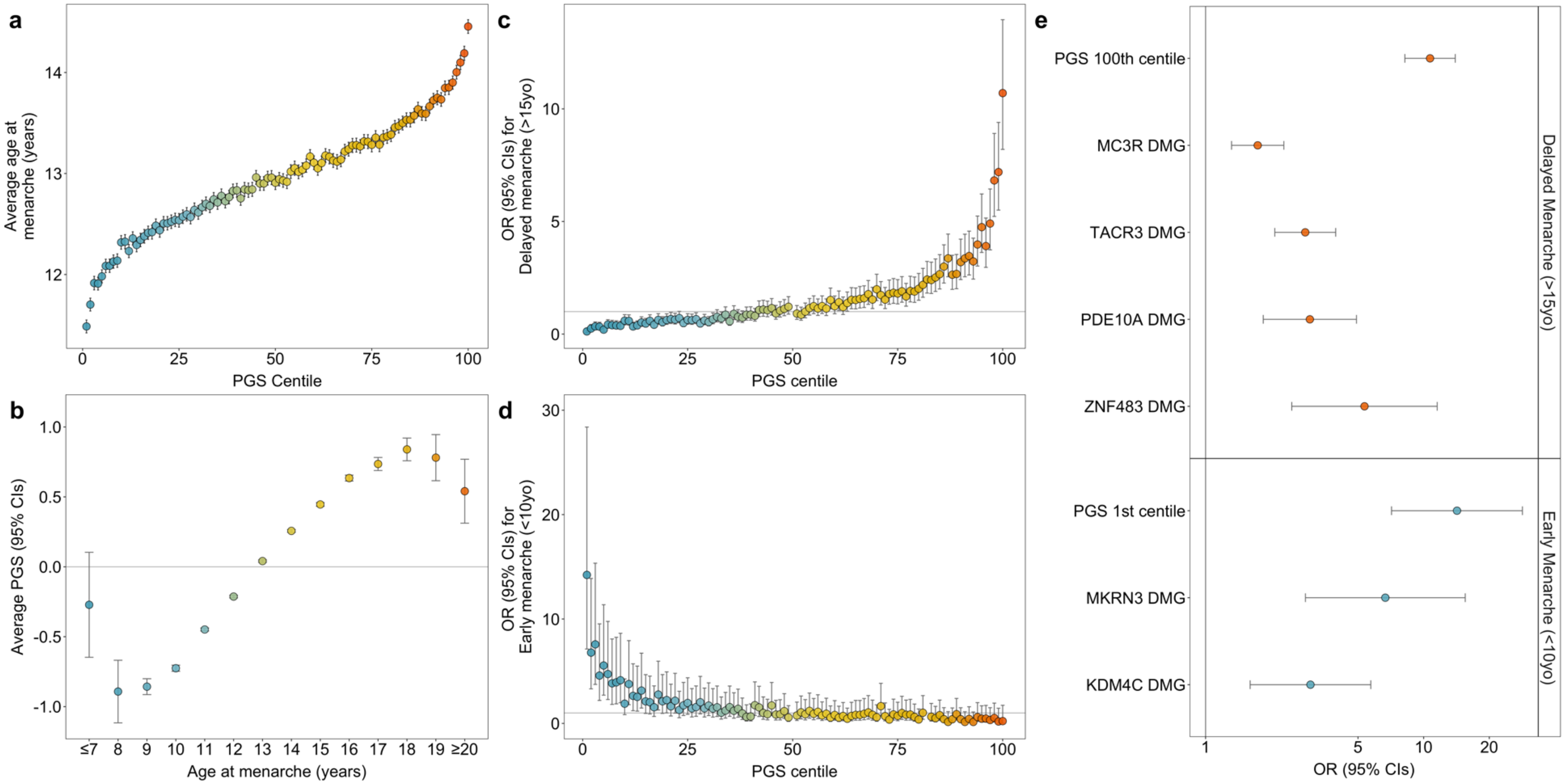

Supplementary Figure 6 | Polygenic score (PGS) associations with age at menarche (AAM). The PGS for AAM was derived using summary statistics from the European-only MA and excluding UK Biobank, and then applied to white European, unrelated female participants in UK Biobank (N=187,941). (a) Means with 95% confidence intervals (CIs) of AAM by PGS centile (N=1,880 per centile). (b) Means with 95% CIs of standardised PGS by AAM. (c-d) Associations of each PGS centile compared to the 50th PGS centile (OR with 95% CIs) with (c) delayed menarche (menarche after 15 years) and (d) early menarche (menarche before 10 years). Controls were women who reported menarche at 12 or 13 years. PGS centiles with fewer than 2 participants were omitted. (e) Gene burden associations between qualifying variants in the exome-identified genes compared to extreme PGS centiles, with delayed or early menarche defined as above, presented as ORs with 95% CIs. Extended data are shown in Supplementary Tables 8-10.

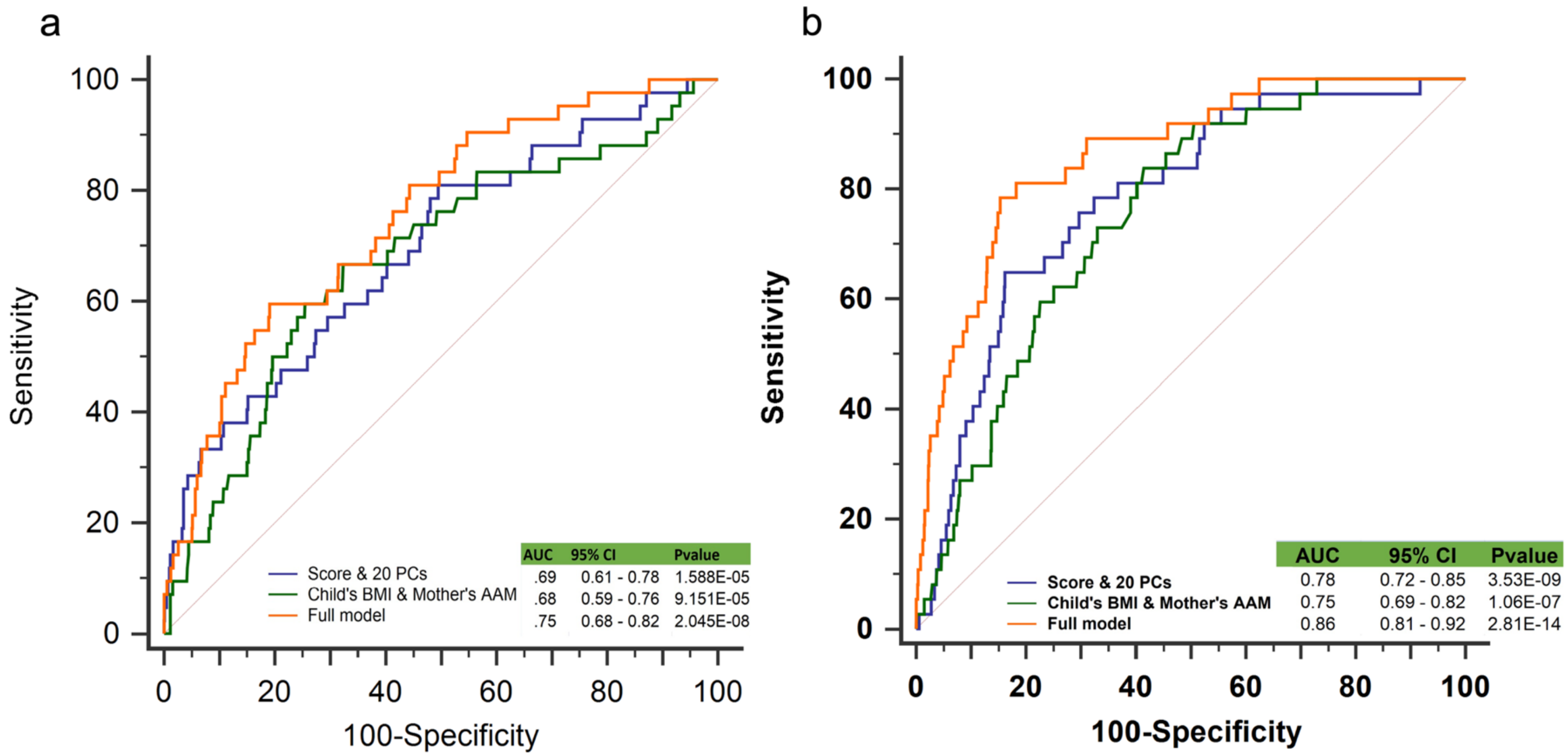

Supplementary Figure 7 | Receiver operating characteristic (ROC) curve for predicting extremes of age at menarche (AAM) in the ALSPAC study. Predictive performance of linear regressions adjusting for the genetic, clinical, and combined predictor and presented as ROC graphs against (a) early AAM and (b) delayed AAM in the ALSPAC study (N= 3,140). Extended data are shown in Supplementary Table 11.

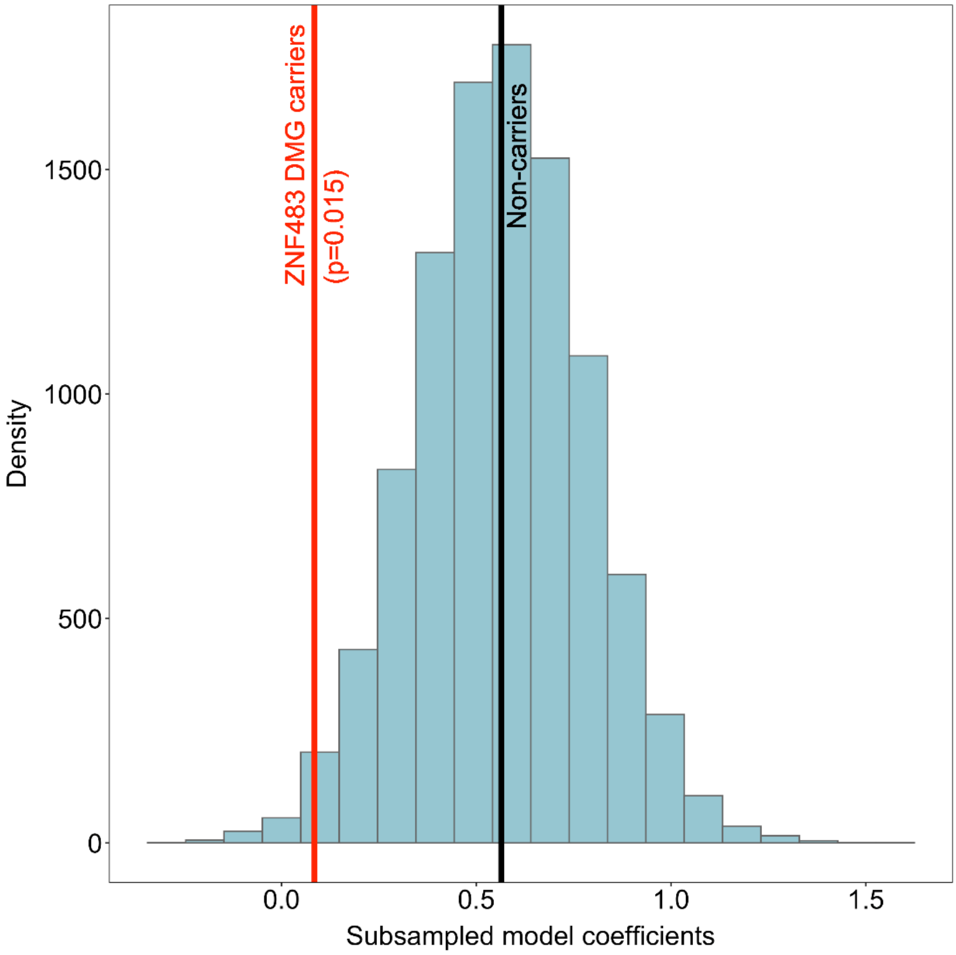

Supplementary Figure 8 | Epistatic interaction between the polygenic score (PGS) and carriage of rare damaging variants in *ZNF483* on age at menarche (AAM). Blue columns indicate the beta coefficients for the PGS-AAM association among 10,000 random subsamples of 49 white European unrelated female non-carriers in UK Biobank, tested in a linear model. The beta coefficient from the full sample of non-carriers is indicated by the black vertical line (0.564 years per SD, SE 0.003, $P<2x10^{-16}$) and the beta coefficient seen in *ZNF483* variant carriers is indicated by the red vertical line (difference in coefficients: -0.480, SE 0.214, P=0.025). The probability of observing a beta coefficient smaller than that in *ZNF483* variant carriers was estimated as the proportion of subsampled coefficients that were smaller than 0.084 (i.e., 0.564-0.480).

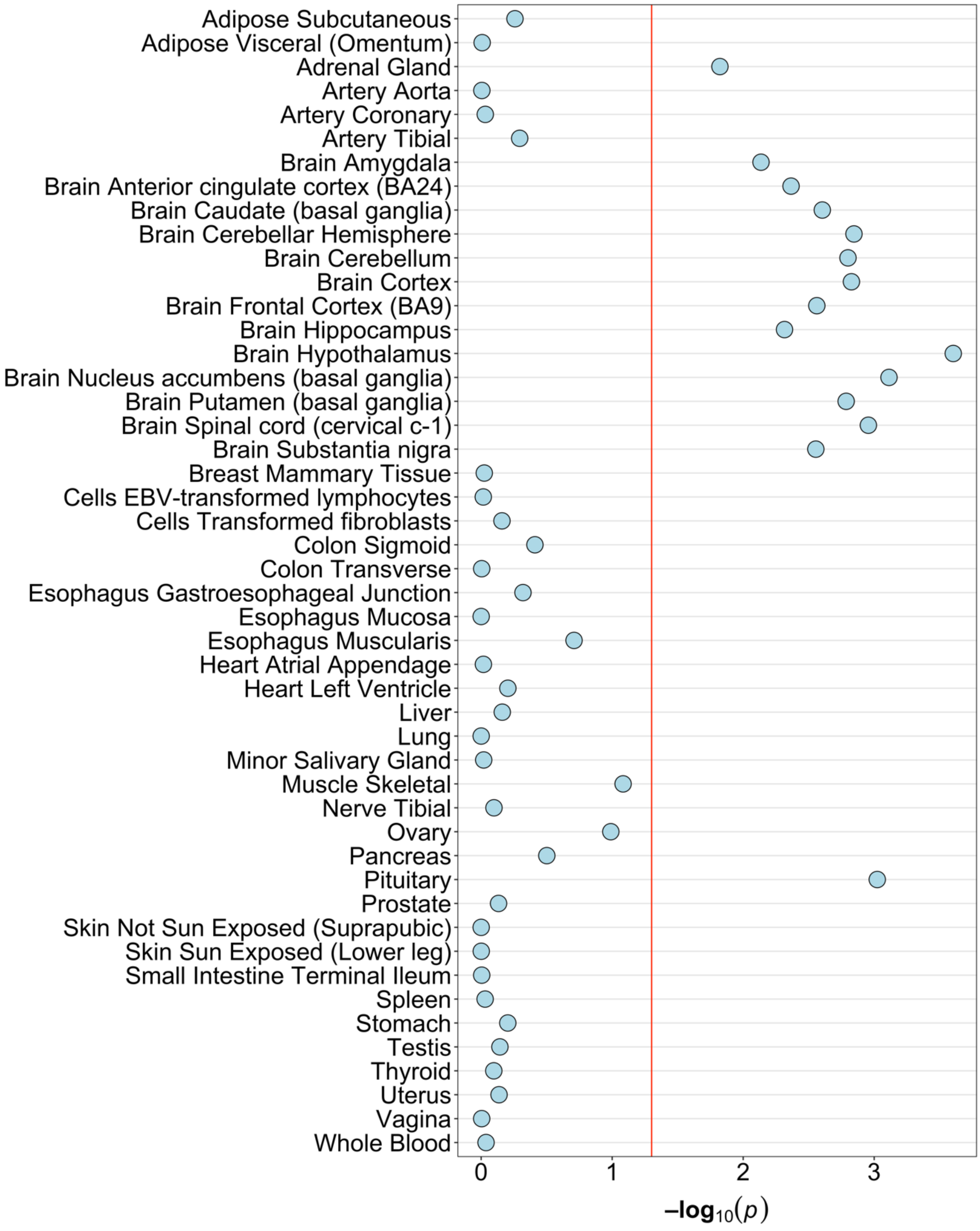

Supplementary Figure 9 | Tissue enrichment for age at menarche (AAM) GWAS associations. Linkage-disequilibrium score regression to specifically expressed genes (LDSC-SEG) was used to test for enrichment for AAM GWAS associations among European-only samples among genes specifically expressed across the different GTEx tissues. LDSC-SEG unadjusted P-values represent a one-sided test that the coefficient is greater than zero. Extended data are shown in Supplementary Table 17.

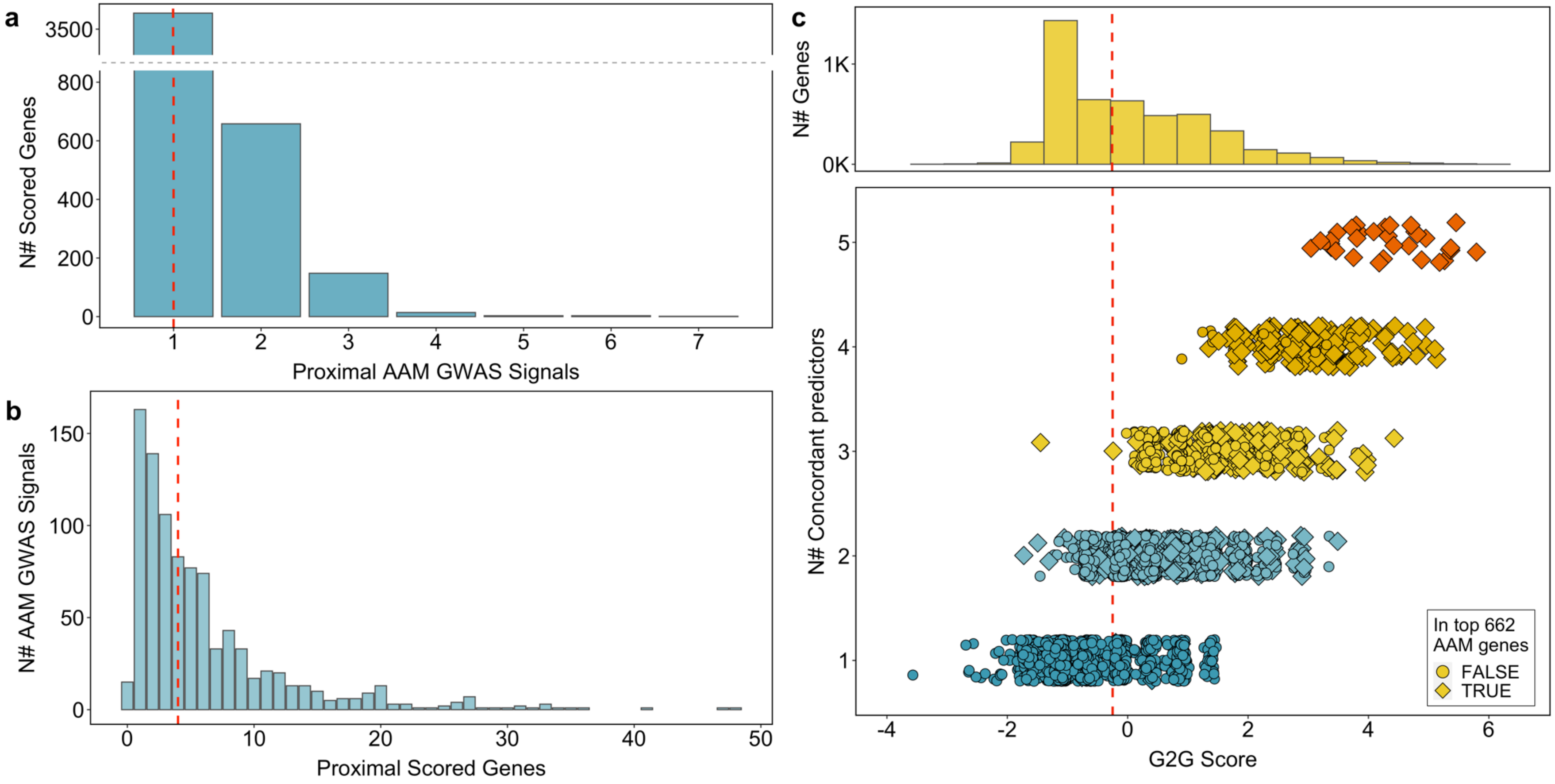

Supplementary Figure 10 | Distribution of age at menarche (AAM) GWAS signals and genes, as identified by GWAS to Genes (G2G). (a) Based on the European-only GWAS meta-analysis, 4,668 genes were implicated by G2G as potential regulators of AAM. Most were proximal to (within 500kb) only 1 GWAS signal (median signals per gene: 1; range: 1 to 7). (b) Conversely, each GWAS signal was annotated to be proximal (within 500kb) to (median) 4 genes (range: 0 to 48). (c) The 4,657 genes had a median G2G score -0.25 (range -3.57 to 5.88) derived from a maximum of 6 predictors (up to 5 observed). The 665 high confidence AAM genes were defined as the top-scoring gene at each independent AAM signal with at least 2 concordant predictors. Extended data are shown in Supplementary Table 13.

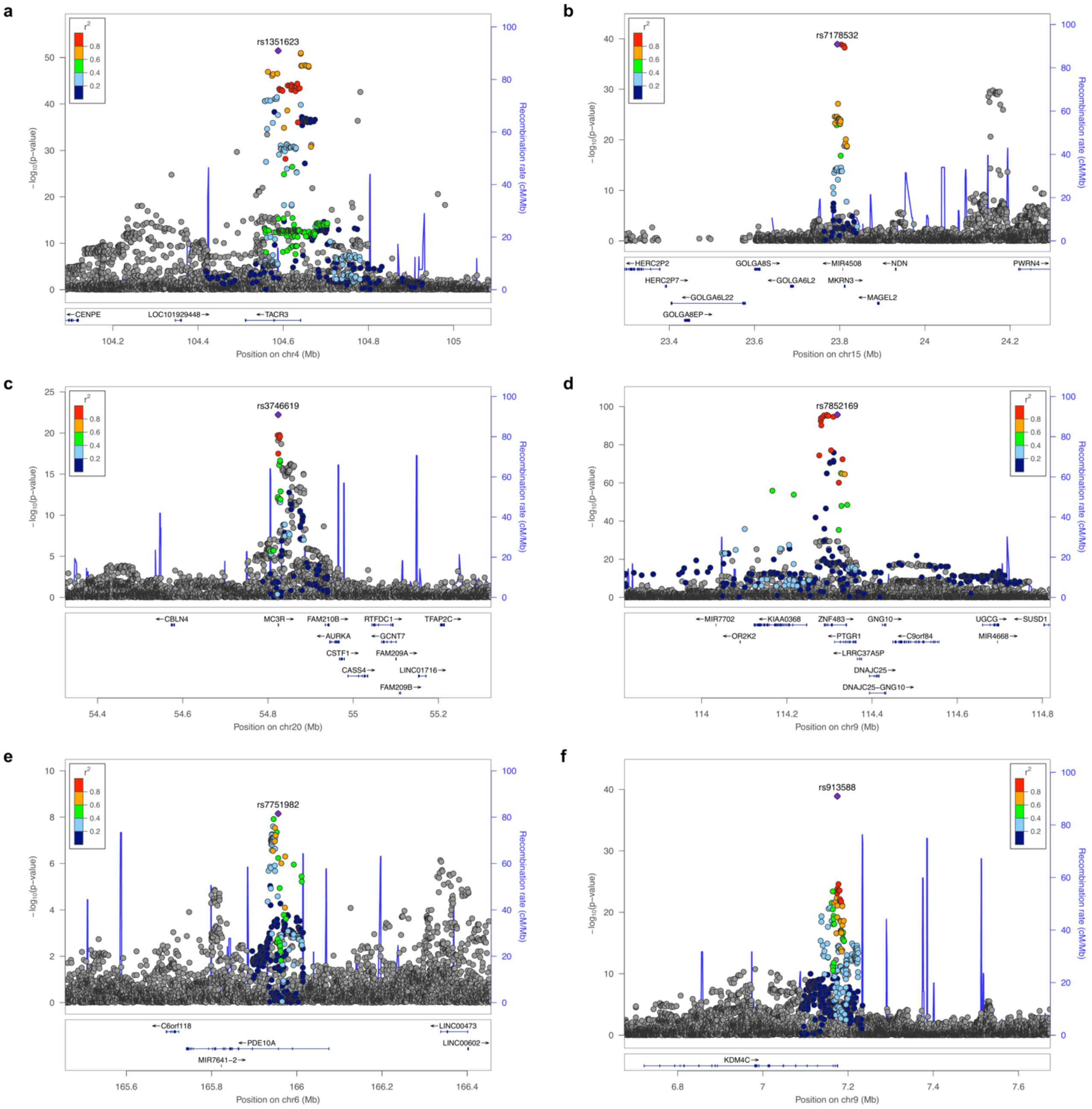

Supplementary Figure 11 | GWAS loci proximal to the six exome-wide significantly associated genes. Associations among European-only samples in regions surrounding the 6 genes (±500kb) identified via exome-wide rare variant associations with AAM; (a) *TACR3*, (b) *MKRN3*, (c) *MC3R*, (d) *ZNF483*, (e) *PDE10A*, and (f) *KDM4C*.

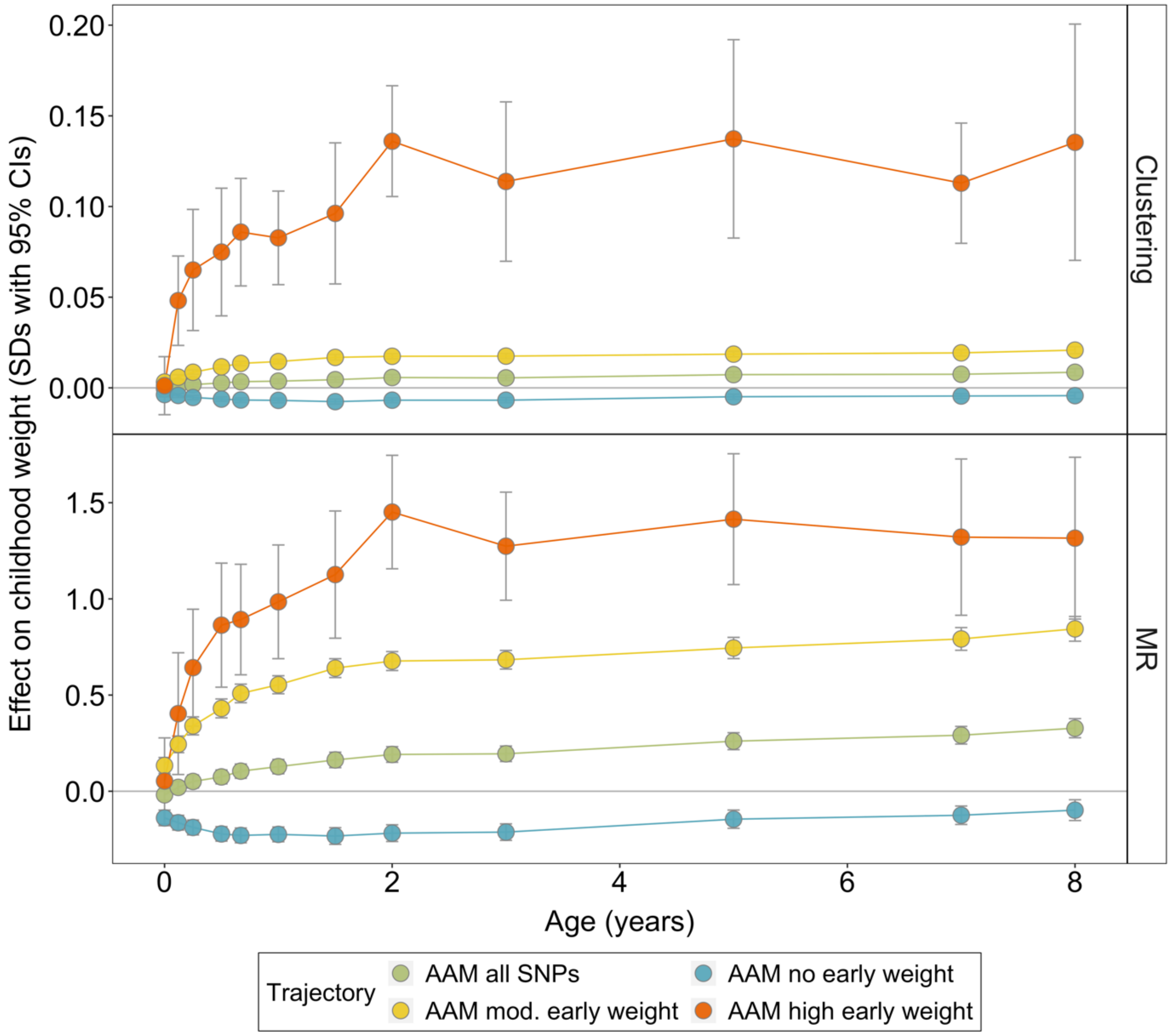

Supplementary Figure 12 | Mean childhood body weight trajectory for each of the three age at menarche (AAM) GWAS SNP clusters (aligned to AAM-decreasing alleles). Mendelian randomisation (MR) estimates with 95% confidence intervals (CIs) for all 1080 AAM signals, and the three AAM SNP clusters as separate exposures, on childhood body weight at 12 time-points (n=26,681 children). Extended data are shown in Supplementary Tables 21 and 22.

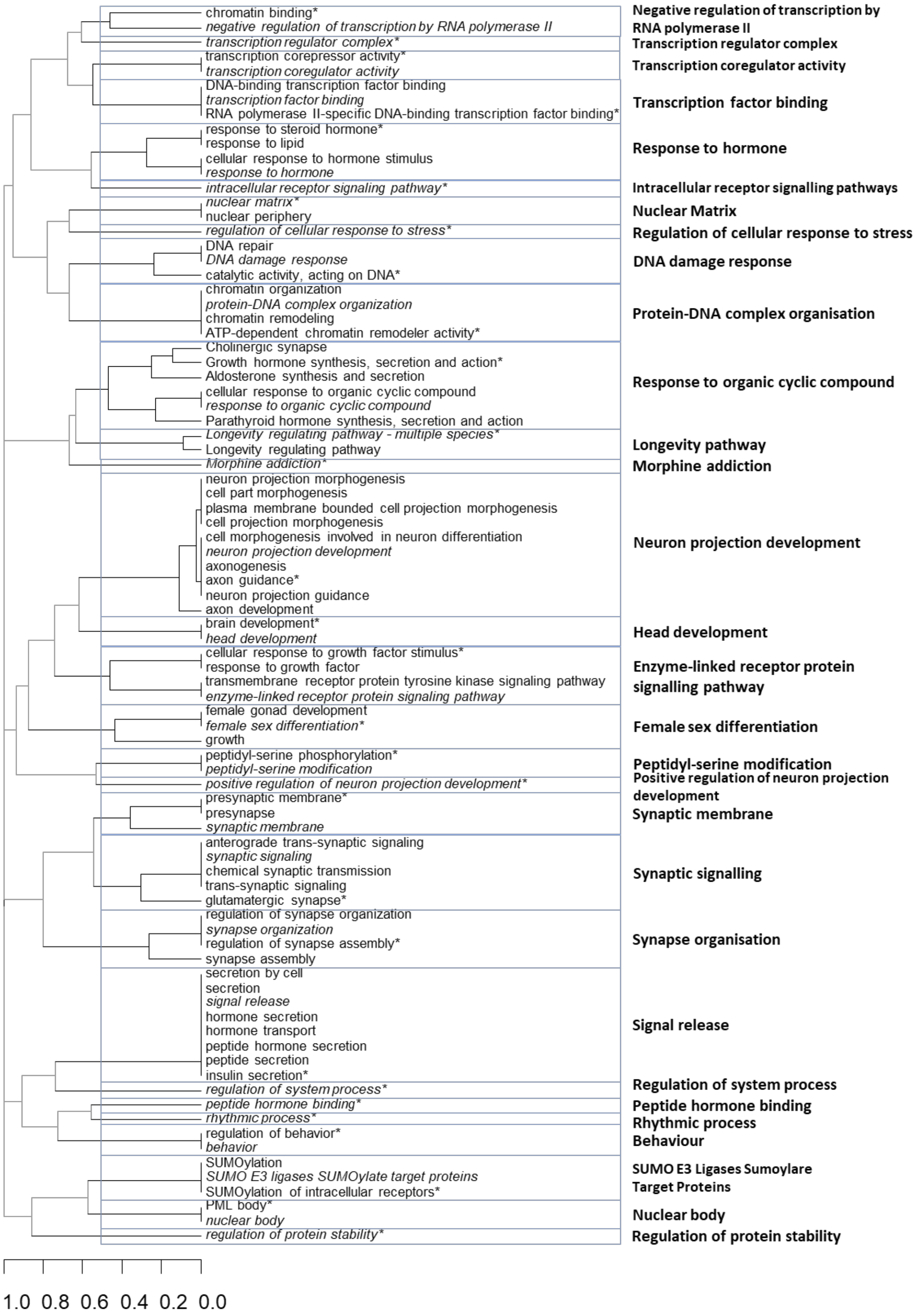

Supplementary Figure 13 | Dendrogram showing clustering of biological pathways enriched for the 665 high confidence AAM genes. In each cluster, the most significantly associated pathway is highlighted in italics and the most highly enriched pathway is marked by an asterisk. Extended data are shown in Supplementary Tables 23-24.

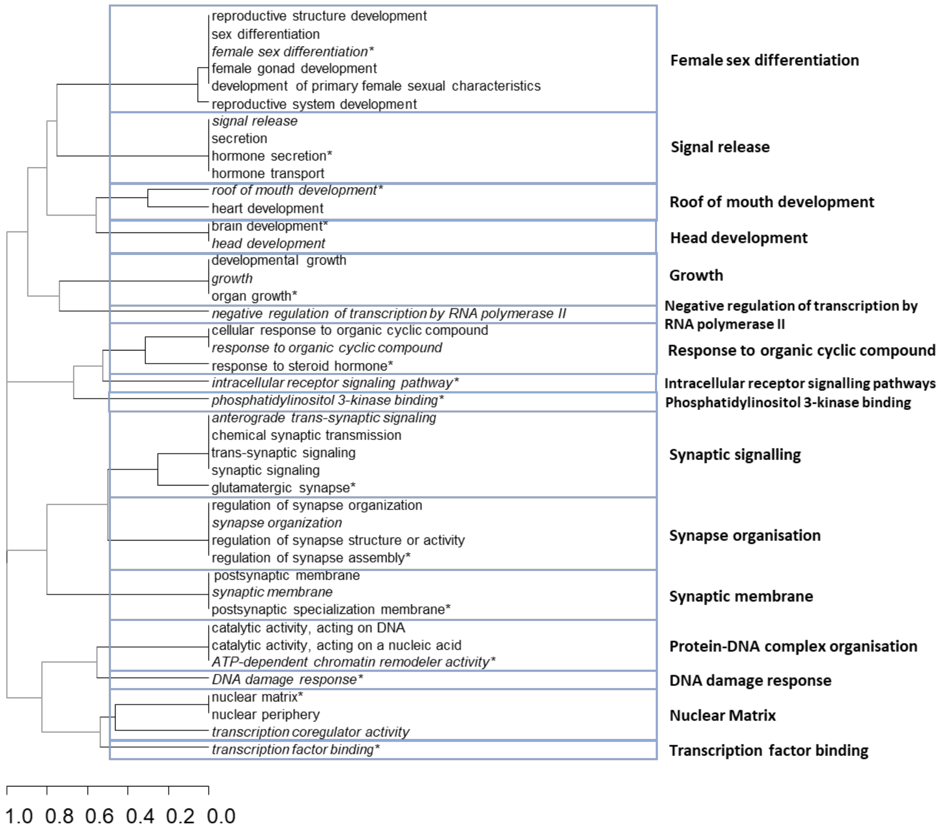

Supplementary Figure 14 | Clustering of biological pathways enriched for AAM genes in the "early weight gain" trajectory. In each cluster the most significant pathway is highlighted in italics and the most highly enriched pathway is marked with an asterisk. Extended data are shown in Supplementary Tables 25-26.

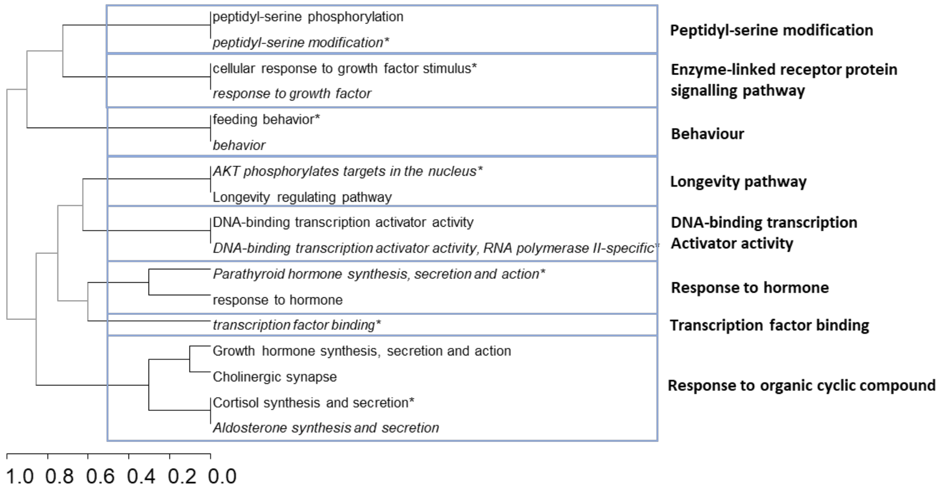

Supplementary Figure 15 | Clustering of biological pathways enriched for AAM genes in the "no early weight gain" trajectory. In each cluster the most significant pathway is highlighted in italics and the most highly enriched pathway is marked with an asterisk. Extended data are shown in Supplementary Tables 25-26.

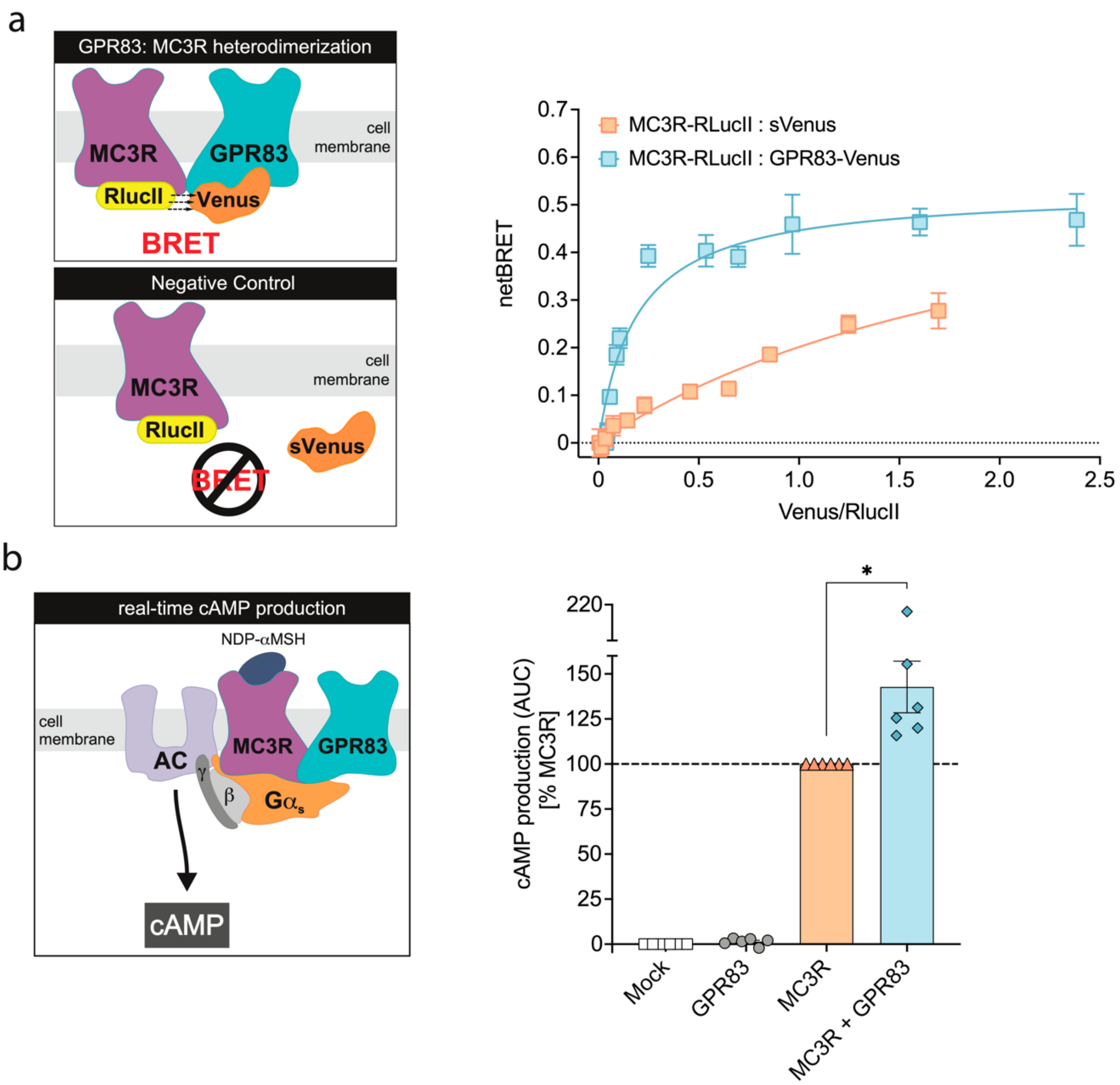

Supplementary Figure 16 | GPR83-MC3R heterodimerization modulates canonical MC3R-cAMP signalling pathway. (a) BRET saturation curve from HEK293 cells co-transfected with a constant amount of MC3R-RlucII donor construct and increasing amounts of the GPR83-Venus acceptor construct, indicating a specific and saturable GPR83-MC3R interaction. The selectivity of the observed signal was further supported by the observation that co-expression of MC3R-RlucII with a soluble acceptor (sVenus) led to lower BRET signals that progressed linearly over the same range of acceptor/donor ratios. (b) NDP-αMSH-stimulated cAMP production area under the curve calculation from HEK293 cells transfected with *MC3R* or co-transfected with both *MC3R* and *GPR83*. The time-resolved data are shown in Figure 6. Data are expressed as a percentage of maximal control response [% MC3R] and plotted as mean ± standard error from 6 independent experiments. Statistical significance was determined by unpaired t-test with Welch's correction; * $P < 0.05$. Extended data are shown in Supplementary Tables 29-31.

Consortia membership

The China Kadoorie Biobank Collaborative Group

International Steering Committee: Junshi Chen, Zhengming Chen (PI), Robert Clarke, Rory Collins, Yu Guo, Liming Li (PI), Chen Wang, Jun Lv, Richard Peto, Robin Walters.

International Co-ordinating Centre, Oxford: Daniel Avery, Derrick Bennett, Ruth Boxall, Sushila Burgess, Ka Hung Chan, Yumei Chang, Yiping Chen, Zhengming Chen, Johnathan Clarke; Robert Clarke, Huaidong Du, Ahmed Edris, Hannah Fry, Simon Gilbert, Mike Hill, Michael Holmes, Pek Kei Im, Andri Iona, Maria Kakkoura, Christiana Kartsonaki, Hubert Lam, Kuang Lin, Mohsen Mazidi, Iona Millwood, Sam Morris, Qunhua Nie, Alfred Pozarickij, Paul Ryder, Saredo Said, Dan Schmidt, Paul Sherliker, Becky Stevens, Iain Turnbull, Robin Walters, Lin Wang, Neil Wright, Ling Yang, Xiaoming Yang, Pang Yao.

National Co-ordinating Centre, Beijing: Yu Guo, Xiao Han, Can Hou, Qingmei Xia, Chao Liu, Jun Lv, Pei Pei, Canqing Yu.

Regional Co-ordinating Centres:

Gansu: Gansu Provincial CDC – Caixia Dong, Pengfei Ge, Xiaolan Ren. Maiji CDC – Zhongxiao Li, Enke Mao, Tao Wang, Hui Zhang, Xi Zhang.

Haikou: Hainan Provincial CDC – Jinyan Chen, Ximin Hu, Xiaohuan Wang. Meilan CDC – Zhendong Guo, Huimei Li, Yilei Li, Min Weng, Shukuan Wu.

Harbin: Heilongjiang Provincial CDC – Shichun Yan, Mingyuan Zou, Xue Zhou. Nangang CDC – Ziyan Guo, Quan Kang, Yanjie Li, Bo Yu, Qinai Xu.

Henan: Henan Provincial CDC – Liang Chang, Lei Fan, Shixian Feng, Ding Zhang, Gang Zhou. Huixian CDC – Yulian Gao, Tianyou He, Pan He, Chen Hu, Huarong Sun, Xukui Zhang.

Hunan: Hunan Provincial CDC – Biyun Chen, Zhongxi Fu, Yuelong Huang, Huilin Liu, Qiaohua Xu, Li Yin. Liuyang CDC – Huajun Long, Xin Xu, Hao Zhang, Libo Zhang.

Liuzhou: Guangxi Provincial CDC – Naying Chen, Duo Liu, Zhenzhu Tang. Liuzhou CDC – Ningyu Chen, Qilian Jiang, Jian Lan, Mingqiang Li, Yun Liu, Fanwen Meng, Jinhuai Meng, Rong Pan, Yulu Qin, Ping Wang, Sisi Wang, Liuping Wei, Liyuan Zhou.

Qingdao: Qingdao CDC – Liang Cheng, Ranran Du, Ruqin Gao, Feifei Li, Shanpeng Li, Yongmei Liu, Feng Ning, Zengchang Pang, Xiaohui Sun, Xiaocao Tian, Shaojie Wang, Yaoming Zhai, Hua Zhang. Licang CDC – Wei Hou, Silu Lv, Junzheng Wang.

Sichuan: Sichuan Provincial CDC – Xiaofang Chen, Xianping Wu, Ningmei Zhang, Weiwei Zhou. Pengzhou CDC – Xiaofang Chen, Jianguo Li, Jiaqiu Liu, Guojin Luo, Qiang Sun, Xunfu Zhong.

Suzhou: Jiangsu Provincial CDC – Jian Su, Ran Tao, Ming Wu, Jie Yang, Jinyi Zhou, Yonglin Zhou. Suzhou CDC – Yihe Hu, Yujie Hua, Jianrong Jin Fang Liu, Jingchao Liu, Yan Lu, Liangcai Ma, Aiyu Tang, Jun Zhang.

Zhejiang: Zhejiang Provincial CDC – Weiwei Gong, Ruying Hu, Hao Wang, Meng Wang, Min Yu. Tongxiang CDC – Lingli Chen, Qijun Gu, Dongxia Pan, Chunmei Wang, Kaixu Xie, Xiaoyi Zhang.

Lifelines Cohort Study

Behrooz Z Alizadeh[1], H Marike Boezen[1], Lude Franke[2], Pim van der Harst[3], Gerjan Navis[4], Marianne Rots[5], Harold Snieder[1], Morris Swertz[2], Bruce HR Wolffenbuttel[6], and Cisca Wijmenga[2]

1. Department of Epidemiology, University of Groningen, University Medical Center Groningen, The Netherlands
2. Department of Genetics, University of Groningen, University Medical Center Groningen, The Netherlands
3. Department of Cardiology, University of Groningen, University Medical Center Groningen, The Netherlands
4. Department of Internal Medicine, Division of Nephrology, University of Groningen, University Medical Center Groningen, The Netherlands
5. Department of Pathology and Medical Biology, University of Groningen, University Medical Center Groningen, The Netherlands
6. Department of Endocrinology, University of Groningen, University Medical Center Groningen, The Netherlands

Danish Blood Donor Study

Karina Banasik[1], Jakob Bay[2], Jens Kjærgaard Boldsen[3], Thorsten Brodersen[2], Søren Brunak[1], Kristoffer Burgdorf[1], Mona Ameri Chalmer[4], Maria Didriksen[5], Khoa Manh Dinh[3], Joseph Dowsett[5], Christian Erikstrup[3,6], Bjarke Feenstra[5,7], Frank Geller[5,7], Daniel Gudbjartsson[8], Thomas Folkmann Hansen[4], Lotte Hindhede[3], Henrik Hjalgrim[9,7], Rikke Louise Jacobsen[5], Gregor Jemec[10], Bitten Aagaard Jensen[11], Katrine Kaspersen[3], Bertram Dalskov Kjerulff[3], Lisette Kogelman[4], Margit Anita Hørup Larsen[5], Ioannis Louloudis[1], Agnete Lundgaard[1], Susan Mikkelsen[3], Christina Mikkelsen[5], Ioanna Nissen[5], Mette Nyegaard[12], Sisse Rye Ostrowski[5,13], Ole Birger Pedersen[2,13], Alexander Pil Henriksen[1], Palle Duun Rohde[12], Klaus Rostgaard[9,7], Michael Schwinn[5], Kari Stefansson[8], Hreinn Stefánsson[8], Erik Sørensen[5], Unnur Þorsteinsdóttir[8], Lise Wegner Thørner[5], Mie Topholm Bruun[14], Henrik Ullum[15], Thomas Werge[16,13], and David Westergaard[1]

1. Novo Nordisk Foundation Center for Protein Research, Faculty of Health and Medical Sciences, University of Copenhagen, Copenhagen, Denmark,
2. Department of Clinical Immunology, Zealand University Hospital, Køge, Denmark,
3. Department of Clinical Immunology, Aarhus University Hospital, Aarhus, Denmark,
4. Danish Headache Center, Department of Neurology, Copenhagen University Hospital, Rigshospitalet-Glostrup, Copenhagen, Denmark,
5. Department of Clinical Immunology, Copenhagen University Hospital,

Rigshospitalet, Copenhagen, Denmark,
6. Department of Clinical Medicine, Health, Aarhus University, Aarhus, Denmark,
7. Department of Epidemiology Research, Statens Serum Institut, Copenhagen, Denmark,
8. deCODE Genetics, Reykjavik, Iceland,
9. Danish Cancer Society Research Center, Copenhagen, Denmark,
10. Department of Dermatology, Zealand University hospital, Roskilde, Denmark,
11. Department of Clinical Immunology, Aalborg University Hospital, Aalborg, Denmark,
12. Department of Health Science and Technology, Faculty of Medicine, Aalborg Univeristy, Aalborg, Denmark,
13. Department of Clinical Medicine, Faculty of Health and Medical Sciences, University of Copenhagen, Copenhagen, Denmark,
14. Department of Clinical Immunology, Odense University Hospital, Odense, Denmark,
15. Statens Serum Institut, Copenhagen, Denmark,
16. Institute of Biological Psychiatry, Mental Health Centre, Sct. Hans, Copenhagen University Hospital, Roskilde, Denmark.

The Ovarian Cancer Association Consortium

CM Phelan[1], KB Kuchenbaecker[2,3], JP Tyrer[4], SP Kar[4], K Lawrenson[5], SJ Winham[6], J Dennis[4], A Pirie[4], M J Riggan[7], G Chornokur[8], MA Earp[9], PC Lyra, Jr.[8], JM. Lee[10], S Coetzee[10], J Beesley[11], L McGuffog[12], P Soucy[13], E Dicks[4], A Lee[12], D Barrowdale[12], J Lecarpentier[12], G Leslie[12], CM Aalfs[14], KKH Aben[15,16], M Adams[17], J Adlard[18], IL Andrulis[19], H Anton-Culver[20], N Antonenkova[21], AOCS study group[22], G Aravantinos[23], N Arnold[24], BK Arun[25], B Arver[26], J Azzollini[27], J Balmaña[28], SN Banerjee[29], L Barjhoux[30], RB Barkardottir[31,32], Y Bean[33], MW Beckmann[34], A BeeghlyFadiel[35], J Benitez[36], M Bermisheva[37], MQ Bernardini[38], MJ Birrer[39], L Bjorge[40,41], A Black[42], K Blankstein[43], MJ Blok[44], C Bodelon[42], N Bogdanova[45], A Bojesen[46], B Bonanni[47], Å Borg[48], AR Bradbury[49], JD Brenton[50], C Brewer[51], L Brinton[42], P Broberg[52], A Brooks-Wilson[53], F Bruinsma[54], J Brunet[55], B Buecher[56], R Butzow[57], SS Buys[58], T Caldes[59], MA Caligo[60], I Campbell[61,62], R Cannioto[63], ME Carney[64], T Cescon[43], SB Chan[65], J Chang-Claude[66,67], S Chanock[42], X Qing Chen[11], Y-E Chiew[68,69], J Chiquette[70], WK Chung[71], KBM Claes[72], T Conner[58], LS Cook[73], J Cook[74], DW Cramer[75], JM Cunningham[76], AA D'Aloisio[77], MB Daly[78], F Damiola[30], S Dina Damirovna[79], A Dansonka-Mieszkowska[80], F Dao[81], R Davidson[82], A DeFazio[68,69], C Delnatte[83], KF Doheny[17], O Diez[84,85], Y Chun Ding[86], J Anne Doherty[87], SM Domchek[49], CM Dorfling[88], T Dörk[89], L Dossus[90], M Duran[91], M Dürst[92], B Dworniczak[93], D Eccles[94], T Edwards[35], R Eeles[95], U Eilber[66], B Ejlertsen[96], AB Ekici[97], S Ellis[12], M Elvira[79], Study EMBRACE[22], KH Eng[98], C Engel[99], DG Evans[100], PA Fasching[101,34], S Ferguson[38], S Fert Ferrer[102], JM Flanagan[103], ZC Fogarty[6], RT Fortner[66], F Fostira[104], WD Foulkes[105], G Fountzilas[106], BL Fridley[107], TM Friebel[108], E Friedman[109], D Frost[12], PA Ganz[110], J Garber[111], MJ García[36], V GarciaBarberan[59], A Gehrig[112], GEMO Study Collaborators[22], A GentryMaharaj[113], A-M Gerdes[114], GG Giles[54,115,116], R Glasspool[117], G Glendon[118], AK Godwin[119], DE Goldgar[120], T Goranova[50], M Gore[121], MH Greene[122], J Gronwald[123], S Gruber[124], E Hahnen[125], CA Haiman[126], N Håkansson[127], U Hamann[128], TVO Hansen[129], PA Harrington[4], HR Harris[127], J Hauke[125] HEBON Study[22], A Hein[34], A Henderson[130], MAT Hildebrandt[131], P Hillemanns[132], S Hodgson[133], CK Høgdall[134], E Høgdall[135,136], FBL Hogervorst[137], H

Holland[11], MJ Hooning[138], K Hosking[4], R-Y Huang[139], PJ Hulick[140], J Hung[68,69], DJ Hunter[141], DG Huntsman[142], T Huzarski[123], EN Imyanitov[143], C Isaacs[144], ES Iversen[145], L Izatt[146], A Izquierdo[55], A Jakubowska[123], P James[147], R Janavicius[148,149], M Jernetz[150], A Jensen[135], U Birk Jensen[151], EM John[152], S Johnatty[11], ME Jones[153], P Kannisto[150], BY Karlan[5], A Karnezis[142], K Kast[154], KconFab Investigators[22], CJ Kennedy[68,69], E Khusnutdinova[37], LA Kiemeney[15], JI Kiiski[155], S-W Kim[156], SK Kjaer[134,135], M Köbel[157], RK Kopperud[40,41], TA Kruse[158], J Kupryjanczyk[80], A Kwong[159,160,161], Y Laitman[109], D Lambrechts[162,163], N Larrañaga[164,165], MC Larson[6], C Lazaro[166] ND Le[167] L Le Marchand[168], J Won Lee[169], SB Lele[170], A Leminen[155], D Leroux[171], J Lester[5], F Lesueur[172], DA Levine[81], D Liang[173], C Liebrich[174], J Lilyquist[175], L Lipworth[176], J Lissowska[177], KH Lu[178], J Lubiński[123], C Luccarini[4], L Lundvall[179], PL Mai[122], G Mendoza-Fandiño[8], S Manoukian[27], LFAG Massuger[15], T May[38], S Mazoyer[180], JN McAlpine[181], V McGuire[182], JR McLaughlin[183], I McNeish[184], H MeijersHeijboer[185], A Meindl[186], U Menon[113], AR Mensenkamp[187], MA Merritt[188], RL Milne[54,115], G Mitchell[147,189], F Modugno[190,191,192], J Moes-Sosnowska[80], M Moffitt[193,194], M Montagna[195], KB Moysich[63], AM Mulligan[196,197], J Musinsky[198], KL Nathanson[49], L Nedergaard[199], RB Ness[200], SL Neuhausen[86], H Nevanlinna[155], D Niederacher[201], RL Nussbaum[202], K Odunsi[170], E Olah[203], OI Olopade[204], H Jernström[205], C Olswold[175], DM O'Malley[206], K Ong[207], NC Onland-Moret[208], OPAL study group[22], N Orr[209], S Orsulic[5], A Osorio[36], D Palli[210], L Papi[211], T-W Park-Simon[132], J Paul[212], CL Pearce[213,126], I Søkilde Pedersen[214], PHM Peeters[208], B Peissel[27], A Peixoto[215], T Pejovic[193,194], LM Pelttari[155], JB Permuth[8], P Peterlongo[216], L Pezzani[27], G Pfeiler[217], K-A Phillips[189,115,218], M Piedmonte[219], MC Pike[126,220], AM Piskorz[50], SR Poblete[170], T Pocza[203], EM Poole[221], B Poppe[72], ME Porteous[222], F Prieur[223], D Prokofyeva[79], E Pugh[17], M Angel Pujana[224], P Pujol[225], P Radice[226], J Rantala[227], C Rappaport-Fuerhauser[217], G Rennert[228], K Rhiem[125], P Rice[43], A Richardson[229], M Robson[230], GC Rodriguez[231], C Rodríguez-Antona[232], J Romm[17], MA Rookus[233], M Anne Rossing[234], JH Rothstein[182], A Rudolph[66], IB Runnebaum[92], HB Salvesen[40,41], DP Sandler[235], MJ Schoemaker[153], L Senter[236], VW Setiawan[126], G Severi[237,238,239,240], P Sharma[241], T Shelford[17], N Siddiqui[242], LE Side[243], W Sieh[182], CF Singer[217], H Sobol[244], H Song[4], MC Southey[245], AB Spurdle[11], Z Stadler[230], D Steinemann[246], D Stoppa-Lyonnet[56], LE Sucheston-Campbell[247], G Sukiennicki[123], R Sutphen[248], C Sutter[249], AJ Swerdlow[153,250], CI Szabo[251], L Szafron[80], YY Tan[217], JA Taylor[235], M-K Tea[217], MR Teixeira[252], S-H Teo[253,254], KL Terry[75,255], PJ Thompson[256], LCV Thomsen[40,41], DL Thull[257], L Tihomirova[258] AV Tinker[259], M Tischkowitz[105,260], S Tognazzo[195], A Ewart Toland[261], A Tone[38], B Trabert[42], RC Travis[262], A Trichopoulou[263,264], N Tung[265], SS. Tworoger[221,255], AM van Altena[266], D Van Den Berg[126], AH van der Hout[267], RB van der Luijt[268], M Van Heetvelde[72], E Van Nieuwenhuysen[269], EJ van Rensburg[88], A Vanderstichele[269], R Varon-Mateeva[270], V Ana[271,272], D Velez Edwards[273], I Vergote[269], RA Vierkant[6], J Vijai[198], A Vratimos[104], L Walker[274], C Walsh[5], D Wand[275], S Wang-Gohrke[276], B Wappenschmidt[125], PM Webb[277], CR Weinberg[278], JN Weitzel[279], N Wentzensen[42], AS Whittemore[182,280], JT Wijnen[281], LR Wilkens[168], A Wolk[127], M Woo[142], X Wu[131], AH Wu[126], H Yang[42], D Yannoukakos[104], A Ziogas[282], KK Zorn[257], SA Narod[283], DF Easton[3,4], CI Amos[284], JM Schildkraut[28,5], SJ Ramus[286,287], L Ottini[288], MT Goodman[256,289], SK Park[290,291,292], LE Kelemen[293], HA Risch[294], M Thomassen[158], K Offit[198], J Simard[13], R Katharina Schmutzler[125], D Hazelett[295], AN Monteiro[8], FJ Couch[76], A Berchuck[7], G Chenevix-Trench[11], EL Goode[9], TA. Sellers[8], SA Gayther[10], AC Antoniou[3,4], and PDP Pharoah[3,4]

1. Departments of Cancer Epidemiology and Gynecologic Oncology, Moffitt Cancer Center, Tampa, FL, USA
2. Wellcome Trust Sanger Institute, Wellcome Genome Campus, Hinxton, Cambridgeshire CB10 1SA, UK
3. Centre for Cancer Genetic Epidemiology, Department of Public Health and Primary Care, University of Cambridge, Strangeways Research Laboratory, Worts Causeway, Cambridge, UK
4. Department of Oncology, Centre for Cancer Genetic Epidemiology, University of Cambridge, Strangeways Research Laboratory, Cambridge, UK
5. Women's Cancer Program at the Samuel Oschin Comprehensive Cancer Institute, CedarsSinai Medical Center, 8700 Beverly Boulevard, Suite 290W, Los Angeles, CA, USA
6. Department of Health Science Research, Division of Biomedical Statistics and Informatics, Mayo Clinic, Rochester, Minnesota, USA
7. Department of Obstetrics and Gynecology, Duke University Medical Center, Durham, North Carolina, USA
8. Department of Cancer Epidemiology, Moffitt Cancer Center, Tampa, FL, USA
9. Department of Health Science Research, Division of Epidemiology, Mayo Clinic, Rochester, Minnesota, USA
10. Center for Bioinformatics and Functional Genomics, Samuel Oschin Comprehensive Cancer Institute, Cedars-Sinai Medical Center, Los Angeles, California, USA
11. Department of Genetics and Computational Biology, QIMR Berghofer Medical Research Institute, Brisbane, QLD, Australia
12. Centre for Cancer Genetic Epidemiology, Department of Public Health and Primary Care, University of Cambridge, Cambridge, UK
13. Genomics Center, Centre Hospitalier Universitaire de Québec Research Center and Laval University, 2705 Laurier Boulevard, Quebec City (Quebec), Canada
14. Department of Clinical Genetics, Academic Medical Center, Amsterdam, The Netherlands
15. Radboud university medical center, Radboud Institute for Health Sciences, Department for Health Evidence, Nijmegen, The Netherlands
16. Netherlands Comprehensive Cancer Organisation, Utrecht, The Netherlands
17. Center for Inherited Disease Research, Institute of Genetic Medicine, Johns Hopkins University School of Medicine, Baltimore, MD 21224
18. Yorkshire Regional Genetics Service, Chapel Allerton Hospital, Leeds, UK
19. Lunenfeld-Tanenbaum Research Institute, Mount Sinai Hospital, Toronto, Ontario M5G 1X5, Departments of Molecular Genetics and Laboratory Medicine and Pathobiology, University of Toronto, Ontario, Canada
20. Department of Epidemiology, Director of Genetic Epidemiology Research Institute, UCI Center for Cancer Genetics Research & Prevention, School of Medicine, University of California Irvine, Irvine, California, USA
21. N.N. Alexandrov National Cancer Centre of Belarus, Minsk, Belarus
22. A list of members and affiliations appears in the Supplementary note
23. "Agii Anargiri" Cancer Hospital, Athens, Greece
24. Department of Gynaecology and Obstetrics, University Hospital of Schleswig-Holstein, Campus Kiel, Christian-Albrechts University Kiel, Germany
25. Department of Breast Medical Oncology and Clinical Cancer Genetics Program, University Of Texas MD Andersson Cancer Center, 1515 Pressler Street, CBP 5, Houston, TX, USA

26. Department of Oncology and Pathology, Karolinska University Hospital and Karolinska Institutet, Stockholm, Sweden
27. Unit of Medical Genetics, Department of Preventive and Predictive Medicine, Fondazione IRCCS (Istituto Di Ricovero e Cura a Carattere Scientifico) Istituto Nazionale Tumori (INT), Via Giacomo Venezian 1, 20133 Milan, Italy
28. Department of Medical Oncology, University Hospital, Vall d'Hebron, Barcelona, Spain
29. Gynaecology Unit, The Royal Marsden Hospital, London, UK
30. Bâtiment Cheney D, Centre Léon Bérard, 28 rue Laënnec, Lyon, France
31. Laboratory of Cell Biology, Department of Pathology, hus 9, Landspitali-LSH v/Hringbraut, 101 Reykjavik, Iceland
32. BMC (Biomedical Centre), Faculty of Medicine, University of Iceland, Vatnsmyrarvegi 16, 101 Reykjavik, Iceland
33. Department of Gynecologic Oncology, Oregon Health & Science University, Portland, OR, USA; Knight Cancer Institute, Portland, OR, USA
34. Universtiy Hospital Erlangen, Department of Gynecology and Obstetrics, FriedrichAlexander-University Erlangen-Nuremberg, Comprehensive Cancer Center Erlangen Nuremberg, Universitaetsstrasse 21-23, 91054 Erlangen, Germany
35. Division of Epidemiology, Department of Medicine, Vanderbilt Epidemiology Center, Institute for Medicine and Public Health, Vanderbilt University Medical Center, VanderbiltIngram Cancer Center, Nashville, TN, USA
36. Human Genetics Group, Spanish National Cancer Centre (CNIO), and Biomedical Network on Rare Diseases (CIBERER), Madrid, Spain
37. Institute of Biochemistry and Genetics, Ufa Science Center, Russian Academy of Sciences, Ufa, Bashkortostan, Russia
38. Division of Gynecologic Oncology, Princess Margaret Hospital, University Health Network, Toronto, Ontario, Canada
39. Department of Medicine, Massachusetts General Hospital, Boston, USA
40. Department of Gynecology and Obstetrics, Haukeland University Hospital, Bergen, Norway
41. Centre for Cancer Biomarkers, Department of Clinical Science, University of Bergen, Bergen, Norway
42. Division of Cancer Epidemiology and Genetics, National Cancer Institute, Bethesda, MD, USA
43. Clinical Cancer Genetics, for the City of Hope Clinical Cancer Genetics Community Research Network, Duarte, CA, USA
44. Department of Clinical Genetics, Maastricht University Medical Center, Maastricht, The Netherlands
45. Radiation Oncology Research Unit, Hannover Medical School, Hannover, Germany
46. Department of Clinical Genetics, Vejle Hospital, Vejle, Denmark
47. Division of Cancer Prevention and Genetics, Istituto Europeo di Oncologia (IEO), via Ripamonti 435, 20141 Milan, Italy
48. Department of Oncology, Clinical Sciences, Lund University and Skåne University Hospital, Lund, Sweden
49. Department of Medicine, Abramson Cancer Center, Perelman School of Medicine at The University of Pennsylvania, Philadelphia, PA, USA
50. Cancer Research UK (CRUK) Cambridge Institute, University of Cambridge
51. Department of Clinical Genetics, Royal Devon and Exeter Hospital, Exeter, UK
52. Department of Cancer Epidemiology, University Hospital, Lund, Lund University,

Lund, Sweden
53. Canada’s Michael Smith Genome Sciences Centre, BC Cancer Agency, Vancouver, BC, Canada
54. Cancer Epidemiology Centre, Cancer Council Victoria, Melbourne, VIC, Australia
55. Genetic Counseling Unit, Hereditary Cancer Program, IDIBGI (Institut d’Investigació Biomèdica de Girona), Catalan Institute of Oncology. Av. França s/n. 1707 Girona, Spain
56. Service de Génétique Oncologique, Institut Curie, 26, rue d’Ulm, Paris Cedex 05, France
57. Department of Pathology, University of Helsinki and Helsinki University Hospital, Helsinki, Finland
58. Department of Medicine, Huntsman Cancer Institute, 2000 Circle of Hope, Salt Lake City, UT 84112, USA
59. Molecular Oncology Laboratory, Hospital Clinico San Carlos, IdISSC (Instituto de Investigación Sanitaria del Hospital Clínico San Carlos), Martin Lagos s/n, Madrid, Spain
60. Section of Genetic Oncology, Dept. of Laboratory Medicine, University and University Hospital of Pisa, Pisa Italy
61. Cancer Genetics Laboratory, Research Division, Peter MacCallum Cancer Centre, Melbourne, VIC, Australia
62. Department of Pathology, University of Melbourne, Parkville, VIC, Australia
63. Cancer Pathology & Prevention, Division of Cancer Prevention and Population Sciences, Roswell Park Cancer Institute, Buffalo, NY
64. Department of Obstetrics and Gynecology, John A. Burns School of Medicine, University of Hawaii, Honolulu, Hawaii, US
65. University of California, San Francisco, 1600 Divisadero Street, C415, San Francisco, CA 94143 – 1714, USA
66. Division of Cancer Epidemiology, German Cancer Research Center (DKFZ), Heidelberg, Germany
67. University Cancer Center Hamburg (UCCH), University Medical Center HamburgEppendorf, Hamburg, Germany
68. Centre for Cancer Research, The Westmead Institute for Medical Research, The University of Sydney, Sydney, New South Wales, Australia
69. Department of Gynaecological Oncology, Westmead Hospital, Sydney, New South Wales, Australia
70. Unité de Recherche en Santé des Populations, Centre des Maladies du Sein DeschênesFabia, Centre de Recherche FRSQ du Centre Hospitalier Affilié Universitaire de Québec, Québec, QC, Canada
71. Departments of Pediatrics and Medicine, Columbia University, New York, NY, USA
72. Center for Medical Genetics, Ghent University, Ghent, Belgium
73. Division of Epidemiology, Biostatistics and Preventative Medicine, Department of Internal Medicine, University of New Mexico, Albuquerque, New Mexico, USA
74. Sheffield Clinical Genetics Service, Sheffield Children’s Hospital, Sheffield, UK
75. Obstetrics and Gynecology Epidemiology Center, Brigham and Women’s Hospital and Harvard Medical School, Boston, MA, USA
76. Department of Laboratory Medicine and Pathology,Division of Experimental Pathology, Mayo Clinic, Rochester, MN, USA
77. Social & Scientific Systems, Inc. Durham, NC 27703, USA
78. Department of Clinical Genetics, Fox Chase Cancer Center, 333 Cottman

Avenue, Philadelphia, PA 19111, USA
79. Department of Genetics and Fundamental Medicine, Bashkir State University, Ufa, Russia
80. Department of Pathology and Laboratory Diagnostics, the Maria Sklodowska-Curie Memorial Cancer Center and Institute of Oncology, Warsaw, Poland
81. Gynecology Service, Department of Surgery, Memorial Sloan Kettering Cancer Center, New York, NY, USA
82. Department of Clinical Genetics, South Glasgow University Hospitals, Glasgow, UK
83. Unité d'oncogénétique, ICO-Centre René Gauducheau, Boulevard Jacques Monod, 44805 Nantes Saint Herblain Cedex, France
84. Oncogenetics Group, Vall d'Hebron Institute of Oncology (VHIO), Barcelona, Spain
85. Clinical and Molecular Genetics Area, Vall d'Hebron University Hospital. Barcelona, Spain
86. Department of Population Sciences, Beckman Research Institute of City of Hope, Duarte, CA, USA
87. Department of Epidemiology, The Geisel School of Medicine – at Dartmouth, Hanover, New Hampshire, USA
88. Cancer Genetics Laboratory, Department of Genetics, University of Pretoria, Pretoria, South Africa
89. Gynaecology Research Unit, Hannover Medical School, Hannover, Germany
90. Nutrition and Metabolism Section, International Agency for Research on Cancer (IARCWHO), Lyon, France
91. Institute of Biology and Molecular Genetics, Universidad de Valladolid (IBGM-UVA), Valladolid, Spain
92. Department of Gynecology, Jena University Hospital – Friedrich Schiller University, Jena, Germany
93. Institute of Human Genetics, Münster, Germany
94. University of Southampton Faculty of Medicine, Southampton University Hospitals NHS Trust, Southampton, UK
95. Oncogenetics Team, The Institute of Cancer Research and Royal Marsden NHS Foundation Trust, Sutton, UK
96. Department of Oncology, Rigshospitalet, Copenhagen University Hospital, Copenhagen, Denmark
97. Institute of Human Genetics, Friedrich-Alexander-University Erlangen-Nuremberg, Erlangen, Germany
98. Department of Biostatistics & Bioinformatics, Roswell park Institute, Buffalo, NY, USA
99. Institute for Medical Informatics, Statistics and Epidemiology, University of Leipzig, Leipzig, Germany
100. Genomic Medicine, Manchester Academic Health Sciences Centre, University of Manchester, Central Manchester University Hospitals NHS Foundation Trust, Manchester, UK
101. University of California at Los Angeles, David Geffen School of Medicine, Department of Medicine, Division of Hematology and Oncology, Los Angeles, CA, USA
102. Laboratoire de Génétique Chromosomique, Hôtel Dieu Centre Hospitalier, Chambéry, France
103. Department of Surgery and Cancer, Imperial College London, London, UK

104. Molecular Diagnostics Laboratory, INRASTES, National Centre for Scientific Research “Demokritos”, Aghia Paraskevi Attikis, Athens, Greece
105. Program in Cancer Genetics, Departments of Human Genetics and Oncology, McGill University, Montreal, Quebec, Canada
106. Department of Medical Oncology, Papageorgiou, Hospital, Aristotle University of Thessaloniki School of Medicine, Thessaloniki, Greece
107. Biostatistics and Informatics Shared Resource, University of Kansas Medical Center, Kansas City, KS, USA
108. Harvard TH Chan School of Public Health and Dana Farber Cancer Institute, 1101 Dana Building, 450 Brookline Ave, Boston, MA 02215, USA
109. The Susanne Levy Gertner Oncogenetics Unit, Institute of Human Genetics, Chaim Sheba Medical Center, Ramat Gan 52621, and Sackler Faculty of Medicine, Tel Aviv University, Ramat Aviv 69978, Israel
110. UCLA Schools of Medicine and Public Health, Division of Cancer Prevention and Control Research, Jonsson Comprehensive Cancer Center, Los Angeles, CA, USA
111. Cancer Risk and Prevention Clinic, Dana Farber Cancer Institute, Boston, MA, USA
112. Centre of Familial Breast and Ovarian Cancer, Department of Medical Genetics, Institute of Human Genetics, University Würzburg, Germany
113. Women’s Cancer, Institute for Women’s Health, University College London, London, United Kingdom
114. Department of Clinical Genetics, Rigshospitalet, Copenhagen University Hospital, Copenhagen, Denmark
115. Centre for Epidemiology and Biostatistics, School of Population and Global Health, University of Melbourne, Australia
116. Department of Epidemiology and Preventive Medicine, Monash University, Melbourne, VIC, Australia
117. The Beatson West of Scotland Cancer Centre, Glasgow, UK
118. Ontario Cancer Genetics Network: Samuel Lunenfeld Research Institute, Mount Sinai Hospital, Toronto, Ontario M5G 1X5
119. Department of Pathology and Laboratory Medicine, University of Kansas Medical Center, Kansas City, KS, USA
120. Department of Dermatology, University of Utah School of Medicine, Salt Lake City, Utah, USA
121. Department of Medicine, Royal Marsden Hospital, London, UK
122. Clinical Genetics Branch,DCEG, NCI, NIH, 9609 Medical Center Drive, Room 6E-454, Bethesda, MD, USA
123. Department of Genetics and Pathology, Pomeranian Medical University, Szczecin, Poland
124. Keck School of Medicine, and Norris Comprehensive Cancer Center, University of Southern California, Los Angeles, CA
125. Center for Familial Breast and Ovarian Cancer, Center for Integrated Oncology (CIO) and Center for Molecular Medicine Cologne (CMMC), University Hospital Cologne, Cologne, Germany
126. Department of Preventive Medicine, Keck School of Medicine, University of Southern California Norris Comprehensive Cancer Center, Los Angeles, CA, USA
127. Karolinska Institutet, Department of Environmental Medicine, Division of Nutritional Epidemiology, SE-171 77 STOCKHOLM, Sweden
128. Molecular Genetics of Breast Cancer, Deutsches Krebsforschungszentrum

(DKFZ), Heidelberg, Germany
129. Center for Genomic Medicine, Rigshospitalet, Copenhagen University Hospital, Copenhagen, Denmark
130. Institute of Genetic Medicine, Centre for Life, Newcastle Upon Tyne Hospitals NHS Trust, Newcastle upon Tyne, UK
131. Department of Epidemiology, The University of Texas MD Anderson Cancer Center, Houston, TX, USA
132. Clinics of Obstetrics and Gynaecology, Hannover Medical School, Hannover, Germany
133. Medical Genetics Unit, St George's, University of London, UK
134. Department of Gynecology, Rigshospitalet, University of Copenhagen, Copenhagen, Denmark
135. Department of Virus, Lifestyle and Genes, Danish Cancer Society Research Center, Copenhagen, Denmark
136. Molecular Unit, Department of Pathology, Herlev Hospital, University of Copenhagen, Copenhagen, Denmark
137. Family Cancer Clinic, Netherlands Cancer Institute, Amsterdam, The Netherlands
138. Department of Medical Oncology, Family Cancer Clinic, Erasmus University Medical Center, Rotterdam, The Netherlands
139. Center For Immunotherapy, Roswell Park Cancer Institute, Buffalo, NY, USA
140. Center for Medical Genetics, NorthShore University HealthSystem, University of Chicago Pritzker School of Medicine, 1000 Central Street, Suite 620,Evanston, IL 60201,US
141. Program in Genetic Epidemiology and Statistical Genetics, Department of Epidemiology, The Harvard T.H. Chan School of Public Health, 677 Huntington Avenue, Boston, MA, 02115, USA
142. British Columbia's Ovarian Cancer Research (OVCARE) Program, Vancouver General Hospital, BC Cancer Agency and University of British Columbia; Departments of Pathology and Laboratory Medicine, Obstetrics and Gynaecology and Molecular Oncology, Vancouver, British Columbia, CANADA
143. N.N. Petrov Institute of Oncology, St.Petersburg, Russia
144. Lombardi Comprehensive Cancer Center, Georgetown University, 3800 Reservoir Road NW, Washington, DC, USA
145. Department of Statistical Science, Duke University, Durham, North Carolina, USA
146. Clinical Genetics, Guy's and St. Thomas' NHS Foundation Trust, London, UK
147. Familial Cancer Centre, Peter MacCallum Cancer Centre, Locked Bag 1, A'Beckett Street, Melbourne, VIC 8006 AUSTRALIA; Sir Peter MacCallum Dept of Oncology, University of Melbourne, VIC 3010
148. Vilnius University Hospital Santariskiu Clinics, Hematology, Oncology and Transfusion Medicine Center, Department of Molecular and Regenerative Medicine, Vilnius, Lithuania
149. State Research Institute Centre for Innovative Medicine, Vilnius, Lithuania
150. Department of Obstetrics and Gynecology Lund University Hospital, Lund Sweden
151. Department of Clinical Genetics, Aarhus University Hospital, Brendstrupgaardsvej 21C, Aarhus N, Denmark
152. Department of Epidemiology, Cancer Prevention Institute of California, Fremont, California, USA

153. Division of Genetics and Epidemiology, Institute of Cancer Research, London, UK
154. Department of Gynaecology and Obstetrics, University Hospital Carl Gustav Carus, Technical University Dresden, Germany
155. Department of Obstetrics and Gynecology, University of Helsinki and Helsinki University Central Hospital, Helsinki, HUS, Finland
156. Department of Surgery, Breast Care Center, Daerim St. Mary’s Hospital, 657 Siheungdaero, Yeongdeungpo-gu, Seoul, 150-822, Korea
157. Department of Pathology, University of Calgary, Calgary, Alberta, Canada
158. Department of Clinical Genetics, Odense University Hospital, Odense C, Denmark
159. The Hong Kong Hereditary Breast Cancer Family Registry, Hong Kong
160. Department of Surgery, The University of Hong Kong, Hong Kong
161. Cancer Genetics Center and Department of Surgery, Hong Kong Sanatorium and Hospital, Hong Kong
162. Vesalius Research Center, VIB, Leuven, Belgium
163. Laboratory for Translational Genetics, Department of Oncology, KULeuven, Belgium
164. Public Health Division of Gipuzkoa, Regional Government of the Basque Country, Spain
165. CIBER of Epidemiology and Public Health (CIBERESP), Spain
166. Molecular Diagnostic Unit, Hereditary Cancer Program, IDIBELL-Catalan Institute of Oncology, Barcelona, Spain
167. Cancer Control Research, BC Cancer Agency, Vancouver, BC, Canada
168. Cancer Epidemiology Program, University of Hawaii Cancer Center, Hawaii, USA
169. Department of Surgery, Ulsan College of Medicine and Asan Medical Center, Seoul, Korea
170. Department of Gynecological Oncology, Roswell Park Cancer Institute, Buffalo, NY, USA
171. Département de Génétique, Centre Hospitalier Universitaire de Grenoble, BP 217, Grenoble Cedex 9, France
172. Institut Curie, PSL Research Unviersity and Inserm, U900, Paris, France; Mines Paris Tech, Fontainebleau, France
173. College of Pharmacy and Health Sciences, Texas Southern University, Houston, Texas, USA
174. Cancer Center Wolfsburg, Clinics of Gynaecology, Wolfsburg, Germany
175. Department of Health Sciences Research, Division of Epidemiology, Mayo Clinic, Rochester, MN, USA
176. Division of Epidemiology, Department of Medicine, Vanderbilt Epidemiology Center and Vanderbilt-Ingram Cancer Center, Vanderbilt University School of Medicine, Nashville, TN, USA
177. Department of Cancer Epidemiology and Prevention, Maria Sklodowska-Curie Memorial Cancer Center and Institute of Oncology, Warsaw, Poland
178. Department of Gynecologic Oncology, The University of Texas MD Anderson Cancer Center, Houston, TX, USA
179. The Juliane Marie Centre, Department of Gynecology, Rigshospitalet, University of Copenhagen, Copenhagen, Denmark
180. INSERM U1052, CNRS UMR5286, Université Lyon 1, Centre de Recherche en Cancérologie de Lyon, Centre Léon Bérard, Lyon, France

181. Ovarian Cancer Research (OVCARE) Program – Gynecologic Tissue Bank, Vancouver General Hospital and BC Cancer Agency, Vancouver, British Columbia CANADA
182. Department of Health Research and Policy – Epidemiology, Stanford University School of Medicine, Stanford, CA, USA
183. Public Health Ontario, Toronto, ON, Canada
184. Institute of Cancer Sciences, University of Glasgow, Wolfson Wohl Cancer Research Centre, Beatson Institute for Cancer Research, Glasgow, UK
185. Department of Clinical Genetics, VU University Medical Centre, P.O. Box 7057, 1007 MB Amsterdam, the Netherlands
186. Department of Gynaecology and Obstetrics, Division of Tumor Genetics, Klinikum rechts der Isar, Technical University of Munich, Munich, Germany
187. Department of Human Genetics, Radboud University Medical Centre, Nijmegen, The Netherlands
188. Department of Epidemiology and Biostatistics, School of Public Health, Imperial College London, London, W2 1PG, UK
189. Sir Peter MacCallum Department of Oncology, University of Melbourne, Parkville, VIC, Australia
190. Division of Gynecologic Oncology, Department of Obstetrics, Gynecology and Reproductive Sciences, University of Pittsburgh School of Medicine, Pittsburgh, Pennsylvania, USA
191. Department of Epidemiology, University of Pittsburgh Graduate School of Public Health, Pittsburgh, Pennsylvania, USA
192. Ovarian Cancer Center of Excellence, Womens Cancer Research Program, MageeWomens Research Institute and University of Pittsburgh Cancer Institute, Pittsburgh, Pennsylvania, USA
193. Department of Obstetrics and Gynecology, Oregon Health and Science University, Portland, OR, USA
194. Knight Cancer Institute, Oregon Health & Science University, Portland, Oregon, USA
195. Immunology and Molecular Oncology Unit, Veneto Institute of Oncology IOV – IRCCS, Via Gattamelata 64, Padua, Italy
196. Department of Laboratory Medicine and Pathobiology, University of Toronto, Toronto, ON, Canada
197. Department of Laboratory Medicine, and the Keenan Research Centre of the Li Ka Shing Knowledge Institute, St Michael’s Hospital, Toronto, ON, Canada
198. Clinical Genetics Research Laboratory, Department of Medicine, Memorial Sloan Kettering Cancer Center, 1275 York Avenue, New York, NY 10044, USA
199. Department of Pathology, Rigshospitalet, University of Copenhagen, Denmark
200. The University of Texas School of Public Health, Houston, TX, USA
201. Department of Gynaecology and Obstetrics, University Hospital Düsseldorf, HeinrichHeine University Düsseldorf, Germany
202. Invitae Corporation and University of Southern California, San Francisco, 513 Parnassus Ave., HSE 901E, San Francisco, CA. 94143 – 0794
203. Department of Molecular Genetics, National Institute of Oncology, Budapest, Hungary
204. Center for Clinical Cancer Genetics and Global Health, University of Chicago Medical Center, 5841 South Maryland Avenue, MC 2115 Chicago, IL, USA
205. Oncology, Clinical Sciences in Lund, Lund University, 221 85 Lund, Sweden
206. The Ohio State University and the James Cancer Center, Columbus, Ohio, USA

207. West Midlands Regional Genetics Service, Birmingham Women's Hospital Healthcare NHS Trust, Edgbaston, Birmingham, UK
208. Julius Center for Health Sciences and Primary Care, UMC Utrecht, Utrecht, the Netherlands
209. The Breast Cancer Now Toby Robins Research Centre, The Institute of Cancer Research, 237 Fulham Road, London SW3 6JB, UK
210. Molecular and Nutritional Epidemiology Unit, Cancer Research and Prevention Institute ISPO, Florence, Italy
211. Unit of Medical Genetics, Department of Biomedical, Experimental and Clinical Sciences, University of Florence, Florence, Italy
212. Cancer Research UK Clinical Trials Unit, Institute of Cancer Sciences, University of Glasgow, UK
213. Department of Epidemiology, University of Michigan School of Public Health, Ann Arbor, Michigan, USA
214. Section of Molecular Diagnostics, Clinical Biochemistry, Aalborg University Hospital, Aalborg, Denmark
215. Department of Genetics, Portuguese Oncology Institute, Rua Dr. António Bernardino de Almeida, 4200-072 Porto, Portugal
216. IFOM, The FIRC (Italian Foundation for Cancer Research) Institute of Molecular Oncology, c/o IFOM-IEO campus, via Adamello 16 20139 Milan, Italy
217. Dept of OB/GYN, Medical University of Vienna and Comprehensive Cancer Center, Vienna, Austria, Waehringer Guertel 18-20, A 1090 Vienna, Austria
218. Division of Cancer Medicine, Peter MacCallum Cancer Centre, Locked Bag 1, A'Beckett St, East Melbourne, victoria 8006, Australia
219. NRG Oncology, Statistics and Data Management Center, Roswell Park Cancer Institute, Elm St & Carlton St, Buffalo, NY 14263, USA
220. Department of Epidemiology and Biostatistics, Memorial Sloan Kettering Cancer Center, New York, NY, USA
221. Channing Division of Network Medicine, Brigham and Women's Hospital and Harvard Medical School, Boston, MA, USA
222. South East of Scotland Regional Genetics Service, Western General Hospital, Edinburgh, UK
223. Service de Génétique Clinique Chromosomique et Moléculaire, Centre Hospitalier Universitaire de St Etienne, St Etienne, France
224. Translational Research Laboratory, IDIBELL (Bellvitge Biomedical Research Institute),Catalan Institute of Oncology, Barcelona, Spain
225. Unité d'Oncogénétique, CHU Arnaud de Villeneuve, Montpellier, France
226. Unit of "Predictive Medicine: Molecular Bases of Genetic Risk", Department of Experimental Oncology, Fondazione IRCCS Istituto Nazionale dei Tumori, Milan, Italy
227. Department of Clinical Genetics, Karolinska University Hospital, Stockholm, Sweden
228. Clalit National Israeli Cancer Control Center and Department of Community Medicine and Epidemiology, Carmel Medical Center and B. Rappaport Faculty of Medicine, Haifa, Israel
229. Brigham and Women's Hospital, Dana-Farber Cancer Institute, Boston, MA USA
230. Clinical Genetics Service, Department of Medicine, Memorial Sloan Kettering Cancer Center, New York, NY, USA
231. Division of Gynecologic Oncology, NorthShore University HealthSystem,

University of Chicago, Evanston, IL, USA
232. Hereditary Endocrine Cancer group, Spanish National Cancer Research Center (CNIO), and Biomedical Network on Rare Diseases (CIBERER), Madrid, Spain
233. Department of Epidemiology, Netherlands Cancer Institute, Amsterdam, The Netherlands 234. Program in Epidemiology, Division of Public Health Sciences, Fred Hutchinson Cancer Research Center, Seattle, WA, USA
235. Epidemiology Branch, Division of Intramural Research, National Institute of Environmental Health Sciences, NIH, Research Triangle Park, NC, USA
236. Clinical Cancer Genetics Program, Division of Human Genetics, Department of Internal Medicine, The Comprehensive Cancer Center, The Ohio State University, Columbus, USA
237. Université Paris-Saclay, Université Paris-Sud, UVSQ, CESP, INSERM, Villejuif, France
238. Gustave Roussy, F-94805, Villejuif, France
239. Human Genetics Foundation (HuGeF), Torino, Italy
240. Cancer Council Victoria and University of Melbourne, Australia
241. Department of Hematology and Oncology, University of Kansas Medical Center, Kansas City, KS, USA
242. Department of Gynaecological Oncology, Glasgow Royal Infirmary, Glasgow, UK
243. North East Thames Regional Genetics Service, Great Ormond Street Hospital for Children NHS Trust, London, UK
244. Département Oncologie Génétique, Prévention et Dépistage, INSERM CIC-P9502, Institut Paoli-Calmettes/Université d'Aix-Marseille II, Marseille, France
245. Genetic Epidemiology Laboratory, Department of Pathology, University of Melbourne, Parkville, VIC, Australia
246. Institute of Human Genetics, Hannover Medical School, Hannover, Germany
247. Department of Cancer Prevention and Control, Roswell Park Cancer Institute, Buffalo, NY, USA
248. Epidemiology Center, College of Medicine, University of South Florida, Tampa, Florida, USA
249. Institute of Human Genetics, Department of Human Genetics, University Hospital Heidelberg, Germany
250. Division of Breast Cancer Research, The Institute of Cancer Research, London, UK
251. National Human Genome Research Institute, National Institutes of Health, Bethesda, MD, USA
252. Department of Genetics, Portuguese Oncology Institute, Rua Dr. António Bernardino de Almeida, 4200-072 Porto, Portugal and Biomedical Sciences Institute (ICBAS), University of Porto, Porto, Portugal
253. Cancer Research Initiatives Foundation, Sime Darby Medical Centre, Subang Jaya, Malaysia
254. University Malaya Cancer Research Institute, Faculty of Medicine, University Malaya Medical Centre, University Malaya, Kuala Lumpur, Malaysia
255. Department of Epidemiology, Harvard T. Chan School of Public Health, Boston, MA, USA
256. Cancer Prevention and Control, Samuel Oschin Comprehensive Cancer Institute, CedarsSinai Medical Center, Los Angeles, CA, USA
257. Magee-Womens Hospital of UPMC, University of Pittsburgh School of Medicine, Pittsburgh, PA, USA

258. Latvian Biomedical Research and Study Centre. Ratsupites str 1, Riga, Latvia
259. Ovarian Cancer Research (OVCARE) Program – Cheryl Brown Ovarian Cancer Outcomes Unit (CBOCOU), BC Cancer Agency, Vancouver, British Columbia CANADA
260. Department of Medical Genetics, Box 134, Level 6 Addenbrooke's Treatment Centre, Addenbrooke's Hosptital, Hills Road, Cambridge CB2 0QQ, UK
261. Divison of Human Cancer Genetics, Departments of Internal Medicine and Molecular Virology, Immunology and Medical Genetics, Comprehensive Cancer Center, The Ohio State University, Columbus, OH, USA
262. Cancer Epidemiology Unit, Nuffield Department of Population Health, University of Oxford, Oxford, UK
263. Hellenic Health Foundation, Athens, Greece
264. WHO Collaborating Center for Nutrition and Health, Unit of Nutritional Epidemiology and Nutrition in Public Health, Dept. of Hygiene, Epidemiology and Medical Statistics, University of Athens Medical School, Greece
265. Department of Medical Oncology, Beth Israel Deaconess Medical Center, Boston, MA, USA
266. Division of Gynecologic Oncology, Department of Obstetrics and Gynaecology, Radboud University Medical Centre, Nijmegen, The Netherlands
267. Department of Genetics, University Medical Center, Groningen University, Groningen, The Netherlands
268. Department of Medical Genetics, University Medical Center Utrecht, Utrecht, The Netherlands
269. Division of Gynecologic Oncology, Department of Obstetrics and Gynaecology and Leuven Cancer Institute, University Hospitals Leuven, Leuven, Belgium
270. Institute of Human Genetics, Campus Virchov Klinikum, Charite Berlin, Germany
271. Fundación Pública Galega de Medicina Xenómica, Servizo Galego de Saúde (SERGAS), Instituto de Investigaciones Sanitarias (IDIS), Santiago de Compostela, Spain
272. Grupo de Medicina Xenómica, Centro de Investigación Biomédica en Red de Enfermedades Raras (CIBERER), Universidade de Santiago de Compostela (USC), Santiago de Compostela, Spain
273. Vanderbilt Epidemiology Center, Vanderbilt Genetics Institute, Department of Obstetrics and Gynecology, Vanderbilt University Medical Center, Nashville, TN, USA
274. Oxford Regional Genetics Service, Churchill Hospital, Oxford, UK
275. Institute of Human Genetics, University Hospital, Leipzig, Germany
276. Department of Obstetrics and Gynecology, University of Ulm, Ulm, Germany
277. Population Health Department, QIMR Berghofer Medical Research Institute, 300 Herston Road, Herston, QLD 4006, Australia
278. Biostatistics and Computational Biology Branch, Division of Intramural Research, National Institute of Environmental Health Sciences, NIH, Research Triangle Park, NC, USA
279. Clinical Cancer Genetics, City of Hope, Duarte, CA, USA
280. Department of Data Management Science- Stanford University School of Medicine, Stanford, CA, USA
281. Department of Human Genetics and Department of Clinical Genetics, Leiden University Medical Center, Leiden, The Netherlands
282. Department of Epidemiology, University of California Irvine, Irvine, CA, USA

283. Women's College Research Institute, University of Toronto, Toronto, ON, Canada
284. Center for Genomic Medicine, Department of Biomedical Data Science, Geisel School of Medicine at Dartmouth, Williamson Translational Research Building, Room HB 7261, Lebanon, NH 03756, USA
285. Department of Public Health Sciences, The University of Virginia, Charlottesville, VA, USA
286. School of Women's and Children's Health, Lowy Cancer Research Centre, The University of New South Wales UNSW Sydney NSW 2052 AUSTRALIA
287. The Kinghorn Cancer Centre, Garvan Institute of Medical Research, 384 Victoria Street, Darlinghurst NSW 2010, Australia
288. Department of Molecular Medicine, University La Sapienza, c/o Policlinico Umberto I, viale Regina Elena 324, 00161 Rome, Italy
289. Community and Population Health Research Institute, Department of Biomedical Sciences, Cedars-Sinai Medical Center, Los Angeles, California, USA
290. Department of Preventive Medicine, College of Medicine, Seoul National University, 103 Daehak-ro, Jongno-gu, Seoul 110-799, Korea
291. Seoul National University Cancer Research Institute, 103 Daehak-ro, Jongno-gu, Seoul 110-799, Korea
292. Department of Biomedical Science, Graduate School, Seoul National University, 103 Daehak-ro, Jongno-gu, Seoul 110-799, Korea
293. Department of Public Health Sciences, College of Medicine, Medical University of South Carolina, Charleston, SC 29425
294. Department of Chronic Disease Epidemiology, Yale School of Public Health, New Haven, CT, USA
295. Department of Biomedical Sciences, Cedars-Sinai Medical Center, Los Angeles, California, USA

The Breast Cancer Association Consortium

K Michailidou[1,2], S Lindström[3], J Dennis[1], MK Bolla[1], Q Wang[1], R Keeman[4], S Behrens[5], H Anton-Culver[6], KJ Aronson[7], PL Auer[8,9], J Benitez[10,11], SE Bojesen[12,13,14], H Brenner[15,16,17], B Burwinkel[18,19], F Canzian[20], JE Castelao[21], JY Choi[22,23], CL Clarke[24], NBCS Collaborators, A Cox[25], K Czene[26], MB Daly[27], P Devilee[28,29], T Dörk[30], M Dwek[31], DM Eccles[32], PA Fasching[33,34], L Fritschi[35], M Gago-Dominguez[36,37], JA García-Sáenz[38], GG Giles[39,40], MS Goldberg[41,42], DE Goldgar[43], P Guénel[44], CA Haiman[45], U Hamann[46], M Hartman[47,48], A Hollestelle[49], MJ Hooning[49], R Hoppe[50,51], JL Hopper[40], M Iwasaki[52], A Jakubowska[53], EM John[54], R Kaaks[5], D Kang[22,23,55], VN Kristensen[56,57], A Kwong[58,59,60], D Lambrechts[61,62], A Lindblom[63], J Lubinski[53], C Luccarini[64], A Mannermaa[65,66,67], S Margolin[68], K Matsuo[69,70], U Menon[71], K Muir[72,73], SL Neuhausen[74], H Nevanlinna[75], OI Olopade[76], H Jernström[77], SK Park[22,23,55], P Peterlongo[78], J Peto[79], KA Phillips[40,80,81,82], D Plaseska-Karanfilska[83], R Prentice[8], P Radice[84], G Rennert[85], A Romero[86], E Saloustros[87], DP Sandler[88], EJ Sawyer[89], RK Schmutzler[90,91,92], CY Shen[93,94], XO Shu[95], MC Southey[39,96,97], JJ Spinelli[98,99], J Stone[40,100], A Swerdlow[101,102], R Tamimi[103,104,105], JA Taylor[88,106], LR Teras[107], MB Terry[108], C Vachon[109], CR Weinberg[110], C Wendt[111], R Winqvist[112,113], A Wolk[114], AH Wu[45], W Zheng[95], E Ziv[115], ABCTB Investigators*; kConFab/AOCS Investigators, AC Antoniou[1], IL

Andrulis[116,117], FJ Couch[118], PDP Pharoah[1,64], J Chang-Claude[5,119], P Hall[26,68], DJ Hunter[104,105], RL Milne[39,40], M García-Closas[120], MK Schmidt[4,121], SJ Chanock[120], AM Dunning[64], G Chenevix-Trench[122], J Simard[123], P Kraft[104,105], DF Easton[1,64]

1 Centre for Cancer Genetic Epidemiology, Department of Public Health and Primary Care, University of Cambridge, Cambridge, UK
2 Department of Electron Microscopy/Molecular Pathology, The Cyprus Institute of Neurology and Genetics, Nicosia, Cyprus
3 Department of Epidemiology, University of Washington School of Public Health, Seattle, WA, USA
4 Division of Molecular Pathology, The Netherlands Cancer Institute - Antoni van Leeuwenhoek Hospital, Amsterdam, The Netherlands
5 Division of Cancer Epidemiology, German Cancer Research Center (DKFZ), Heidelberg, Germany
6 Department of Epidemiology, University of California Irvine, Irvine, CA, USA
7 Department of Public Health Sciences, and Cancer Research Institute, Queen's University, Kingston, ON, Canada
8 Cancer Prevention Program, Fred Hutchinson Cancer Research Center, Seattle, WA, USA
9 Zilber School of Public Health, University of Wisconsin-Milwaukee, Milwaukee, WI, USA
10 Human Cancer Genetics Program, Spanish National Cancer Research Centre, Madrid, Spain
11 Centro de Investigación en Red de Enfermedades Raras (CIBERER), Valencia, Spain
12 Copenhagen General Population Study, Herlev and Gentofte Hospital, Copenhagen University Hospital, Herlev, Denmark
13 Department of Clinical Biochemistry, Herlev and Gentofte Hospital, Copenhagen University Hospital, Herlev, Denmark
14 Faculty of Health and Medical Sciences, University of Copenhagen, Copenhagen, Denmark
15 German Cancer Consortium (DKTK), German Cancer Research Center (DKFZ), Heidelberg, Germany
16 Division of Clinical Epidemiology and Aging Research, German Cancer Research Center (DKFZ), Heidelberg, Germany
17 Division of Preventive Oncology, German Cancer Research Center (DKFZ) and National Center for Tumor Diseases (NCT), Heidelberg, Germany
18 Molecular Epidemiology Group, C080, German Cancer Research Center (DKFZ), Heidelberg, Germany
19 Molecular Biology of Breast Cancer, University Womens Clinic Heidelberg, University of Heidelberg, Heidelberg, Germany
20 Genomic Epidemiology Group, German Cancer Research Center (DKFZ), Heidelberg, Germany
21 Oncology and Genetics Unit, Instituto de Investigacion Biomedica (IBI) Orense-PontevedraVigo, Xerencia de Xestion Integrada de Vigo-SERGAS, Vigo, Spain
22 Department of Biomedical Sciences, Seoul National University Graduate School, Seoul, Korea

23 Cancer Research Institute, Seoul National University, Seoul, Korea
24 Westmead Institute for Medical Research, University of Sydney, Sydney, Australia
25 Sheffield Institute for Nucleic Acids (SInFoNiA), Department of Oncology and Metabolism, University of Sheffield, Sheffield, UK
26 Department of Medical Epidemiology and Biostatistics, Karolinska Institutet, Stockholm, Sweden
27 Department of Clinical Genetics, Fox Chase Cancer Center, Philadelphia, PA, USA
28 Department of Pathology, Leiden University Medical Center, Leiden, The Netherlands
29 Department of Human Genetics, Leiden University Medical Center, Leiden, The Netherlands
30 Gynaecology Research Unit, Hannover Medical School, Hannover, Germany
31 School of Life Sciences, University of Westminster, London, UK
32 Faculty of Medicine, University of Southampton, Southampton, UK
33 Institute of Human Genetics, University Hospital Erlangen, Friedrich-Alexander University Erlangen-Nuremberg, Comprehensive Cancer Center Erlangen-EMN, Erlangen, Germany
34 David Geffen School of Medicine, Department of Medicine Division of Hematology and Oncology, University of California at Los Angeles, Los Angeles, CA, USA
35 School of Public Health, Curtin University, Perth, Australia
36 Genomic Medicine Group, Galician Foundation of Genomic Medicine, Instituto de Investigación Sanitaria de Santiago de Compostela (IDIS), Complejo Hospitalario Universitario de Santiago, SERGAS, Santiago de Compostela, Spain
37 Moores Cancer Center, University of California San Diego, La Jolla, CA, USA
38 Medical Oncology Department, Hospital Clínico San Carlos, IdISSC (Centro Investigacion Biomedica en Red), CIBERONC (Instituto de Investigación Sanitaria San Carlos), Madrid, Spain
39 Cancer Epidemiology Division, Cancer Council Victoria, Melbourne, Victoria, Australia
40 Centre for Epidemiology and Biostatistics, Melbourne School of Population and Global Health, The University of Melbourne, Melbourne, Australia
41 Department of Medicine, McGill University, Montréal, QC, Canada
42 Division of Clinical Epidemiology, Royal Victoria Hospital, McGill University, Montréal, QC, Canada
43 Department of Dermatology, Huntsman Cancer Institute, University of Utah School of Medicine, Salt Lake City, UT, USA
44 Cancer & Environment Group, Center for Research in Epidemiology and Population Health (CESP), INSERM, University Paris-Sud, University Paris-Saclay, Villejuif, France
45 Department of Preventive Medicine, Keck School of Medicine, University of Southern California, Los Angeles, CA, USA
46 Molecular Genetics of Breast Cancer, German Cancer Research Center (DKFZ), Heidelberg, Germany

47 Saw Swee Hock School of Public Health, National University of Singapore, Singapore, Singapore
48 Department of Surgery, National University Health System, Singapore, Singapore
49 Department of Medical Oncology, Family Cancer Clinic, Erasmus MC Cancer Institute, Rotterdam, The Netherlands
50 Dr. Margarete Fischer-Bosch-Institute of Clinical Pharmacology, Stuttgart, Germany
51 University of Tübingen, Tübingen, Germany
52 Division of Epidemiology, Center for Public Health Sciences, National Cancer Center, Tokyo, Japan
53 Department of Genetics and Pathology, Pomeranian Medical University, Szczecin, Poland
54 Department of Medicine, Division of Oncology, Stanford Cancer Institute, Stanford University School of Medicine, Stanford, CA, USA
55 Department of Preventive Medicine, Seoul National University College of Medicine, Seoul, Korea
56 Department of Cancer Genetics, Institute for Cancer Research, Oslo University Hospital Radiumhospitalet, Oslo, Norway
57 Institute of Clinical Medicine, Faculty of Medicine, University of Oslo, Oslo, Norway
58 Hong Kong Hereditary Breast Cancer Family Registry, Happy Valley, Hong Kong
59 Department of Surgery, The University of Hong Kong, Pok Fu Lam, Hong Kong
60 Department of Surgery, Hong Kong Sanatorium and Hospital, Happy Valley, Hong Kong
61 VIB Center for Cancer Biology, Leuven, Belgium
62 Laboratory for Translational Genetics, Department of Human Genetics, University of Leuven, Leuven, Belgium
63 Department of Molecular Medicine and Surgery, Karolinska Institutet, Stockholm, Sweden
64 Centre for Cancer Genetic Epidemiology, Department of Oncology, University of Cambridge, Cambridge, UK
65 Translational Cancer Research Area, University of Eastern Finland, Kuopio, Finland
66 Institute of Clinical Medicine, Pathology and Forensic Medicine, University of Eastern Finland, Kuopio, Finland
67 Imaging Center, Department of Clinical Pathology, Kuopio University Hospital, Kuopio, Finland
68 Department of Oncology, Södersjukhuset, Stockholm, Sweden
69 Division of Cancer Epidemiology and Prevention, Aichi Cancer Center Research Institute, Nagoya, Japan
70 Division of Cancer Epidemiology, Nagoya University Graduate School of Medicine, Nagoya, Japan
71 MRC Clinical Trials Unit at UCL, Institute of Clinical Trials & Methodology, University College London, London, UK
72 Division of Population Health, Health Services Research and Primary Care, School of Health Sciences, Faculty of Biology, Medicine and Health, The University

of Manchester, Manchester, UK
73 Division of Health Sciences, Warwick Medical School, University of Warwick, Coventry, UK
74 Department of Population Sciences, Beckman Research Institute of City of Hope, Duarte, CA, USA
75 Department of Obstetrics and Gynecology, Helsinki University Hospital, University of Helsinki, Helsinki, Finland
76 Center for Clinical Cancer Genetics, The University of Chicago, Chicago, IL, USA
78. Oncology, Clinical Sciences in Lund, Lund University, 221 85 Lund, Sweden
77 Oncology, Clinical Sciences in Lund, Lund University, 221 85 Lund, Sweden
78 Genome Diagnostics Program, IFOM - the FIRC (Italian Foundation for Cancer Research) Institute of Molecular Oncology, Milan, Italy
79 Department of Non-Communicable Disease Epidemiology, London School of Hygiene and Tropical Medicine, London, UK
80 Peter MacCallum Cancer Center, Melbourne, Australia
81 Sir Peter MacCallum Department of Oncology, The University of Melbourne, Melbourne, Australia
82 Department of Medicine, St Vincent’s Hospital, The University of Melbourne, Fitzroy, Australia
83 Research Centre for Genetic Engineering and Biotechnology "Georgi D. Efremov", MASA, Skopje, Republic of North Macedonia
84 Unit of ”Predictive Medicine: Molecular Bases of Genetic Risk”, Department of Experimental Oncology, Fondazione IRCCS Istituto Nazionale dei Tumori, Milan, Italy
85 Clalit National Cancer Control Center, Carmel Medical Center and Technion Faculty of Medicine, Haifa, Israel
86 Medical Oncology Department, Hospital Universitario Puerta de Hierro, Madrid, Spain
87 Department of Oncology, University Hospital of Larissa, Larissa, Greece
88 Epidemiology Branch, National Institute of Environmental Health Sciences, NIH, Research Triangle Park, NC, USA
89 Research Oncology, Guy’s Hospital, King's College London, London, UK
90 Center for Familial Breast and Ovarian Cancer, Faculty of Medicine and University Hospital Cologne, University of Cologne, Cologne, Germany
91 Center for Integrated Oncology (CIO), Faculty of Medicine and University Hospital Cologne, University of Cologne, Cologne, Germany
92 Center for Molecular Medicine Cologne (CMMC), Faculty of Medicine and University Hospital Cologne, University of Cologne, Cologne, Germany
93 Taiwan Biobank, Institute of Biomedical Sciences, Academia Sinica, Taipei, Taiwan
94 School of Public Health, China Medical University, Taichung, Taiwan
95 Division of Epidemiology, Department of Medicine, Vanderbilt Epidemiology Center, Vanderbilt-Ingram Cancer Center, Vanderbilt University School of Medicine, Nashville, TN, USA
96 Precision Medicine, School of Clinical Sciences at Monash Health, Monash University, Clayton, Victoria, Australia

97 Department of Clinical Pathology, The University of Melbourne, Melbourne, Victoria, Australia
98 Population Oncology, BC Cancer, Vancouver, BC, Canada
99 School of Population and Public Health, University of British Columbia, Vancouver, BC, Canada
100 The Curtin UWA Centre for Genetic Origins of Health and Disease, Curtin University and University of Western Australia, Perth, Australia
101 Division of Genetics and Epidemiology, The Institute of Cancer Research, London, UK
102 Division of Breast Cancer Research, The Institute of Cancer Research, London, UK
103 Channing Division of Network Medicine, Department of Medicine, Brigham and Women's Hospital, Harvard Medical School, Boston, MA, USA
104 Department of Epidemiology, Harvard T.H. Chan School of Public Health, Boston, MA, USA
105 Program in Genetic Epidemiology and Statistical Genetics, Harvard T.H. Chan School of Public Health, Boston, MA, USA
106 Epigenetic and Stem Cell Biology Laboratory, National Institute of Environmental Health Sciences, NIH, Research Triangle Park, NC, USA
107 Department of Population Science American Cancer Society Atlanta, GA, USA
108 Department of Epidemiology, Mailman School of Public Health, Columbia University, New York, NY, USA
109 Department of Health Sciences Research, Division of Epidemiology, Mayo Clinic, Rochester, MN, USA
110 Biostatistics and Computational Biology Branch, National Institute of Environmental Health Sciences, NIH, Research Triangle Park, NC, USA
111 Department of Clinical Science and Education, Södersjukhuset, Karolinska Institutet, Stockholm, Sweden
112 Laboratory of Cancer Genetics and Tumor Biology, Cancer and Translational Medicine Research Unit, Biocenter Oulu, University of Oulu, Oulu, Finland
113 Laboratory of Cancer Genetics and Tumor Biology, Northern Finland Laboratory Centre Oulu, Oulu, Finland
114 Institute of Environmental Medicine, Karolinska Institutet, Stockholm, Sweden
115 Department of Medicine, Institute for Human Genetics, UCSF Helen Diller Family Comprehensive Cancer Center, University of California San Francisco, San Francisco, CA, USA
116 Fred A. Litwin Center for Cancer Genetics, Lunenfeld-Tanenbaum Research Institute of Mount Sinai Hospital, Toronto, ON, Canada
117 Department of Molecular Genetics, University of Toronto, Toronto, ON, Canada
118 Department of Laboratory Medicine and Pathology, Mayo Clinic, Rochester, MN, USA
119 Cancer Epidemiology Group, University Cancer Center Hamburg (UCCH), University Medical Center Hamburg-Eppendorf, Hamburg, Germany
120 Division of Cancer Epidemiology and Genetics, National Cancer Institute, National Institutes of Health, Department of Health and Human Services, Bethesda, MD, USA

121 Division of Psychosocial Research and Epidemiology, The Netherlands Cancer Institute - Antoni van Leeuwenhoek hospital, Amsterdam, The Netherlands
122 Department of Genetics and Computational Biology, QIMR Berghofer Medical Research Institute, Brisbane, Australia
123 Genomics Center, Centre Hospitalier Universitaire de Québec – Université Laval Research Center, Québec City, QC, Canada

***ABCTB Investigators:**
Christine Clarke (Westmead Institute for Medical Research, University of Sydney, NSW, Australia); Rosemary Balleine (Pathology West ICPMR, Westmead, NSW, Australia); Robert Baxter (Kolling Institute of Medical Research, University of Sydney, Royal North Shore Hospital, NSW, Australia); Stephen Braye (Pathology North, John Hunter Hospital, Newcastle, NSW, 2305, Australia); Jane Carpenter (Westmead Institute for Medical Research, University of Sydney); Jane Dahlstrom (Department of Anatomical Pathology, ACT Pathology, Canberra Hospital, ACT, Australia; ANU Medical School, Australian National University, ACT, Australia); John Forbes (Department of Surgical Oncology, Calvary Mater Newcastle Hospital, Australian New Zealand Breast Cancer Trials Group, and School of Medicine and Public Health, University of Newcastle, NSW, Australia); C Soon Lee (School of Science and Health, The University of Western Sydney, Sydney, Australia); Deborah Marsh (Hormones and Cancer Group, Kolling Institute of Medical Research, Royal North Shore Hospital, University of Sydney, NSW, Australia); Adrienne Morey (SydPath St Vincent's Hospital, Sydney, NSW, Australia); Nirmala Pathmanathan (Department of Tissue Pathology and Diagnostic Oncology, Pathology West; Westmead Breast Cancer Institute, Westmead Hospital, NSW, Australia); Mythily Sachchithananthan (Australian Breast Cancer Tissue Bank, Westmead Institute for Medical Research, University of Sydney, Sydney, New South Wales, Australia); Rodney Scott (Centre for Information Based Medicine, Hunter Medical Research Institute, NSW, 2305, Australia; Priority Research Centre for Cancer, School of Biomedical Sciences and Pharmacy, Faculty of Health, University of Newcastle, NSW, Australia); Peter Simpson (The University of Queensland: UQ Centre for Clinical Research and School of Medicine, QLD, Australia); Allan Spigelman (Hereditary Cancer Clinic, St Vincent's Hospital, The Kinghorn Cancer Centre, Sydney, New South Wales, 2010, Australia); Nicholas Wilcken (Crown Princess Mary Cancer Centre, Westmead Hospital, Westmead, Australia; Sydney Medical School - Westmead, University of Sydney, NSW, Australia); Desmond Yip (Department of Medical Oncology, The Canberra Hospital, ACT, Australia; ANU Medical School, Australian National University, ACT, Australia); Nikolajs Zeps (St John of God Perth Northern Hospitals, Perth, WA, Australia).

Study-specific text & acknowledgements

ALSPAC

Funding: The UK Medical Research Council and Wellcome (Grant ref: 217065/Z/19/Z) and the University of Bristol provide core support for ALSPAC. A comprehensive list of grants funding is available on the ALSPAC website: (http://www.bristol.ac.uk/alspac/external/documents/grant-acknowledgements.pdf).
Acknowledgements: We are extremely grateful to all the families who took part in this study, the midwives for their help in recruiting them, and the whole ALSPAC team, which includes interviewers, computer and laboratory technicians, clerical workers, research scientists, volunteers, managers, receptionists and nurses. None of the funders influenced the study design, analyses or interpretation of the findings. This publication is the work of the authors and JRBP will serve as guarantor for the contents of this paper. The views expressed in this paper are those of the authors and not necessarily any of the funders.

The Atherosclerosis Risk in Communities study

The Atherosclerosis Risk in Communities study has been funded in whole or in part with Federal funds from the National Heart, Lung, and Blood Institute, National Institutes of Health, Department of Health and Human Services, under Contract nos. (75N92022D00001, 75N92022D00002, 75N92022D00003, 75N92022D00004, 75N92022D00005). The authors thank the staff and participants of the ARIC study for their important contributions.
GWAS was supported by R01HL087641, and R01HL086694; National Human Genome Research Institute contract U01HG004402; and National Institutes of Health contract HHSN268200625226C. Infrastructure was partly supported by Grant Number UL1RR025005, a component of the National Institutes of Health and NIH Roadmap for Medical Research. Funding support for “Building on GWAS for NHLBI-diseases: the U.S. CHARGE consortium” was provided by the NIH through the American Recovery and Reinvestment Act of 2009 (ARRA) (5RC2HL102419).

INGI-Carlantino

We are very grateful to the municipal administrators for their collaboration on the project and for logistic support. We would like to thank all participants in the study. The study was funded by Regione FVG (L.26.2008).

CILENTO

We address special thanks to the populations of Campora, Gioi, and Cardile for their participation in the study. This work was supported by grants from the Italian Ministry of Universities (PON03PE_00060_7, IDF SHARID ARS01_01270), the Assessorato Ricerca Regione Campania (POR CAMPANIA 2000/2006 MISURA 3.16).

CROATIA-Korcula

We would like to acknowledge the contributions of the recruitment team in Korcula, the administrative teams in Croatia and Edinburgh and the people of Korcula. The SNP genotyping for the KORCULA cohort was performed in Helmholtz Zentrum München, Neuherberg, Germany. Funding provided by Medical Research Council UK, the Ministry of Science, Education and Sport in the Republic of Croatia (number 216-1080315-0302) and Croatian Science Foundation (grant 8875).

CROATIA-Vis
We would like to acknowledge the invaluable contributions of the recruitment teams in Vis (including those from the Institute of Anthropological Research in Zagreb and the Croatian Centre for Global Health at the University of Split), the administrative teams in Croatia and Edinburgh and the people of Vis. The CROATIA-Vis study was supported through grants from the Medical Research Council UK and Ministry of Science, Education and Sport of the Republic of Croatia (number 108-1080315-0302) and the European Union framework program 6 EUROSPAN project (contract no. LSHG-CT-2006-018947). We would like to acknowledge the staff of several institutions in Croatia that supported the field work, including but not limited to The University of Split and Zagreb Medical Schools, the Institute for Antroplogical Research in Zagreb and the Croatian Institute for Public Health. We would like to acknowledge the invaluable contributions of the recruitment team in Korcula, the administrative teams in Croatia and Edinburgh and the participants. The SNP genotyping was performed in Helmholtz Zentrum München, Neuherberg, Germany. The study was funded by the Medical Research Council UK, The Croatian Ministry of Science, Education and Sports (grant 216-1080315-0302), the European Union framework program 6 EUROSPAN project (contract no. LSHG-CT-2006-018947), the Croatian Science Foundation (grant 8875), the Croatian National Center of Research Excellence in Personalized Healthcare (grant number KK.01.1.1.01.0010) and the Center of Competence in Molecular Diagnostics (KK.01.2.2.03.0006).

EGCUT
This study was supported by EU H2020 grants 692145, 676550, 654248, Estonian Research Council Grant IUT20-60, NIASC, and EU through the European Regional Development Fund (Project No. 2014- 2020.4.01.15-0012 GENTRANSMED).

EPIC
The coordination of EPIC is financially supported by the European Commission (DG-SANCO) and the International Agency for Research on Cancer. The national cohorts (that recruited male participants) are supported by Danish Cancer Society (Denmark); German Cancer Aid, German Cancer Research Center (DKFZ), Federal Ministry of Education and Research (BMBF), Deutsche Krebshilfe, Deutsches Krebsforschungszentrum and Federal Ministry of Education and Research (Germany); the Hellenic Health Foundation (Greece); Associazione Italiana per la Ricerca sul Cancro-AIRC-Italy and National Research Council (Italy); Dutch Ministry of Public Health, Welfare and Sports (VWS), Netherlands Cancer Registry (NKR), LK Research Funds, Dutch Prevention Funds, Dutch ZON (Zorg Onderzoek Nederland), World Cancer Research Fund (WCRF), Statistics Netherlands (The Netherlands); Health Research Fund (FIS), PI13/00061 to Granada, PI13/01162 to EPICMurcia, Regional Governments of Andalucía, Asturias, Basque Country, Murcia and Navarra, ISCIII RETIC (RD06/0020) (Spain); Swedish Cancer Society, Swedish Research Council and County Councils of Skåne and Västerbotten (Sweden); Cancer Research UK (14136 to EPIC-Norfolk; C570/A16491 and C8221/A19170 to EPICOxford), Medical Research Council (1000143 to EPIC-Norfolk, MR/M012190/1 to EPICOxford) (United Kingdom). The EPIC Norfolk Study is funded by Cancer Research United Kingdom and the Medical Research Council. This work was supported by the Medical Research Council [U106179472; MC_U106179472; U106179471; MC_U106179471]. The authors acknowledge the support of all EPICNorfolk staff and participants.

INGI-FVG
We are very grateful to the municipal administrators for their collaboration on the project and for logistic support. We would like to thank all participants in the study. The study was funded by Regione FVG (L.26.2008).

Generation Scotland
Generation Scotland received core support from the Chief Scientist Office of the Scottish Government Health Directorates [CZD/16/6] and the Scottish Funding Council [HR03006] and is currently supported by the Wellcome Trust [216767/Z/19/Z]. Genotyping of the GS:SFHS samples was carried out by the Genetics Core Laboratory at the Edinburgh Clinical Research Facility, University of Edinburgh, Scotland and was funded by the Medical Research Council UK and the Wellcome Trust (Wellcome Trust Strategic Award "STratifying Resilience and Depression Longitudinally" (STRADL) Reference 104036/Z/14/Z).

HBCS
We acknowledge support from grants from the Academy of Finland, the Finnish Diabetes Research Society, Folkhälsan Research Foundation, Novo Nordisk Foundation, Finska Läkaresällskapet, Signe and Ane Gyllenberg Foundation, University of Helsinki, European Science Foundation (EUROSTRESS), Ministry of Education, Ahokas Foundation, Emil Aaltonen Foundation.

InCHIANTI
The InCHIANTI study baseline (1998-2000) was supported as a "targeted project" (ICS110.1/RF97.71) by the Italian Ministry of Health and in part by the U.S. National Institute on Aging (Contracts: 263 MD 9164 and 263 MD 821336).

InterAct
We thank all EPIC participants and staff for their contribution to the study. We thank staff from the Technical, Field Epidemiology and Data Functional Group Teams of the MRC Epidemiology Unit in Cambridge, UK, for carrying out sample preparation, DNA provision and quality control, genotyping and data- handling work. The EPIC-InterAct study received funding from the European Union (Integrated Project LSHM- CT-2006-037197 in the Framework Programme 6 of the European Community).

KORA
KORA F3 and F4: The KORA study was initiated and financed by the Helmholtz Zentrum München – German Research Center for Environmental Health, which is funded by the German Federal Ministry of Education and Research (BMBF) and by the State of Bavaria. Data collection in the KORA study is done in cooperation with the University Hospital of Augsburg. We thank all participants for their long-term commitment to the KORA study, the staff for data collection and research data management and the members of the KORA Study Group (https://www.helmholtz-munich.de/en/epi/cohort/kora) who are responsible for the design and conduct of the study.

Lifelines
The Lifelines Biobank initiative has been made possible by funding from the Dutch Ministry of Health, Welfare and Sport, the Dutch Ministry of Economic Affairs, the

University Medical Center Groningen (UMCG the Netherlands), University of Groningen and the Northern Provinces of the Netherlands. The generation and management of GWAS genotype data for the Lifelines Cohort Study is supported by the UMCG Genetics Lifelines Initiative (UGLI). UGLI is partly supported by a Spinoza Grant from NWO, awarded to Cisca Wijmenga. The authors wish to acknowledge the services of the Lifelines Cohort Study, the contributing research centers delivering data to Lifelines, and all the study participants. The LifeLines Cohort Study, and generation and management of GWAS genotype data for the LifeLines Cohort Study is supported by the Netherlands Organization of Scientific Research NWO (grant 175.010.2007.006), the Economic Structure Enhancing Fund (FES) of the Dutch government, the Ministry of Economic Affairs, the Ministry of Education, Culture and Science, the Ministry for Health, Welfare and Sports, the Northern Netherlands Collaboration of Provinces (SNN), the Province of Groningen, University Medical Center Groningen, the University of Groningen, Dutch Kidney Foundation and Dutch Diabetes Research Foundation. The authors wish to acknowledge the services of the Lifelines Cohort Study, the contributing research centers delivering data to Lifelines, and all the study participants.

NEO
The authors of the NEO study thank all individuals who participated in the Netherlands Epidemiology in Obesity study, all participating general practitioners for inviting eligible participants and all research nurses for collection of the data. We thank the NEO study group, Petra Noordijk, Pat van Beelen and Ingeborg de Jonge for the coordination, lab and data management of the NEO study. The genotyping in the NEO study was supported by the Centre National de Génotypage (Paris, France), headed by Jean-Francois Deleuze. The NEO study is supported by the participating Departments, the Division and the Board of Directors of the Leiden University Medical Center, and by the Leiden University, Research Profile Area Vascular and Regenerative Medicine.

NHS
We would like to thank the participants and staff of the NHS and NHSII for their valuable contributions. The authors assume full responsibility for analyses and interpretation of these data. The NHS/NHSII studies thank the following state cancer registries for their help: AL, AZ, AR, CA, CO, CT, DE, FL, GA, ID, IL, IN, IA, KY, LA, ME, MD, MA, MI, NE, NH, NJ, NY, NC, ND, OH, OK, OR, PA, RI, SC, TN, TX, VA, WA, and WY. The NHS GWAS were supported by grants from the National Institutes of Health [NCI (CA40356, CA087969, CA055075, CA98233, U01 CA137088, R01 CA059045, R01 CA137178, R01 CA082838, R01 CA131332, R01 CA49449), NIDDK (DK058845, DK070756), NHGRI (HG004399, HG004728), NHLBI (HL35464), NIAMS (R01 AR056291)], UM1 CA186107, P01 CA87969.

The Netherlands Twin Register
We are grateful to all participants from the Netherlands Twin Register. We acknowledge funding the Netherlands Organization for Scientific Research (NWO) and The Netherlands Organization for Health Research and Development (ZonMW) grants, 904-61-193,480-04-004, 400-05-717, Addiction-31160008, 911-09-032, Biobanking and Biomolecular Resources Research Infrastructure (BBMRI –NL, 184.021.007); NWO 480-15-001/674: Netherlands Twin Registry Repository. European Research Council (ERC-230374 and ERC-284167); Rutgers University Cell and DNA Repository (NIMH U24 MH068457-06), the Avera Institute, Sioux Falls,

South Dakota (USA) and the National Institutes of Health (NIH R01 HD042157-01A1). Part of the genotyping was funded by the Genetic Association Information Network (GAIN) of the Foundation for the National Institutes of Health and Grand Opportunity grants 1RC2 MH089951). We acknowledge support from VU Amsterdam and the Amsterdam Reproduction and Development (AR&D) research institute.

NFBC1966
We thank all cohort members and researchers who participated in the 31y and 46y NFBC1966 study. We also wish to acknowledge the work of the NFBC project centre. NFBC1966 received financial support from University of Oulu Grant no. 65354, Oulu University Hospital Grant no. 2/97, 8/97, Ministry of Health and Social Affairs Grant no. 23/251/97, 160/97, 190/97, National Institute for Health and Welfare, Helsinki Grant no. 54121, Regional Institute of Occupational Health, Oulu, Finland Grant no. 50621, 54231, University of Oulu Grant no. 24000692, Oulu University Hospital Grant no. 24301140, ERDF European Regional Development Fund Grant no. 539/2010 A31592, Academy of Finland grant numbers 24300796, 24302031, 285547 (EGEA) (MRJ); the Medical Research Council (MRC) UK (grant number G0601653) (MRJ); Medical Research Council Biotechnology and Biological Sciences Research Council PREcisE (Nutrition & Epigenome, The Joint Programming Initiative a Healthy Diet for a Healthy Life (JPI HDHL/EU-H2020)) (MRJ); Yrjö Jahnsson Foundation (SP), Päivikki and Sakari Sohlberg Foundation sr (SP); the European Union's Horizon 2020 programmes, iHealth-T2D (grant number 643774) and EDCMET (grant number 825762) (SP).

The Orkney Complex Disease Study
The Orkney Complex Disease Study (ORCADES) was supported by the Chief Scientist Office of the Scottish Government (CZB/4/276, CZB/4/710), a Royal Society URF to J.F.W., the MRC Human Genetics Unit quinquennial programme “QTL in Health and Disease”, Arthritis Research UK and the European Union framework program 6 EUROSPAN project (contract no. LSHG-CT-2006-018947). DNA extractions were performed at the Edinburgh Clinical Research Facility, University of Edinburgh. We would like to acknowledge the invaluable contributions of the research nurses in Orkney, the administrative team in Edinburgh and the people of Orkney. For the purpose of open access, the author has applied a Creative Commons Attribution (CC BY) licence to any Author Accepted Manuscript version arising from this submission.

QIMR
We thank the participants and their families for contributing to this research. We also thank A Henders, B Usher, E Souzeau, A Kuot, A McMellon, MJ Wright, MJ Campbell, A Caracella, L Bowdler, S Smith, B Haddon, A Conciatore, D Smyth, H Beeby, O Zheng and B Chapman for their input into project management, databases, phenotype collection, and sample collection, processing and genotyping. The QIMR cohort was supported by National Institutes of Health (NIH) Grants AA07535, AA07728, AA13320, AA13321, AA14041, AA11998, AA17688, DA012854, DA019951, AA010249, AA013320, AA013321, AA011998, AA017688, and DA027995; by Grants from the Australian National Health and Medical Research Council (NHMRC) (241944, 339462, 389927, 389875, 389891, 389892, 389938, 442915, 442981, 496739, 552485, 552498 and 1075175); by Grants from the Australian Research Council (ARC) (A7960034, A79906588, A79801419, DP0770096, DP0212016, and DP0343921).

SardiNIA
This research was supported by the Intramural Research Program of the National Institute on Aging, National Institutes of Health (NIH) (contract 75N95021C00012-SardiNIA5).

SHIP
SHIP is part of the Community Medicine Research Network of the University Medicine Greifswald, which is supported by the German Federal State of Mecklenburg- West Pomerania.

TwinGene
TwinGene is part of the Swedish Twin Registry which is managed by Karolinska Institutet and receives funding through the Swedish Research Council under the grant no 2017-00641 and 2021-00180. Previous funding for data collection and build-up of TwinGene has been received through The Ministry for Higher Education; The Swedish Research Council (M-2005-1112); GenomEUtwin (EU/QLRT-2001-01254; QLG2-CT-2002-01254); NIH DK U01-066134; The Swedish Foundation for Strategic Research (SSF); Heart and Lung foundation no. 20070481.

TWINS UK
TwinsUK is funded by the Wellcome Trust, Medical Research Council, Versus Arthritis, European Union Horizon 2020, Chronic Disease Research Foundation (CDRF), Zoe Ltd, the National Institute for Health and Care Research (NIHR) Clinical Research Network (CRN) and Biomedical Research Centre based at Guy’s and St Thomas’ NHS Foundation Trust in partnership with King’s College London.

VB
We thank all the participants to the project, the San Raffaele Hospital MDs who contributed to clinical data collection, prof. Clara Camaschella who coordinated the data collection, Corrado Masciullo and Massimiliano Cocca for the database informatics. The research was supported by funds from Compagnia di San Paolo, Torino, Italy; Fondazione Cariplo, Italy; Telethon Italy; Ministry of Health, Ricerca Finalizzata 2008 and 2011-2012 and Public Health Genomics Project 2010.

AGES
This study has been funded by NIH contracts N01-AG-1-2100 and 271201200022C, the NIA Intramural Research Program, Hjartavernd (the Icelandic Heart Association), and the Althingi (the Icelandic Parliament). The study is approved by the Icelandic National Bioethics Committee, VSN: 00-063. The researchers are indebted to the participants for their willingness to participate in the study.

FHS
The Framingham Heart Study phenotype-genotype analyses were supported by NIA R01AG29451 JMM, KLL). The Framingham Heart Study of the National Heart Lung and Blood Institute of the National Institutes of Health and Boston University School of Medicine was supported by the National Heart, Lung and Blood Institute's Framingham Heart Study Contract No. N01-HC-25195 and its contract with Affymetrix, Inc for genotyping services (Contract No. N02-HL-6-4278). Analyses reflect intellectual input and resource development from the Framingham Heart Study

investigators participating in the SNP Health Association Resource (SHARe) project. A portion of this research was conducted using the Linux Cluster for Genetic Analysis (LinGA-II) funded by the Robert Dawson Evans Endowment of the Department of Medicine at Boston University School of Medicine and Boston Medical Center.

Rotterdam
The generation and management of GWAS genotype data for the Rotterdam Study are supported by the Netherlands Organisation of Scientific Research NWO Investments (nr. 175.010.2005.011, 911- 03-012). This study is funded by the Research Institute for Diseases in the Elderly (014-93-015; RIDE2), the Netherlands Genomics Initiative (NGI)/Netherlands Organisation for Scientific Research (NWO) project nr. 050-060-810, and funding from the European Commision (HEALTH-F2-2008-201865, GEFOS; HEALTHF2-2008-35627, TREAT-OA) . The Rotterdam Study is funded by Erasmus Medical Center and Erasmus University, Rotterdam, Netherlands Organization for the Health Research and Development (ZonMw), the Research Institute for Diseases in the Elderly (RIDE), the Ministry of Education, Culture and Science, the Ministry for Health, Welfare and Sports, the European Commission (DG XII), and the Municipality of Rotterdam. We thank Pascal Arp, Mila Jhamai, Dr Michael Moorhouse, Marijn Verkerk, and Sander Bervoets for their help in creating the GWAS database. The authors are grateful to the study participants, the staff from the Rotterdam Study and the participating general practioners and pharmacists. We would like to thank Dr. Tobias A. Knoch, Anis Abuseiris, Karol Estrada, and Rob de Graaf as well as their institutions Biophysical Genomics, Erasmus MC Rotterdam, The Netherlands, and especially the national German MediGRID and Services@MediGRID part of the German D-Grid, both funded by the German Bundesministerium fuer Forschung und Technology under grants #01 AK 803 A-H and # 01 IG 07015 G for access to their grid resources.

The Women's genome Health study
The WGHS is supported by the National Heart, Lung, and Blood Institute (HL043851 and HL080467) and the National Cancer Institute (CA047988 and UM1CA182913), with funding for genotyping provided by Amgen.

BMDCS
We appreciate the dedication of the BMDCS study participants and their families, and the support of Dr. Karen Winer, Scientific Director of this effort. This study was funded by R01 HD58886, R01 HD100406, the Eunice Kennedy Shriver National Institute of Child Health and Human Development (NICHD) contracts (N01-HD-1-3228, -3329, -3330, -3331, -3332, -3333), and the CTSA program Grant 8 UL1 TR000077. S.F.A.G. is supported by the Daniel B. Burke Endowed Chair for Diabetes Research, R01 HD056465 and R01 HG010067.

CHOP
This research was financially supported by an Institute Development Award from the Children's Hospital of Philadelphia, a Research Development Award from the Cotswold Foundation, the Children´s Hospital of Philadelphia Endowed Chair in Genomic Research, the Daniel B. Burke Endowed Chair for Diabetes Research and NIH grant R01 HD056465. The authors thank the network of primary care clinicians and the patients and families for their contribution to this project and to clinical research facilitated by the Pediatric Research Consortium (PeRC) at The Children's Hospital of

Philadelphia. R. Chiavacci, E. Dabaghyan, A. (Hope) Thomas, K. Harden, A. Hill, C. Johnson-Honesty, C. Drummond, S. Harrison, F. Salley, C. Gibbons, K. Lilliston, C. Kim, E. Frackelton, F. Mentch, G. Otieno, K. Thomas, C. Hou, K. Thomas and M.L. Garris provided expert assistance with genotyping and/or data collection and management. The authors would also like to thank S. Kristinsson, L.A. Hermannsson and A. Krisbjörnsson of Raförninn ehf for extensive software design and contributions.

The Raine Study
Funding: The Raine Study was supported by the National Health and Medical Research Council of Australia [grant numbers 572613, 403981 and 1059711] and the Canadian Institutes of Health Research [grant number MOP-82893].
Acknowledgement: The authors are grateful to the Raine Study participants and their families, and to the Raine Study research staff for cohort coordination and data collection. The authors gratefully acknowledge the NH&MRC for their long term funding to the study over the last 30 years and also the following institutes for providing funding for Core Management of the Raine Study: The University of Western Australia (UWA) , Curtin University, the Raine Medical Research Foundation, the UWA Faculty of Medicine, Dentistry and Health Sciences, the Telethon Kids Institute, the Women's and Infant's Research Foundation (King Edward Memorial Hospital), Murdoch University, The University of Notre Dame (Australia), and Edith Cowan University. The authors gratefully acknowledge the assistance of the Western Australian DNA Bank (National Health and Medical Research Council of Australia National Enabling Facility). We would also like to acknowledge the Raine Study participants for their ongoing participation in the study, and the Raine Study Team for study co-ordination and data collection. This work was supported by resources provided by the Pawsey Supercomputing Centre with funding from the Australian Government and Government of Western Australia.

deCODE
We thank the study subjects for their valuable participation. All deCODE collaborators in this study are employees of deCODE Genetics/Amgen, Inc.

VIKING
The Viking Health Study – Shetland (VIKING) was supported by the MRC Human Genetics Unit quinquennial programme grant “QTL in Health and Disease”. DNA extractions and genotyping were performed at the Edinburgh Clinical Research Facility, University of Edinburgh. We would like to acknowledge the invaluable contributions of the research nurses in Shetland, the administrative team in Edinburgh and the people of Shetland. For the purpose of open access, the author has applied a Creative Commons Attribution (CC BY) licence to any Author Accepted Manuscript version arising from this submission.

TRAILS
TRAILS (TRacking Adolescents' Individual Lives Survey): TRAILS is a collaborative project involving various departments of the University Medical Center and University of Groningen, the University of Utrecht, the Radboud University Medical Center, and the Parnassia Psychiatric Institute, all in the Netherlands. TRAILS has been financially supported by grants from the Netherlands Organization for Scientific Research NWO (Medical Research Council program grant GB-MW 940-38-011; ZonMW Brainpower grant 100-001-004; ZonMw Risk Behavior and Dependence grant 60-60600-97-118;

ZonMw Culture and Health grant 261-98-710; Social Sciences Council medium-sized investment grants GB-MaGW 480-01-006 and GB-MaGW 480-07-001; Social Sciences Council project grants GB-MaGW 452-04-314 and GB-MaGW 452-06-004; ZonMw Longitudinal Cohort Research on Early Detection and Treatment in Mental Health Care grant 636340002, NWO large-sized investment grant 175.010.2003.005; NWO Longitudinal Survey and Panel Funding 481-08-013 and 481-11-001; NWO Vici 016.130.002, 453-16-007/2735, and Vi.C 191.021; NWO Gravitation 024.001.003), the Dutch Ministry of Justice (WODC), the European Science Foundation (EuroSTRESS project FP-006), the European Research Council (ERC-2017-STG-757364 en ERC-CoG-2015-681466), Biobanking and Biomolecular Resources Research Infrastructure BBMRI-NL (CP 32), The Gratema foundation, the Jan Dekker foundation, the participating universities, and Accare Centre for Child and Adolescent Psychiatry. Statistical analyses were carried out on the Genetic Cluster Computer (http://www.geneticcluster.org), which is financially supported by the Netherlands Scientific Organization (NWO 480-05-003) along with a supplement from the Dutch Brain Foundation. We are grateful to all adolescents who participated in this research and to everyone who worked on this project and made it possible.

The Breast Cancer Association Consortium
The BCAC data management was supported by Cancer Research UK grant: PPRPGM-Nov20\100002 and by core funding from the NIHR Cambridge Biomedical Research Centre (NIHR203312) [*]. *The views expressed are those of the author(s) and not necessarily those of the NIHR or the Department of Health and Social Care. The breast cancer genome-wide association analyses were supported by Cancer Research UK (C1287/A10118, C1287/A16563, C1287/A10710), the PERSPECTIVE project via the Government of Canada through Genome Canada and the Canadian Institutes of Health Research (grant GPH[1]129344), the 'Ministère de l'Économie, de la Science et de l'Innovation du Québec' through Genome Québec and grant PSR-SIIRI701, the Quebec Breast Cancer Foundation, the National Institutes of Health (U19 CA148065, CA128978, X01HG007492) and Post-Cancer GWAS initiative (1U19 CA148537, 1U19 CA148065 and 1U19 CA148112 - the GAME-ON initiative), the Department of Defence (W81XWH-10-1-0341), the Canadian Institutes of Health Research (CIHR) for the CIHR Team in Familial Risks of Breast Cancer, Komen Foundation for the Cure, the Breast Cancer Research Foundation, the European Community´s Seventh Framework Programme (HEALTH-F2-2009-223175), and European Union's Horizon 2020 Research and Innovation Programme (grants 634935 and 633784 for BRIDGES and B-CAST respectively). BCAC is additional funded by the Confluence project which is funded with intramural funds from the National Cancer Institute Intramural Research Program, National Institutes of Health, the European Union's Horizon 2020 Research and Innovation Programme (grant numbers 634935 and 633784 for BRIDGES and B-CAST respectively), and the PERSPECTIVE I&I project, funded by the Government of Canada through Genome Canada and the Canadian Institutes of Health Research, the Ministère de l'Économie et de l'Innovation du Québec through Genome Québec, the Quebec Breast Cancer Foundation. The EU Horizon 2020 Research and Innovation Programme funding source had no role in study design, data collection, data analysis, data interpretation or writing of the report. All studies and funders are listed Michailidou et al (2017) [PMID 29059683].

CPS-II: The authors express sincere appreciation to all Cancer Prevention Study-II participants, and to each member of the study and biospecimen management group.

The authors would also like to acknowledge the contribution to this study from central cancer registries supported through the Centers for Disease Control and Prevention's National Program of Cancer Registries and cancer registries supported by the National Cancer Institute's Surveillance Epidemiology and End Results Program. The American Cancer Society funds the creation, maintenance, and updating of the Cancer Prevention Study-II cohort (and/or Cancer Prevention Study-3).

BCINIS: The BCINIS study in Northern Israel is a population-based case-control study which finished 23 years of recruitment of more than 12000 women with incident breast cancer (and a similar amount of matched population controls) who underwent a deep epidemiological and germline/somatic genetic evaluation and clinical follow-up after signing an informed consent. Much of the work was funded by support from the BCRF (Breast Cancer Research Foundation, NYC, USA). This long-term effort could not have done without the help over years of Drs. Flavio Lejbkowicz, Mila Pinchev, Ronit Almog, Meira Frank, Vered Friedman , Shiri Kalet , Esti Liani , Ilana Cohen, Leon Raskin, Marcelo Lau, Leah Naghi, Keren Landsman, Kati Shulman and Hedy S Rennert in the analytical team. It is important to also acknowledge the many dozens of medical oncologists, surgeons, gynecologists an family physicians as well as administrative and technical support teams without whom this type of study could never have taken place.

OCAC
The Ovarian Cancer Association Consortium is supported by a grant from the Ovarian Cancer Research Fund thanks to donations by the family and friends of Kathryn Sladek Smith (PPD/RPCI.07). The scientific development and funding for this project were in part supported by the US National Cancer Institute GAME-ON Post-GWAS Initiative (U19-CA148112). This study made use of data generated by the Wellcome Trust Case Control consortium that was funded by the Wellcome Trust under award 076113. The results published here are in part based upon data generated by The Cancer Genome Atlas Pilot Project established by the National Cancer Institute and National Human Genome Research Institute (dbGap accession number phs000178.v8.p7).

The OCAC OncoArray genotyping project was funded through grants from the U.S. National Institutes of Health (CA1X01HG007491-01 (C.I.A.), U19-CA148112 (T.A.S.), R01-CA149429 (C.M.P.) and R01-CA058598 (M.T.G.); Canadian Institutes of Health Research (MOP-86727 (L.E.K.) and the Ovarian Cancer Research Fund (A.B.). The COGS project was funded through a European Commission's Seventh Framework Programme grant (agreement number 223175 - HEALTH-F2-2009-223175).

23andMe
We thank the research participants and employees of 23andMe for making this work possible.

The Biobank Japan
We would express our sincere gratitude to the study participants, the research and medical staffs in the BBJ. This study was supported by the BioBank Japan project, which was supported by the Ministry of Education, Culture, Sports, Sciences and Technology of the Japanese Government and AMED under grant numbers 17km0305002 and 18km0605001. This study was supported by JSPS KAKENHI

(JP20H00462) from the Ministry of Education, Culture, Sports, Science and Technology of Japan and Japan Agency for Medical Research and Development (AMED) (JP21kk0305013, JP21tm0424220 and JP21ck0106642).

China Kadoorie Biobank
CKB gratefully acknowledges the participants in the study and the members of the survey teams in each of the 10 regional centres, and to the project development and management teams based at Beijing, Oxford and the 10 regional centres. China's National Health Insurance provides electronic linkage to all hospital treatments. The CKB baseline survey and the first re-survey were supported by the Kadoorie Charitable Foundation in Hong Kong. Long-term follow-up was supported by the Wellcome Trust (212946/Z/18/Z, 202922/Z/16/Z, 104085/Z/14/Z, 088158/Z/09/Z), the National Key Research and Development Program of China (2016YFC0900500, 2016YFC0900501, 2016YFC0900504, 2016YFC1303904), and the National Natural Science Foundation of China (81941018, 82192900, 91843302, 91846303). DNA extraction and genotyping was funded by GlaxoSmithKline, and the UK Medical Research Council (MC-PC-13049, MC-PC-14135). The project is supported by core funding from the UK Medical Research Council (MC_UU_00017/1, MC_UU_12026/2, MC_U137686851), Cancer Research UK (C16077/A29186; C500/A16896), and the British Heart Foundation (CH/1996001/9454) to the Clinical Trial Service Unit and Epidemiological Studies Unit and to the MRC Population Health Research Unit at Oxford University. Computation used the Oxford Biomedical Research Computing (BMRC) facility, a joint development between the Wellcome Centre for Human Genetics and the Big Data Institute supported by Health Data Research UK and the NIHR Oxford Biomedical Research Centre.

Danish Blood Donor Study
Funding: The initiation of the Danish Blood Donor Study was supported by the Danish Administrative Regions (02/2611) and the Danish Council for Independent Research (09–069412). The study is currently funded by the Danish Administrative Regions and Bio- and Genome Bank Denmark.
Acknowledgements: We thank the Danish blood donors for their valuable participation in the Danish Blood Donor Study as well as the staff at the blood centers making this study possible.

MoBa
The Norwegian Mother, Father and Child Cohort Study (MoBa) is a population-based pregnancy cohort study conducted by the Norwegian Institute of Public Health. Participants were recruited from all over Norway from 1999-2008. The women consented to participation in 41% of the pregnancies. The cohort includes approximately 114.500 children, 95.200 mothers and 75.200 fathers. The current study is based on version 10 of the quality-assured data files released for research in Novel Tools for Early Childhood Predisposition to Obesity. The establishment of MoBa and initial data collection was based on a license from the Norwegian Data Protection Agency and approval from The Regional Committees for Medical and Health Research Ethics. The MoBa cohort is currently regulated by the Norwegian Health Registry Act. The current study was approved by The Regional Committees for Medical and Health Research Ethics (no. 2012/67). The Medical Birth Registry (MBRN) is a national health registry containing information about all births in Norway.

We thank the Norwegian Institute of Public Health (NIPH) for generating high-quality genomic data. This research is part of the HARVEST collaboration, supported by the Research Council of Norway (#229624). We also thank the NORMENT Centre for providing genotype data, funded by the Research Council of Norway (#223273), South East Norway Health Authorities and Stiftelsen Kristian Gerhard Jebsen. We further thank the Center for Diabetes Research, the University of Bergen for providing genotype data and performing quality control and imputation of the data funded by the ERC AdG project SELECTionPREDISPOSED, Stiftelsen Kristian Gerhard Jebsen, Trond Mohn Foundation, the Research Council of Norway, the Novo Nordisk Foundation, the University of Bergen, and the Western Norway Health Authorities.

Supported by grants (to P.R.N.) from the European Research Council (AdG #293574),the Trond Mohn Research Foundation (TMS2022TMT01), the University of Bergen, the Research Council of Norway (FRIPRO grant #240413), the Western Norway Regional Health Authority (Strategic Fund “Personalized Medicine for Children and Adults”), the Novo Nordisk Foundation (grant #54741), and the Norwegian Diabetes Association; and (to S.J.) Helse Vest’s Open Research Grant (grant #912250), the Research Council of Norway (FRIPRO grant #315599), and Novo Nordisk Foundation (grant #NNF21OC0070349 . This work was partly supported by the Research Council of Norway through its Centres of Excellence funding scheme (#262700), Better Health by Harvesting Biobanks (#229624) and The Swedish Research Council, Stockholm, Sweden (2015-02559), The Research Council of Norway, Oslo, Norway (FRIMEDBIO #547711, March of Dimes (#21-FY16-121). The Norwegian Mother, Father, and Child Cohort Study is supported by the Norwegian Ministry of Health and Care Services and the Ministry of Education and Research, NIH/NIEHS (contract no N01-ES-75558), NIH/NINDS (grant no.1 UO1 NS 047537-01 and grant no.2 UO1 NS 047537-06A1).

Data from the Norwegian Mother, Father and Child Cohort Study and the Medical Birth Registry of Norway used in this study are managed by the national health register holders in Norway (Norwegian Institute of public health) and can be made available to researchers, provided approval from the Regional Committees for Medical and Health Research Ethics (REC), compliance with the EU General Data Protection Regulation (GDPR) and approval from the data owners. The consent given by the participants does not open for storage of data on an individual level in repositories or journals. Researchers who want access to data sets for replication should apply through helsedata.no. Access to data sets requires approval from The Regional Committee for Medical and Health Research Ethics in Norway and an agreement with MoBa.

Analyses were performed using digital laboratories in HUNT Cloud at the Norwegian University of Science and Technology, Trondheim, Norway. We are grateful for outstanding support from the HUNT Cloud community. The participating families who contributed with data and biological material are gratefully acknowledged.

Additional acknowledgements

This research has been conducted using the UK Biobank Resource under Application Number 9905. We thank the KoGES study for providing data.

Additional funding information

KAK, LRK, SS, EJG, RYJ, YZ, FRD, JRBP and KKO are supported by the UK Medical Research Council (MRC; Unit programme MC_UU_00006/2). ISF is supported by funding from Wellcome (207462/Z/17/Z), the National Institute for Health and Care Research (NIHR) Cambridge Biomedical Research Centre, a NIHR Senior Investigator Award, the Botnar Foundation, and the Bernard Wolfe Health Neuroscience Endowment. S. Stankovic is supported by the Clare Hall Ivan D. Jankovic PhD scholarship from the University of Cambridge. ASB is funded by the Deutsche Forschungsgemeinschaft (DFG, German Research Foundation) – 464240267. NF Is funded by NIH DK117445, MD012765, HL163972. RM was supported by the Estonian Research Council grant PRG1911. SEM is supported by an Australian National Health and Medical Research Council Investigator Grant (APP1172917). CT is funded by JSPS KAKENHI (JP20H00462) from the Ministry of Education, Culture, Sports, Science and Technology of Japan and Japan Agency for Medical Research and Development (AMED) (JP21kk0305013, JP21tm0424220 and JP21ck0106642). VV and CH are funded by MRC University Unit Programme Grant "QTL in Health and Disease" (U. MC_UU_00007/10). GWM is supported by an Australian National Health and Medical Research Council Investigator Grant (APP1177194). DIB is funded by Royal Netherlands Academy of Arts and Sciences (KNAW PAH/6635). TE was supported by the Estonian Research Council grant PRG1291. DAL is supported by the British Heart Foundation (AA/18/1/34219 and CH/F/20/90003), the European Research Council under the European Union's Horizon 2020 research and innovation program (grant agreements No 101021566) and UK Medical Research Council (MC_UU_00011/6). NGM is supported by an Australian National Health and Medical Research Council Investigator Grant (APP1172990). NJT is a Wellcome Trust Investigator (202802/Z/16/Z), is the PI of the Avon Longitudinal Study of Parents and Children (MRC & WT 217065/Z/19/Z), is supported by the University of Bristol NIHR Biomedical Research Centre (BRC-1215-2001), the MRC Integrative Epidemiology Unit (MC_UU_00011/1) and works within the CRUK Integrative Cancer Epidemiology Programme (C18281/A29019). SFAG is supported by the Daniel B. Burke Endowed Chair for Diabetes Research, R01 HD056465 and R01 HG010067. MV is supported by the Research Council of Norway (project #301178) and the University of Bergen. FS is supported by the Swiss National Science Foundation (310030_185292) and Novartis Foundation for medical-biological research (18A052).
