# Supplementary material for: Understanding the genetic complexity of puberty timing across the allele frequency spectrum
Source: Nat Genet. 2024 Jul 1;56(7):1397–411. doi: 10.1038/s41588-024-01798-4 (PMC11250262; doi:10.1038/s41588-024-01798-4)
Supplement: Supplementary file 2 — Reporting Summary [file 41588_2024_1798_MOESM2_ESM.pdf]

nature portfolio

Corresponding author(s): Ong KK

Last updated by author(s): Apr 22, 2024

Reporting Summary

Nature Portfolio wishes to improve the reproducibility of the work that we publish. This form provides structure for consistency and transparency in reporting. For further information on Nature Portfolio policies, see our Editorial Policies and the Editorial Policy Checklist.

Statistics

For all statistical analyses, confirm that the following items are present in the figure legend, table legend, main text, or Methods section.

n/a	Confirmed	
☐	☒	The exact sample size (*n*) for each experimental group/condition, given as a discrete number and unit of measurement
☐	☒	A statement on whether measurements were taken from distinct samples or whether the same sample was measured repeatedly
☐	☒	The statistical test(s) used AND whether they are one- or two-sided<br>*Only common tests should be described solely by name; describe more complex techniques in the Methods section.*
☐	☒	A description of all covariates tested
☐	☒	A description of any assumptions or corrections, such as tests of normality and adjustment for multiple comparisons
☐	☒	A full description of the statistical parameters including central tendency (e.g. means) or other basic estimates (e.g. regression coefficient) AND variation (e.g. standard deviation) or associated estimates of uncertainty (e.g. confidence intervals)
☐	☒	For null hypothesis testing, the test statistic (e.g. *F*, *t*, *r*) with confidence intervals, effect sizes, degrees of freedom and *P* value noted<br>*Give P values as exact values whenever suitable.*
☐	☒	For Bayesian analysis, information on the choice of priors and Markov chain Monte Carlo settings
☐	☒	For hierarchical and complex designs, identification of the appropriate level for tests and full reporting of outcomes
☐	☒	Estimates of effect sizes (e.g. Cohen's *d*, Pearson's *r*), indicating how they were calculated

Our web collection on statistics for biologists contains articles on many of the points above.

Software and code

Policy information about availability of computer code

Data collection	No software used
Data analysis	The meta-analysis was performed using a fixed-effects inverse-variance-weighted model in METAL (https://github.com/statgen/METAL, 2011/03/25). Conditionally independent signals were identified using GCTA (version 1.92.0). All LD calculations were performed in plink (https://www.cog-genomics.org/plink2/, version 1.90b6.18). LDSC-SEG (https://github.com/bulik/ldsc, version 1.0.1) was used to perform tissue enrichment analyses. e- and pQTL colocalisation analyses were performed using the R package “coloc” (version 5.1.087) and SMR-HEIDI (version 0.6886). We used MAGMA (version 1.09), PoPS (https://github.com/FinucaneLab/pops, version 0.2), lassosum (https://github.com/tshmak/lassosum, version 0.4.5), fGWAS (https://github.com/joepickrell/fgwas, version 0.3.632), Signed LD Profile regression (SLDP, https://github.com/yakirr/sldp), generalized additive model for location, scale and shape (GAMLSS, version 5.1-7, via www.gamlss.com) in R (v3.6.1), and STRING (https://string-db.org/, version 12.0). Pathway enrichment analysis were performed using g:Profiler (via the R client “gprofiler2”, version 0.2.1). Plots were created using R (version 4.2.1) using ggplot2 (3.3.6) and the "Zissou1" palette from the wesanderson R package (version 0.3.6). Code for WES data processing and association testing is available on GitHub (https://github.com/mrcepid-rap/mrcepid-runassociationtesting).

For manuscripts utilizing custom algorithms or software that are central to the research but not yet described in published literature, software must be made available to editors and reviewers. We strongly encourage code deposition in a community repository (e.g. GitHub). See the Nature Portfolio guidelines for submitting code & software for further information.

Data

Policy information about availability of data

All manuscripts must include a data availability statement. This statement should provide the following information, where applicable:

- Accession codes, unique identifiers, or web links for publicly available datasets
- A description of any restrictions on data availability
- For clinical datasets or third party data, please ensure that the statement adheres to our policy

Cohorts should be contacted individually for access to their raw data. UK Biobank data are available on application (https://www.ukbiobank.ac.uk/enable-your-research/register). Summary statistics from the meta-analyses, excluding 23andMe, are available via https://doi.org/10.17863/CAM.107943. Access to the full summary statistics including 23andMe results, can be obtained from 23andMe after completion of a Data Transfer Agreement (https://research.23andme.com/dataset-access/).
We used the NCBI RefSeq gene map for GRCh37 which is available via http://hgdownload.soe.ucsc.edu/goldenPath/hg19/database/. GTEx eQTL data was used (V7) and is available via https://gtexportal.org. Genes linked to rare monogenic disorders were annotated from the Online Mendelian Inheritance in Man (OMIM) database (via Online Mendelian Inheritance in Man, OMIM®. McKusick-Nathans Institute of Genetic Medicine, Johns Hopkins University (Baltimore, MD), accessed November 2023. World Wide Web URL: https://omim.org/).

Research involving human participants, their data, or biological material

Policy information about studies with human participants or human data. See also policy information about sex, gender (identity/presentation), and sexual orientation and race, ethnicity and racism.

Reporting on sex and gender	All analyses are disaggregated by sex.<br>All contributing studies had research ethics approval and collected informed written consent.
Reporting on race, ethnicity, or other socially relevant groupings	Study samples were defined by genetic ancestry.
Population characteristics	Described in Supplementary Table 1 "Cohorts"
Recruitment	This study is a meta-analysis of data from a number of sources. Recruitment varied across the different studies that contributed data.
Ethics oversight	UK Biobank data has approval from the North West Multi-centre Research Ethics Committee (MREC) as a Research Tissue Bank (RTB). 23andMe research participants provided informed consent and volunteered to participate in the research online under a protocol approved by the external AAHRPP-accredited IRB, Ethical & Independent (E&I) Review Services. Each of the other individual studies that contributed data has their own ethical approval from the relevant boards.

Note that full information on the approval of the study protocol must also be provided in the manuscript.

Field-specific reporting

Please select the one below that is the best fit for your research. If you are not sure, read the appropriate sections before making your selection.

☒ Life sciences ☐ Behavioural & social sciences ☐ Ecological, evolutionary & environmental sciences

For a reference copy of the document with all sections, see nature.com/documents/nr-reporting-summary-flat.pdf

Life sciences study design

All studies must disclose on these points even when the disclosure is negative.

Sample size	For GWAS discovery analyses, we meta-analysed data from all large-scale Biobanks and consortia with puberty data (n=799,845). For whole exome sequencing discovery analyses, we used the full available sample with available data in UK Biobank (n=222,283).
Data exclusions	Only individuals failing standard genotyping quality control parameters defined in the individual studies or missing genotype, phenotype, or covariate data were excluded from analysis. This decision was made prior to performing any downstream analysis. Where described, sensitivity analyses were performed in subsets of the UK Biobank cohort, with exclusions of related individuals and/or non-European ancestry individuals.
Replication	We replicated our GWAS findings using menarche association data from the Danish Blood Donors study (n=35,467) and the ALSPAC study (n=3,140). Menarche ExWAS and GWAS data were also replicated using data on voice-breaking (n=178,625 and up to n=205,354, accordingly). All attempted replication has been reported in the manuscript without exception.
Randomization	The principle exposure in this study is naturally occurring genetic variants, meaning that we were unable to randomize the individuals in the study. To account for possible confounding, we used a linear mixed model and adjusted for technical and demographic covariates.

Blinding	Blinding is not applicable to this study, as it is a genome-wide association study of common and rare genetic variation and not a randomized controlled trial. We did not deliver any intervention to the participants in this study.

Reporting for specific materials, systems and methods

We require information from authors about some types of materials, experimental systems and methods used in many studies. Here, indicate whether each material, system or method listed is relevant to your study. If you are not sure if a list item applies to your research, read the appropriate section before selecting a response.

Materials & experimental systems

n/a	Involved in the study
☒	☐ Antibodies
☐	☒ Eukaryotic cell lines
☒	☐ Palaeontology and archaeology
☒	☐ Animals and other organisms
☒	☐ Clinical data
☒	☐ Dual use research of concern
☒	☐ Plants

Methods

n/a	Involved in the study
☒	☐ ChIP-seq
☒	☐ Flow cytometry
☒	☐ MRI-based neuroimaging

Eukaryotic cell lines

Policy information about cell lines and Sex and Gender in Research

Cell line source(s)	HEK293 cells were kindly provided by Professor Michel Bouvier, Universite de Montreal, Canada.
Authentication	HEK293 cells were authenticated via GENETICA Genotypes Analysis in May 2019, showing 97% match when compared to the reference profile ATCC sequence.
Mycoplasma contamination	HEK293 cells tested negative for mycoplasma contamination using MycoProbe Mycoplasma Detection Kit (CUL001B, R&D Systems).
Commonly misidentified lines (See ICLAC register)	This is not a commonly misidentified cell line, as listed on the ICLAC register (version 12).

Plants

Seed stocks	NA
Novel plant genotypes	NA
Authentication	NA
